# Lactylation-dependent DDX18 nucleolar escape regulates *CD44* mRNA stability to drive fibrotic progression in kidney injury

**DOI:** 10.1172/JCI201848

**Published:** 2026-08-13

**Authors:** Lijun Dong, Jingwen Xie, Mengyuan Tao, Shuai Liu, Yueyang Lu, Tianxing Wu, Jian Geng, Qingyun Chen, Xiaoshan Zhao, Jianbo Zhao, Jia Zhou, Honghao Hou, Jun Ai, Tao Tao, Daming Zuo

**Affiliations:** 1 Division of Vascular and Interventional Radiology, Department of General Surgery, Nanfang Hospital,; 2 Institute of Molecular Immunology, Guangdong Province Key Laboratory of Immune Regulation and Immunotherapy, Key Laboratory of Infectious Diseases Research in South China, Ministry of Education, School of Laboratory Medicine and Biotechnology,; 3 Department of Immunology, Guangdong Province Key Laboratory of Proteomics, School of Basic Medical Sciences,; 4 Guangdong Provincial Key Laboratory of Construction and Detection in Tissue Engineering, School of Basic Medical Sciences,; 5 Department of Pathology, Nanfang Hospital,; 6 Institute of Medical Research, Guangdong Provincial People’s Hospital (Guangdong Academy of Medical Sciences),; 7 School of Traditional Chinese Medicine,; 8 Department of General Practice, Nanfang Hospital, and; 9 Sleep and Brain Medicine Center, Shenzhen Hospital, Southern Medical University, Shenzhen, Guangdong, China.

**Keywords:** Cell biology, Nephrology, Chronic kidney disease, Protein traffic, RNA processing

## Abstract

Following acute kidney injury (AKI), a substantial subset of patients experience an irreversible progression to chronic kidney disease (CKD), yet the molecular determinants governing this maladaptive transition remain elusive, and effective clinical interventions are lacking. Here, we identify lactate as a key metabolic determinant orchestrating the transition from AKI to CKD. Analysis of the UK Biobank cohort revealed that elevated circulating lactate independently predicts CKD development in patients with AKI and correlates with fibrotic progression. Using murine ischemia-reperfusion injury models, we demonstrated that lactate drives sustained renal damage through posttranslational lactylation of the RNA helicase DDX18. Mechanistically, p300-mediated lactylation of DDX18 at lysine 116 disrupted its nucleolar retention, causing redistribution to the nucleoplasm, where it acquired enhanced binding affinity for *CD44* mRNA. This subcellular relocalization stabilized *CD44* mRNA through altered RNA–protein interactions, thereby amplifying fibrotic signaling pathways. Therapeutically, we developed a kidney-targeted, cell-penetrating peptide that specifically inhibits DDX18 K116 lactylation, effectively attenuating fibrotic progression in injured kidneys. Our findings establish protein lactylation as a regulatory mechanism governing RNA helicase nucleolar localization and subsequent control of mRNA stability, revealing a potential therapeutic target for interrupting fibrotic processes in CKD.

## Graphical abstract

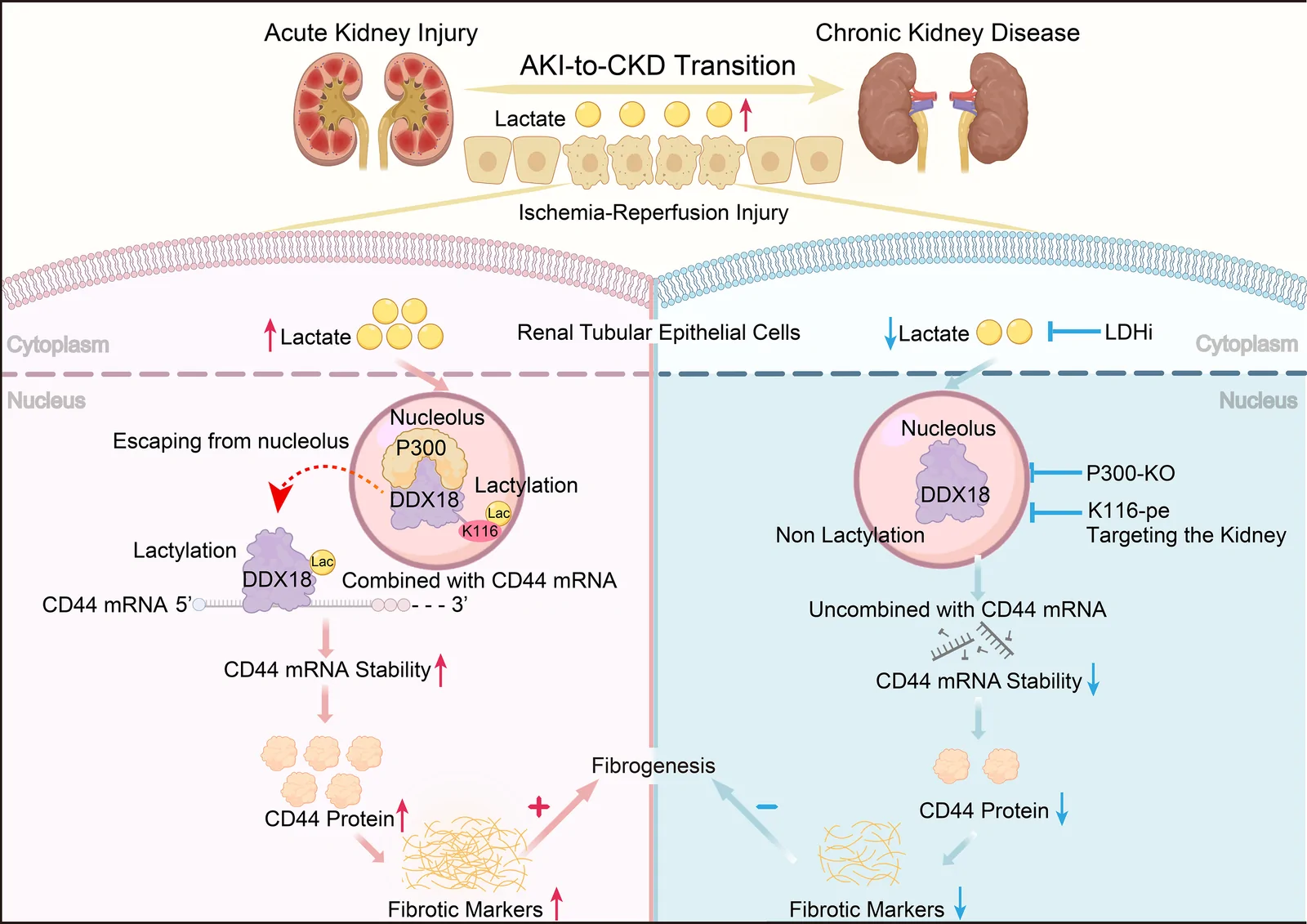

## Introduction

Kidney disease encompasses acute and chronic disorders that progressively impair renal function. Acute kidney injury (AKI), a common clinical syndrome associated with substantial morbidity and mortality, is caused by ischemia, nephrotoxins, and sepsis-related inflammation (1–3). Emerging evidence reveals that AKI often initiates complex pathophysiological cascades with diverse clinical trajectories, including complete recovery, partial resolution, recurrent episodes, or progression to chronic kidney disease (CKD). The AKI-to-CKD transition is a critical event in the patient’s clinical trajectory and is characterized by persistent inflammation, tubular atrophy, interstitial fibrosis, and progressive loss of renal function (4, 5). Understanding the molecular mechanisms governing this transition remains a critical clinical challenge.

Tubulointerstitial fibrosis is the common pathway driving kidney dysfunction, characterized by pathological accumulation of ECM components (6, 7). Beyond structural support, ECM functions as a platform regulating cellular behavior (8). During AKI-to-CKD transition, excessive ECM synthesis and impaired degradation cause pathological deposition of collagens, fibronectin, and proteoglycans (7, 9), whereas dysregulated remodeling enzymes disrupt matrix architecture and drive fibrotic progression (6, 8). Central to this process are renal tubular epithelial cells (TECs), which exhibit remarkable phenotypic plasticity following injury (10, 11). Rather than serving as passive victims or bystanders, TECs actively shape disease trajectories through context-dependent activation of regenerative or maladaptive repair programs (12, 13). However, the molecular mechanisms governing TEC fate decisions in CKD remain incompletely understood.

RNA binding proteins (RBPs) are posttranscriptional regulators that orchestrate RNA metabolism from processing and localization to stability and translation (14). Accumulating evidence implicates the role of RBPs in AKI, diabetic kidney disease, and glomerular disorders (15). For instance, Hu antigen R promotes septic AKI-to-CKD transition by stabilizing CD147 mRNA (16). Similarly, AU-rich element RNA-binding protein (AUF1) promotes fibrotic progression through destabilization of NEDD4L mRNA and overactivation of TGF-β signaling (17). These findings position RBPs as attractive therapeutic targets, yet how RBP functions during CKD progression remains poorly explored.

Metabolic reprogramming is a fundamental driver of kidney disease progression. Alterations in circulating metabolites, including uric acid, sugars, amino acids, lipids, and organic acids, correlate with and potentially predict CKD onset and progression (18–20). However, the metabolic mechanisms linking AKI to fibrosis remain unclear. Our biobank cohort analysis identified lactate as a key metabolic driver of kidney fibrotic progression following AKI, with elevated circulating lactate independently predicting subsequent CKD development. Using a murine renal ischemia-reperfusion model, lactate was found to drive persistent kidney injury by promoting posttranslational lactylation of RNA helicase DDX18, an RBP of the DEAD-box family. Specifically, p300-mediated lactylation of DDX18 at lysine 116 (K116) triggered its redistribution from the nucleolus to the nucleoplasm, enhancing its binding to and stabilization of *CD44* mRNA and thereby accelerating fibrosis. We further developed a kidney-targeted, cell-penetrating peptide that selectively inhibited DDX18 K116 lactylation and attenuated fibrosis in injured kidneys. These findings define a lactate/DDX18/*CD44* axis linking metabolic reprogramming to maladaptive AKI-to-CKD progression and provide a potential therapeutic strategy for progressive kidney disease.

## Results

### Elevated lactate predicts the progression of AKI to CKD.

To delineate the metabolic factors driving the AKI-to-CKD transition, we performed plasma metabolomic profiling in a longitudinal UK Biobank cohort via Nightingale Health’s NMR platform, focusing on plasma lactate measured in participants with AKI within 1 year preceding recruitment (identified through Hospital Episode Statistics). Patients who progressed to CKD displayed elevated plasma lactate compared with nonprogressing counterparts (Figure 1, A and B). Elevated lactate predicted higher cumulative risk of CKD progression (Figure 1C), independent of initial renal function, as serum creatinine levels were comparable between the low- and high-lactate groups (Supplemental Table 1; supplemental material available online with this article; https://doi.org/10.1172/JCI201848DS1). Parallel proteomic analysis revealed enhanced fibrogenesis in patients progressing from AKI to CKD, characterized by ECM components (Figure 1D). This progression signature featured upregulation of structural ECM proteins, including collagens and MMPs, alongside fibroblast markers such as α-smooth muscle actin (α-SMA; encoded by ACTA2) (Figure 1E). Collectively, these data establish lactate as a potential mechanistic link between metabolic dysregulation and progressive renal fibrogenesis following AKI.

Next, we investigated exogenous lactate administration in a mouse model of bilateral ischemia-reperfusion injury (BIRI) (Figure 1F). Lactate exacerbated kidney dysfunction, increasing the serum levels of creatinine, uric acid, and blood urea nitrogen (BUN) (Figure 1G). Histological analyses revealed tubular damage and interstitial collagen deposition (Figure 1H). Molecular analyses showed increased expression of fibrotic markers, including fibronectin, α-SMA, and collagen type I, at mRNA (Figure 1I) and protein levels (Figure 1, J and K). We next assessed whether lactate accumulation was associated with injury progression. Acute lactate administration transiently raised renal lactate levels (Supplemental Figure 1A). Chronic lactate treatment did not alter renal/plasma lactate levels or plasma pH in sham-operated mice but increased renal/plasma lactate and reduced plasma pH in BIRI mice (Supplemental Figure 1, B–D), indicating selective amplification of injury-associated metabolic disturbances. LDHA and LDHB expression was markedly increased in BIRI kidneys (Supplemental Figure 1, E and F) and in hypoxia-exposed HK-2 cells (Supplemental Figure 1, G–I). Pharmacological inhibition of LDH using (R)-GNE-140 alleviated AKI-to-CKD progression (Figure 1L), reduced kidney dysfunction (Figure 1M), alleviated histopathological damage (Figure 1N), and suppressed renal fibrosis markers (Figure 1, O–Q). Consistently, (R)-GNE-140 also reduced lactate accumulation in BIRI kidneys and TECs (Supplemental Figure 1, J and K) and prevented hypoxia-induced fibrotic responses in vitro (Supplemental Figure 1, L–N). Collectively, these data suggest that lactate accumulation may be involved in the progression from AKI to CKD.

### Aberrant protein lactylation marks injured TECs.

Protein lysine lactylation (Kla), a lactate-derived posttranslational modification, regulates diverse cellular processes (21). We hypothesized that this modification may contribute to the AKI-to-CKD progression. To test this, we stratified human AKI biopsies by proteinuria, a robust indicator of CKD development (Supplemental Table 2) (22). Biopsies from patients with AKI with persistent proteinuria exhibited a marked upregulation of global protein lactylation compared with nonproteinuric controls (Figure 2A). Elevated lactylation was recapitulated in kidney tissue from BIRI mice, predominantly localized to renal TECs (Figure 2B). Lactylation intensity increased progressively after kidney injury (Figure 2C) and was further potentiated by lactate administration (Figure 2D).

Lactylation proteomics using a specific Kla antibody (Supplemental Figure 2) identified 143 proteins with increased Kla modifications and 39 proteins with decreased Kla modifications following hypoxic injury in TECs, revealing 207 upregulated and 73 downregulated lactylation sites (Figure 2, E and F, and Supplemental Figure 3A). Most lactylated proteins localized to the nucleus (Figure 2G) and were implicated in RNA metabolic and processing pathways (Figure 2H and Supplemental Figure 3B). Sequence motif analysis revealed that lactylation sites preferentially cluster near lysine residues (Supplemental Figure 3, C and D). Higher lactylation enrichment was observed particularly in RNA helicase proteins, key regulators of RNA metabolism involved in ribosome biogenesis, translation, RNA export, and stability (23, 24). We focused on DDX18, an ATP-dependent RNA helicase critical for rRNA transcription, which presented increased lactylation (Figure 2I and Supplemental Table 3).

Interestingly, renal DDX18 expression remained stable in human renal disease (Figure 2J) and mouse kidney injury models (Figure 2K), whereas DDX18 lactylation was increased in BIRI kidneys (Figure 2L), particularly with lactate administration (Figure 2M). In vitro studies corroborated these findings, demonstrating elevated DDX18 lactylation in hypoxia-reoxygenation–treated (H/R-treated) TECs (Figure 2N). Thus, DDX18 lactylation, rather than expression, appears critical in the transition toward chronic disease states.

### DDX18 orchestrates fibrogenic processes during the progression of kidney disease.

To investigate the role of TEC-expressed DDX18 during AKI-to-CKD progression, TEC-specific *DDX18*-knockout mice were generated via a *Ksp* promoter–driven AAV9 vector (Figure 3A). DDX18 deletion was restricted to the kidneys, with no off-target reduction in other tissues (Supplemental Figure 4). TEC-specific DDX18 depletion protected against BIRI-induced renal dysfunction and structural deterioration, with reduced tubular injury and interstitial collagen deposition (Figure 3, B and C). Protection was observed in both male and female mice (Supplemental Figure 5). DDX18 deficiency blunted the upregulation of profibrotic markers at both the transcript and protein levels following BIRI (Figure 3, D–F). RNA-seq analysis of microdissected tubulointerstitial tissue from patients with kidney disease (Gene Expression Omnibus [GEO]: GSE175759) revealed a positive correlation between *DDX18* expression and fibrotic gene signatures (Supplemental Figure 6), supporting a role for DDX18 in human fibrotic progression. To further elucidate the role of DDX18 in fibrogenesis, *DDX18* was silenced in hypoxia-challenged TECs. DDX18 depletion attenuated hypoxia-induced TEC migration (Figure 3G), accompanied by reduced fibrotic marker expression (Figure 3, H–J). Together, these findings identify DDX18 as a critical regulator of fibrogenic reprogramming during kidney disease progression.

### DDX18 lactylation at K116 mediates its profibrogenic function.

Lactylation occurs on specific lysine residues that are essential for protein folding and function (25). Structural mapping of DDX18 identified 5 conserved lysine residues (Figure 4A), among which K116 exhibited the hypoxia-induced lactylation in injured TECs (Figure 4B), within the missed-cleavage peptide KMVNDAEPDTK. To functionally validate this site, we generated a lysine-to-arginine substitution series (K→R mutants). Compared with the DDX18^WT^ and other mutants, the DDX18^K116R^ mutant presented decreased DDX18 lactylation (Figure 4C), identifying K116 as the major lactylation site. K116 is highly conserved from *Danio rerio* to *Homo sapiens* (Figure 4D).

We then generated an antibody against DDX18 lactylated at K116 (DDX18-K116la) (Figure 4E and Supplemental Figure 7). DDX18 K116 lactylation was increased in TECs treated with lactate (Figure 4F) or in response to hypoxic conditions (Figure 4G). DDX18 K116 lactylation in TECs induced by lactate treatment was dose dependent (Supplemental Figure 8A) and time dependent (Supplemental Figure 8B). In addition, serum starvation, CoCl_2_, or cisplatin treatment increased DDX18 K116 lactylation in TECs (Supplemental Figure 8, C–E). Conversely, blunting lactate metabolism with the LDH inhibitor oxamate or the pyruvate dehydrogenase activator dichloroacetate attenuated DDX18 K116 lactylation (Supplemental Figure 8, F and G). Increased K116 lactylation was seen in primary TECs from BIRI mice (Figure 4H), proteinuric versus nonproteinuric AKI kidneys (Figure 4I), and AKI-to-CKD mice (Figure 4J). Reconstituting DDX18-deficient cells with the DDX18^WT^ or DDX18^K116R^ mutant showed that DDX18^K116R^ cells had lower migratory capacity (Figure 4K) and less fibrotic marker levels (Figure 4, L–N) than DDX18^WT^ cells, indicating DDX18 K116 lactylation drives TEC fibrogenesis under pathological conditions.

### p300 mediates DDX18 lactylation to drive fibrotic signaling in injured TECs.

To identify potential lactylation regulators of DDX18, we performed IP coupled with mass spectrometry (MS) in TECs (Supplemental Figure 9A). Among the identified proteins, the established lactyltransferases AARS1 and p300 have emerged as promising candidates (Supplemental Figure 9B). Co-IP confirmed an interaction of DDX18 and p300, but not AARS1 (Supplemental Figure 9, C and D, and Figure 5A), which increased following BIRI injury (Figure 5B), coinciding with H/R-induced p300 upregulation in HK-2 cells (Figure 5C). p300 overexpression increased DDX18 K116 lactylation under H/R conditions without altering total DDX18 levels (Figure 5D). Conversely, CRISPR/Cas9-mediated p300 knockdown attenuated DDX18 K116 lactylation in injured TECs (Figure 5E). P300 deficiency eliminated lactylation differences between DDX18^WT^- and DDX18^K116R^-expressing cells (Figure 5F), confirming p300 as the primary enzyme mediating this modification. We then analyzed whether the nucleolar localization of DDX18 depends on p300. p300 depletion impaired DDX18 translocation from the nucleolus to the nucleoplasm following H/R stimulation (Figure 5G) and abrogated the distinct fibrotic responses of DDX18^WT^- and DDX18^K116R^-expressing cells (Figure 5, H–J). The p300 inhibitor C646 similarly abolished differences in fibrotic marker expression between DDX18^WT^- and DDX18^K116R^-expressing cells (Supplemental Figure 10). These data suggest that metabolic stress promotes p300-mediated DDX18 K116 lactylation, enabling DDX18 to promote fibrotic signaling.

### DDX18 K116 lactylation regulates CD44 mRNA stability.

Given the emerging role of DDX18 in nucleolar liquid–liquid phase separation (26), we explored how lactylation influences its cellular distribution. H/R treatment markedly decreased the intensity of DDX18 condensates within HK-2 nucleoli (Figure 6A), whereas DDX18^K116R^ resisted H/R-induced nucleolar displacement (Figure 6B), indicating that K116 lactylation directly regulates DDX18 nucleolar distribution.

RNA immunoprecipitation sequencing (RIP-seq) analysis showed DDX18 targets enriched in RNA metabolic processes (Figure 6C). RNA-seq comparing DDX18^WT^- and DDX18^K116R^-expressing cells identified differential expression of genes involved in cell adhesion, adherens junctions, cell-substrate junctions, and focal adhesions (Figure 6D). Integrating both datasets identified 481 common targets responsive to DDX18 binding and H/R treatment (Figure 6E). Cell adhesion–related genes were downregulated in DDX18^K116R^ cells following H/R exposure (Figure 6F). Among these, CD44, a multifunctional cell surface receptor implicated in cell matrix adhesion (27), showed pronounced downregulation (Figure 6G). CD44 also plays a critical role in modulating fibroblast behavior through regulating ECM expression (28). *CD44* expression positively correlated with fibrotic signatures (Supplemental Figure 11) and *DDX18* expression (Figure 6H) in kidney disease samples (GEO: GSE175759), supporting a functional *DDX18*-*CD44* link in renal fibrosis.

Immunoblotting (Figure 6I) and immunofluorescence (Figure 6J) confirmed reduced CD44 protein in DDX18^K116R^-expressing cells under H/R. CD44 depletion eliminated the differential fibrotic response between DDX18^WT^- and DDX18^K116R^-reconstituted cells (Figure 6, K–M), establishing CD44 as a key downstream effector in DDX18 lactylation–mediated fibrogenesis. RNA stability assays showed that DDX18^K116R^ accelerated *CD44* mRNA degradation compared with DDX18^WT^ (Figure 6N), indicating that K116 lactylation stabilizes *CD44* transcripts. RIP-seq analysis demonstrated enrichment of *CD44* mRNA in DDX18 immunoprecipitates relative to IgG controls (Figure 6O), and deletion mapping confirmed the DDX18-binding region on *CD44* mRNA (Figure 6P). In cells expressing a *CD44* variant lacking this region, differences in *CD44* mRNA stability (Figure 6Q) and protein expression (Figure 6R) between DDX18^WT^- and DDX18^K116R^-overexpressing cells were eliminated. Together, these results establish that the lactylation-induced redistribution of DDX18 from the *CD44* transcript regulates *CD44* mRNA stability, a critical factor driving fibrotic progression in kidney disease.

### Targeted inhibition of DDX18 K116 lactylation mitigates the progression of AKI to CKD in mice.

Because p300 inhibition or blockade of lactate metabolism affects multiple pathways, we sought a selective strategy to inhibit DDX18 K116 lactylation. Synthetic peptides based on posttranslational modification motifs can inhibit specific protein modifications (29). Based on the sequence surrounding DDX18 K116, we designed and synthesized 5 cell-penetrating peptide–conjugated inhibitors (Figure 7A). Among them, DDX18 K116-peptide-2# (hereafter, K116-pe) exhibited the strongest inhibitory effect on lactylation (Figure 7B), a K116R substitution served as the specificity control (K116R-pe). K116-pe attenuated the hypoxia-induced expression of fibrosis-related proteins in TECs (Figure 7, C–E), supporting its therapeutic potential.

We then engineered a kidney-targeted delivery platform using l-serine–modified poly(lactic-co-glycolic acid) (PLGA) to facilitate the selective renal delivery of K116-pe in BIRI mice (Figure 7F and Supplemental Figure 12). Biodistribution studies using mCherry-labeled carriers demonstrated the preferential renal accumulation and extended retention of l-serine–modified PLGA-K116-pe (PLGA-Ser-MOF-K116-pe) (Figure 7G), with tubule-specific localization verified by immunofluorescence and electron microscopy (Supplemental Figure 13). The delivery system showed a favorable safety profile, with no effects on survival or organ function (Supplemental Figure 14). BIRI mice treated with PLGA-Ser-MOF-K116-pe, but not the K116R control, on day 1 after surgery showed alleviated AKI-to-CKD transition (Figure 7, H–J), an effect reproduced in female mice (Supplemental Figure 15). PLGA-Ser-MOF-K116-pe treatment markedly decreased renal fibrogenic progression in BIRI mice (Figure 7, K–M) and reduced CD44 expression in the kidneys of BIRI mice (Figure 7, N and O). Together, these results suggest that the selective inhibition of DDX18 K116 lactylation represents a promising therapeutic strategy to mitigate AKI-to-CKD progression.

## Discussion

The pathological transition from AKI to CKD is a major clinical challenge, predominantly driven by maladaptive molecular crosstalk within TECs (4). Here, we identify a lactate-dependent regulatory axis in which p300-mediated lactylation of DDX18 at K116 promotes its nucleolar redistribution, enhances *CD44* mRNA stability, and activates profibrotic programs in TECs. Renal system–targeted delivery of cell-penetrating lactylation inhibitors using l-serine–functionalized PLGA nanoparticles reduced DDX18 K116 lactylation and attenuated AKI-to-CKD progression in preclinical models.

The kidney plays a crucial role in gluconeogenesis, with lactate as the primary substrate under physiological conditions (30). Elevated lactate is an independent risk factor for patients with sepsis-induced AKI (31). Both renal glucose release and lactate uptake are diminished in human and experimental AKI, correlating with increased mortality (32). A previous clinical cohort study linked urinary lactate to tubular injury biomarkers and albuminuria (33), while serum lactate dehydrogenase/albumin ratio predicts poor AKI prognosis (34). Our biobank analysis extends these findings, showing that elevated serum lactate independently predicts CKD progression after AKI. In murine BIRI, exogenous lactate accelerated kidney dysfunction, whereas inhibiting lactate production attenuated fibrosis. TECs have high metabolic demands and are particularly susceptible to injury during AKI (10). While transient TEC migration is a physiologically essential feature of early epithelial repair (35, 36), our findings indicate that enhanced TEC migration is accompanied by increased expression of profibrotic mediators and ECM proteins. Whether hypermigration itself directly contributes to fibrotic progression or instead represents a parallel consequence of broader maladaptive epithelial reprogramming remains to be determined. Recent studies reported elevated lactate promotes fibrogenic responses in DKD mice and advanced glycation end products–simulated (AGEs-stimulated) TECs (37). Our findings reveal a context-dependent role of lactate during AKI progression. Systemic lactate in sham mice did not alter baseline function, but post-ischemic lactate accumulation exacerbated fibrosis, indicating that lactate acts as a disease-associated signaling molecule amplifying injury rather than a primary nephrotoxin.

RNA helicases regulate diverse cellular processes through interactions among RNA, DNA, and proteins (24, 38). Our proteomic analysis revealed extensive lactylation of nuclear RNA helicases in injured TECs. Lactate has emerged as an important signaling molecule that regulates cellular phenotypes through lysine lactylation (39). Wang et al. demonstrated that lactate promotes kidney fibrosis through histone lactylation–mediated NF-κB activation (40). In contrast, our study identifies a complementary posttranscriptional mechanism via nonhistone DDX18 lactylation, suggesting that histone and nonhistone lactylation cooperate during AKI-to-CKD transition. Protein lactylation also contributes to tubular inflammation, cell death, and fibrosis (41), representing an upstream link between metabolic stress and fibrogenic signaling (42). DDXs promote RNA processing and are essential for rearranging RNA structures to promote RNA–protein interactions in an ATP-dependent manner (43). They are crucial for genome protection, with dysfunction implicated in cancer development, viral infections, and tissue differentiation (24, 44, 45). DDX24 stabilizes Fanconi anemia complementation group A mRNA to maintain cell cycle progression and DNA repair (46). DDX18 interacts with RNA/DNA hybrids to maintain genome integrity (47, 48). Additionally, DDX18 enhances STAT1 expression, leading to increased PD-L1 expression (49). Our study demonstrates that lactylation modulates the RNA-binding properties of DDX18, expanding understanding of posttranslational regulation of DDX function. Hypoxia-treated immortalized TECs enabled detailed analysis of DDX18 lactylation and RNA interactions but did not fully reproduce the metabolic complexity of ischemic kidney injury, including acidosis and the accumulation of multiple intermediates. Future studies using kidney organoids or other physiologically relevant models are needed to refine these findings.

Progressive fibrosis, a hallmark of AKI-to-CKD transition, is characterized by excessive ECM production and dysregulated turnover (9). CD44 is a multifunctional receptor integrating extracellular signals with the growth factor pathway (28, 50). Upon activation, this transmembrane receptor transduces signals via membrane-associated cytoskeletal proteins to drive transcriptional programs governing cell matrix adhesion, migration, proliferation, differentiation, and survival (27, 28). In renal diseases, CD44 is markedly increased on injured TECs, contributing to tubular damage, apoptosis, and fibrosis (51–53), partly through HGF and TGF-β signaling pathways (52, 54). Our data establish CD44 as a downstream effector of lactylated DDX18. Mechanistically, lactylated DDX18 directly binds to and stabilizes *CD44* mRNA, boosting its expression and driving profibrotic responses in TECs. Although RIP-seq confirmed this interaction, the precise binding motifs remain unknown, and this mechanism links altered lactate metabolism to RNA regulation and ECM remodeling.

Protein lactylation is catalyzed by lactyltransferases including p300, GCN5, TIP60, and KAT8 (21), as well as the alanyl-tRNA synthetases AARS1 and AARS2 (55–57). We demonstrated that p300 directly lactylates DDX18 and that lactate facilitates this modification by strengthening the p300–DDX18 interaction. p300 has been reported to acetylate both free and nucleosomal histones (58). P300-mediated acetylation of copper metabolism murr1 domain 1 triggers RelA ubiquitylation and nucleolar translocation (59). Similarly, acetylation of the WRN helicase by p300 promotes its translocation from the nucleolus to nucleoplasmic foci (60). Our findings extend this paradigm by demonstrating that p300-mediated lactylation dictates the nucleolar-nucleoplasmic shuttling of DDX18, revealing a mechanism regulating RNA helicase localization and function. Pharmacological inhibition of p300 reduced DDX18 lactylation and attenuated hypoxia-induced profibrotic responses in TECs. Cell-penetrating peptides targeting DDX18 K116 lactylation inhibited TEC fibrogenic activation. To improve renal delivery, we engineered l-serine–functionalized PLGA-MOF nanoparticles. Nanoparticle biodistribution is influenced by physicochemical properties, including particle size (61–63). The PLGA-Ser-MOF base framework measured approximately 65 nm, while the mCherry-conjugated construct measure approximately 70 nm, both within the mesangial targeting range. These properties support preferential renal accumulation and TEC uptake, thereby attenuating renal fibrosis in vivo. Although AAV9 exhibits broad tissue tropism, Cas9 expression in our study was restricted to renal TECs by the Ksp-cadherin promoter. Therefore, the kidney-specific reduction of DDX18 reflects promoter-driven control of Cas9 activity rather than differences in editing efficiency between kidney and liver tissues.

In summary, DDX18 K116 lactylation acts as a pivotal regulator of AKI-to-CKD progression. Lactate accumulation and p300-dependent lactylation trigger DDX18 nucleolar redistribution, enhance *CD44* mRNA stability, and drive ECM remodeling and fibrosis. Peptide-mediated targeting of DDX18 lactylation significantly reduces renal fibrosis in preclinical models. These findings identify lactylation-mediated regulation of RNA-helicase function as a potential link between metabolic stress and fibrotic responses and warrant investigation in other fibrotic diseases.

## Methods

### Sex as a biological variable.

Our study examined male and female animals, and similar findings are reported for both sexes.

### Prospective cohort study in the UK Biobank.

We analyzed UK Biobank data from approximately 500,000 participants aged 40–69 years recruited across the United Kingdom between 2006 and 2010, with linked hospital, mortality, and baseline omics records. AKI and CKD were identified using ICD-10 codes N17 and N18, respectively. Participants with prerecruitment AKI were followed for CKD progression, defined as the first postrecruitment N18 diagnosis. Those with CKD before or at recruitment were excluded, and participants without subsequent CKD formed the nonprogression group. Baseline EDTA plasma underwent proteomic and metabolomic profiling. The Olink Explore 3,072 platform, covering cardiometabolic, inflammation, neurology, and oncology panels, was used for proteomics. Randomized samples were analyzed by proximity extension assay, sequenced on a NovaSeq 6000 system (Illumina, Inc.), and processed into quality-controlled, rank-based inverse-normal-transformed NPX values (64). Metabolomics was performed on 118,019 baseline samples using Nightingale Health’s NMR platform and a 500 MHz Bruker AVANCE IIIHD spectrometer, quantifying 249 biomarkers. Quality control included duplicates, coefficients of variation < 5% for most biomarkers, and cross-batch consistency checks. Participants were stratified by median lactate into low (≤4.135 mmol/L) and high (>4.135 mmol/L) groups, and baseline NMR-measured creatinine was compared. Cox models estimated adjusted hazard ratios for CKD progression, controlling for age, sex, body mass index, smoking, and alcohol intake. Kaplan-Meier curves were compared by log-rank test. All participants provided informed consent. Ethical approval was granted by the North West Research Ethics Committee (06/MRE08/65), and data were accessed under UKB application 90369.

### Patient samples.

Human kidney biopsy specimens were obtained from Nanfang Hospital, Southern Medical University. Tissues were formaldehyde fixed and paraffin embedded using standard procedures. The study was approved by the Institutional Ethics Committee of Nanfang Hospital and conducted in accordance with the Declaration of Helsinki (NFEC-2025-057). Written informed consent was obtained from all participants.

### Animals.

Eight-week-old C57BL/6J mice (20–24 g) were obtained from the Experimental Animal Center of Southern Medical University. TEC-specific *DDX18-*knockout mice were generated at 6 weeks by tail-vein injection of an AAV9-CRISPR/Cas9 system driven by the TEC-specific Ksp promoter. Exons 2–4 of mouse *DDX18* (NM_025860.3) were excised using sgRNA-A1 (GGAACAGCCACGGCCAAGTG, targeting the region upstream of exon 2) and sgRNA-A2 (AGTCTTCAGTATAGCCAAAC, targeting the region downstream of exon 4), inducing a frameshift mutation. Due to AAV packaging constraints, 2 vectors were used: AAV-U6>sgRNA-A1–U6>sgRNA-A2–CMV>EGFP and AAV-Ksp>SaCas9, with Ksp promoter restricting SaCas9 expression to TECs. Recombinant AAV9 particles were produced in HEK293T cells by triple transfection, purified by iodixanol-gradient ultracentrifugation, and quantified by qPCR. Each vector was administered at 2 × 10¹¹ viral genomes per mouse. Renal DDX18 deletion was confirmed by immunoblotting and immunofluorescence. All procedures were approved by the Laboratory Animal Care and Ethical Committee of Southern Medical University (D2022054).

### BIRI model.

For the BIRI AKI-to-CKD model, renal pedicles were clamped for 30 min under ketamine (100 mg/kg) and xylazine (10 mg/kg) anesthesia. Body temperature was maintained at 37°C, and incisions were closed after reperfusion. Sham-operated mice underwent identical procedures without clamping. For pharmacological treatment, the LDH inhibitor (R)-GNE-140 (Merck, HY-100742A) was administered orally at 100 mg/kg every 48 h. Sodium lactate, prepared at 200 mg/mL in PBS, was slowly injected intraperitoneally at 2 g/kg every 48 h for 14 days.

### Lactate and pH measurement.

Tissue and serum lactate were measured using a Lactate Assay Kit (Sigma-Aldrich, MAK064). Kidney tissue (50 mg) was homogenized in 200 μL assay buffer and centrifuged at 13,000*g* for 10 min at 4°C, with values normalized to tissue weight. Human plasma lactate was obtained from UK Biobank NMR data. Blood pH was measured at 37°C using a radiometer capillary electrode and PHM 73 analyzer (Radiometer Copenhagen).

### Serum biochemistry.

Mouse blood was centrifuged at 1,000*g* for 20 min at 4°C to isolate serum. Serum creatinine and BUN were measured using an automated biochemical analyzer (Mindray BS-240) according to the manufacturer’s instructions.

### Histological analysis and injury scoring.

Kidneys were fixed in 4% paraformaldehyde, paraffin embedded, sectioned at 4–5 μm, and stained with Sirius Red (Merck, 365548) to assess tissue architecture and fibrosis. Tubular injury in the corticomedullary region was scored as 0 (none), 1 (<25%), 2 (25%–50%), 3 (50%–75%), or 4 (>75%). At least 9 fields per sample were independently evaluated by 2 investigators blinded to group allocation.

### Cell culture and H/R model.

HK-2 cells (ATCC, CRL-2190) and HEK293T cells (ATCC, CRL-3216) were maintained in DMEM (Thermo Fisher Scientific, 11965092) supplemented with 10% FBS (Thermo Fischer Scientific, A5256701), 100 U/mL penicillin, and 100 mg/mL streptomycin (Merck, P4333) at 37°C in 5% CO_2_. For H/R experiments, cells were incubated in glucose- and serum-free DMEM under 1% O_2_ for 24 h, followed by reoxygenation under normoxia.

### Isolation of mouse renal tubular cells.

Kidneys were excised, minced, and digested with collagenase type IV and dispase (Roche) at 37°C for 30–45 min. The tissue was mechanically dissociated, filtered through a fine mesh, and centrifuged to remove debris. Isolated renal tubular cells were cultured in serum-supplemented DMEM/F-12.

### Cell migration assay.

HK-2 cells were seeded in 6-well plates at 1 × 10^5^ cells/well and grown to confluence. A scratch was made with a sterile 200 μL pipette tip, followed by PBS washing and serum-free DMEM replacement. Wound closure at 0 and 48 h was imaged by phase contrast microscopy and quantified using ImageJ (NIH).

### 4D label-free quantitative lactylproteomic analysis.

HK-2 cells were sonicated in 8 M urea buffer with protease inhibitors, centrifuged at 12,000*g* for 10 min at 4°C, and quantified by BCA assay. Proteins were reduced with 5 mM dithiothreitol at 56°C for 30 min, alkylated with 11 mM iodoacetamide for 15 min in darkness, diluted with 100 mM TEAB to <2 M urea, and digested with trypsin at 1:50 overnight and 1:100 for 4 h. Lactylated peptides were enriched overnight at 4°C in NETN buffer using anti–l-lactyl-lysine antibody beads (PTM Bio, PTM-1404), eluted with 0.1% trifluoroacetic acid, and desalted using C18 ZipTips (Merck Millipore). Peptides were analyzed on a nanoElute UHPLC–timsTOF Pro system (Bruker Corporation) using a 60 min, 6%–80% acetonitrile gradient at 450 nL/min. parallel accumulation-serial fragmentation (PASEF) settings included a 1.70 kV electrospray voltage, 100–1,700 *m*/*z* range, 10 MS/MS scans per cycle, and 24 s dynamic exclusion. Functional enrichment and network analyses were performed using Metascape, Cytoscape, and MCODE. Subcellular localization was assigned using WoLF PSORT. Analyses were performed by Jingjie PTM Biolabs (Hangzhou, China).

### RIP-seq analysis.

RIP-seq was performed using the BersinBio kit (Bes5101). DDX18-deficient HK-2 cells expressing DDX18^WT^ or DDX18^K116R^ were subjected to H/R. Cell lysates were incubated overnight at 4°C with an anti-DDX18 antibody or control IgG. Protein-RNA complexes were captured with A/G magnetic beads for 1 h at 4°C and washed 6 times, and RNA was extracted using TRIzol (Thermo Fisher Scientific, 15596026CN). RNA quality was assessed using an Agilent 2100 Bioanalyzer and SimpleNano spectrophotometer. Reads were quality checked with FastQC, trimmed using Trimmomatic, aligned to GRCh38/hg38 with STAR, and quantified using featureCounts with Ensembl annotations. Differential enrichment between immunoprecipitated and matched input or IgG samples was analyzed using DESeq2. Transcripts with adjusted *P* < 0.05 and |log_2_ fold change| ≥ 1 were considered significant interactors. GO and KEGG enrichment analyses were performed for functional annotation.

### RNA-seq analysis.

Total RNA was extracted with TRIzol from H/R-treated DDX18-deficient HK-2 cells expressing DDX18^WT^ or DDX18^K116R^. RNA integrity was assessed using an Agilent 2100 Bioanalyzer. Poly(A)^+^ mRNA was isolated, fragmented, and reverse transcribed. Double-stranded cDNA underwent end repair, A-tailing, adapter ligation, and selection of approximately 200 bp fragments using AMPure XP beads (Beckman, A63881). Libraries were amplified, quality controlled, and sequenced on an Illumina HiSeq platform by Novogene. Reads were assessed with FastQC, trimmed using Trimmomatic, aligned to GRCh38/hg38 with STAR, and quantified using featureCounts with Ensembl annotations. DESeq2 identified differentially expressed genes at adjusted *P* < 0.05 and |log_2_ fold change| ≥ 1. Principal component analysis and hierarchical clustering were used to assess reproducibility, followed by GO and KEGG analyses.

### RT-qPCR analysis.

Total RNA was extracted from kidney tissues or cells using TRIzol. RNA (1 μg) was reverse transcribed using TranScript All-in-One SuperMix (TransGene Biotech, AE341-02), and qPCR was performed with TransScript Green SuperMix (AQ211-01) on a 7900HT system (Applied Biosystems, Thermo Fisher Scientific). Target gene expression was normalized to β-actin using the 2^−ΔΔCt^ method.

### RNA stability analysis.

Cells were treated with actinomycin D (Merck, A4262), and RNA was collected at 0, 2, 4, 6, and 8 h. *CD44* mRNA levels were measured by RT-qPCR and expressed relative to baseline. mRNA half-life was calculated by 1-phase decay regression.

### Immunoblot analysis.

Tissues and cells were lysed in strong RIPA buffer (Beyotime, P0013B) containing protease inhibitors (Merck, P9599). After sonication and centrifugation, protein concentrations were determined by BCA assay. Equal protein amounts were separated by 10% SDS-PAGE and transferred to PVDF membranes. Membranes were blocked with 1% BSA and incubated with primary antibodies including anti-DDX18 antibody (Proteintech, 28502-1), fibronectin polyclonal antibody (Proteintech, 15613-1-AP), collagen type I monoclonal antibody (Proteintech, 66761-1-Ig, clone 3D5E8), α-SMA polyclonal antibody (Proteintech, 14395-1-AP), β-actin monoclonal antibody (Proteintech, 66009-1-Ig, clone 2D4H5), CD44 polyclonal antibody (Proteintech, 15675-1-AP), and anti–l-lactyl lysine rabbit mAb (PTM Bio, PTM-1401) overnight at 4°C. They were then incubated with HRP-conjugated secondary antibodies for 1 h at room temperature and developed by enhanced chemiluminescence.

### IP and LC-MS/MS analysis.

Cells were lysed for 20 min at 4°C in weak RIPA buffer (Beyotime, P0013D) containing protease inhibitors and clarified by centrifugation. Total protein (0.1 mg) was incubated with the indicated antibody and 100 μL protein A/G magnetic beads (MedChemExpress, HY-K0202) for 4 h at 4°C. Beads were washed 3 times, and bound proteins were collected. Liquid chromatography-tandem mass spectrometry (LC-MS/MS) was performed on a Bruker timsTOF Pro 2 mass spectrometer with a CaptiveSpray source. Data were acquired in DIA mode over 100–1,700 *m*/*z* using a 1.4 kV spray voltage, 180°C drying gas, 100 ms ramp time, and 100% duty cycle. Raw data were analyzed with DIA-NN v1.8.1 against the UniProt human proteome database.

### Immunohistochemistry and immunofluorescence.

For immunohistochemistry, kidneys were fixed in 4% paraformaldehyde, paraffin embedded, and sectioned at 3 μm. After deparaffinization, rehydration, hydrogen peroxide treatment, and citrate buffer antigen retrieval (Beyotime, P0081), sections were incubated with primary antibodies overnight at 4°C, followed by HRP-conjugated secondary antibodies and detection with a REAL EnVision system (Dako, Agilent Technologies). For tissue immunofluorescence, antigen-retrieved sections were permeabilized with 0.2% Triton X-100 for 10 min, blocked with 5% BSA for 1 h, and incubated with primary antibodies overnight at 4°C. Fluorophore-conjugated secondary antibodies were applied for 1 h, followed by DAPI staining (Merck, D9542). For cellular immunofluorescence, chamber slide cultures were fixed with 4% paraformaldehyde for 15 min, permeabilized with 0.3% Triton X-100 for 3 min, blocked, and incubated with primary antibodies overnight at 4°C and secondary antibodies (Proteintech, SA00003-2 and SA00013-3) for 1 h. Images were acquired using a Nikon AX-SHR confocal microscope under identical settings. DDX18 and nuclear structures were further examined by High Intelligent and Sensitive-Structured Illumination Microscopy (HIS-SIM) with a ×100/1.5 NA oil objective.

### Preparation of the DDX18-K116 lactylation–specific antibody.

The DDX18-K116 lactylation–specific antibody was generated in-house using New Zealand rabbits immunized with the synthetic lactylated peptide KKRK(Lac)MVNDAEPDTK. Rabbits were immunized 6 times according to the immunization schedule, and antiserum titers were monitored by indirect ELISA after the third to sixth immunizations. Serum was collected when antibody titers exceeded 1:32,000, followed by antigen-specific affinity purification. Purified antibodies were required to reach titers >1:64,000 and demonstrated selective recognition of the lactylated peptide by dot blot analysis.

### Generation of DDX18- and p300-knockout cells.

HK-2 cells were transfected with 2 μg of DDX18 or p300 CRISPR/Cas9 knockout plasmids (Santa Cruz Biotechnology, sc-406057 and sc-400055) using UltraCruz reagent (sc-395739). Protein expression was assessed by immunoblotting 24 h after transfection.

### Generation of DDX18^WT^- and DDX18^K116R^-expressing cells.

DDX18-knockout HK-2 cells were generated using the DDX18 CRISPR/Cas9 plasmid and validated by immunoblotting. Verified cells were reconstituted with DDX18^WT^ or DDX18^K116R^ plasmids using Lipofectamine 2000 (Invitrogen, 11668019), and exogenous expression was confirmed before subsequent experiments.

### Peptide synthesis.

Peptides were synthesized by solid-phase methods, purified to >98% by reverse-phase HPLC, and verified by MS. d–amino acid variants were used for in vivo studies. Lyophilized peptides were dissolved in sterile PBS at 10 mM, filter sterilized, aliquoted, stored at −80°C, and rechecked by analytical HPLC before use. The inhibitory peptide sequences were as follows: Peptide-1#, HLYVSPWGGKKKRKMANDAGPD; Peptide-2#, HLYVSPWGGKKKKKKKRKMAND; Peptide-3#, HLYVSPWGGKKKKKRKMANDAG; K116-Peptide-4#, HLYVSPWGGEVKKKKKKKRKMA; and Peptide-5#, HLYVSPWGGKRKMANDAGPDTK.

### Synthesis and biodistribution of PLGA-Ser-MOF-K116-pe.

PLGA-Ser-MOF-K116-pe nanocomposites were synthesized in 3 steps. PLGA and serine separately dissolved in DMF were conjugated after serine activation with equimolar EDC·HCl/DMAP for 30 min. After 8 h, PLGA-Ser was water precipitated, washed 5 times, and lyophilized. Zirconium-based MOFs were sonicated in anhydrous dimethylacetamide and activated with 10 mM EDC·HCl and 5 mM NHS for 30 min, then reacted overnight under nitrogen with K116-targeting peptide at a 1:10 MOF/peptide molar ratio. The resulting peptide-loaded MOFs were washed and lyophilized. PLGA-Ser and peptide-loaded MOFs (50 mg each) were probe sonicated in 10 mL DMF and stirred for 24 h for electrostatic assembly, then sequentially washed with DMF, ethanol, and water before lyophilization. For biodistribution analysis, mice received intravenous mCherry-labeled PLGA-Ser-MOF-K116-pe (10 mg/kg) and were euthanized 12 h later. Organ fluorescence was quantified using an In-Vivo FX Pro system (Bruker Corporation). Renal targeting was further evaluated by ICP-MS measurement of zirconium, tubular immunofluorescence, and electron microscopy.

### Statistics.

Statistical analyses were performed using GraphPad Prism 9.0. Data are presented as mean ± SEM. Unpaired 2-tailed Student’s *t* tests were used for 2-group comparisons, and 2-way ANOVA was used for multiple-group comparisons. Pearson’s correlation was used to assess associations between variables. *P* < 0.05 was considered statistically significant.

### Study approval.

All animal procedures were approved by the Laboratory Animal Care and Ethical Committee of Southern Medical University and complied with all relevant ethical regulations (no. D2022054). Ethical approval was granted by the North West Research Ethics Committee (06/MRE08/65). The study was approved by the Institutional Ethics Committee of Nanfang Hospital (NFEC-2025-057) and conducted in accordance with the Declaration of Helsinki. Written informed consent was obtained from all participants.

### Data availability.

Raw RNA-seq and RIP-seq data have been deposited in the NCBI GEO and are publicly available under accession numbers GSE333635 and GSE333824, respectively. The raw data from the 4D label-free quantitative lactylproteomic, IP, and LC-MS/MS analysis have been deposited in the ProteomeXchange Consortium under accession numbers PXD078736 and PXD079002, respectively. Values for all data points in graphs are reported in the Supporting Data Values file. All other data supporting the findings of this study are available upon request from the corresponding authors.

## Author contributions

LD, JX, and MT contributed equally to this work and performed formal analysis of all data. DZ, TT, JA, HH, and LD conceptualized and planned all experiments, methodology, and analysis. Funding acquisition was done by DZ and LD acquired funding and drafted the original manuscript. DZ, LD, JX, MT, XZ, and J Zhao reviewed and edited the manuscript. SL, YL, TW, and J Zhou performed sequencing experiments. LD, JX, MT, and SL performed in vivo experiments. LD, JX, MT, YL, and QC performed in vitro experiments. JG and JA obtained human samples. DZ provided project management and oversight.

## Conflict of interest

The authors have declared that no conflict of interest exists.

## Funding support

- National Natural Science Foundation of China (82471852 and 82271782 to DZ).
- National Natural Science Foundation of China (82370744 to JA).
- National Natural Science Foundation of China (82402129 to LD).
- Science and Technology Planning Project of Guangdong Province (2024A0505040013 to DZ).
- Guangdong Provincial Key Laboratory of Immune Regulation and Immunotherapy (2022B1212010009 to DZ).

## Supplementary Material

Supplemental data

Unedited blot and gel images

Supplemental table 3

Supporting data values

## Figures and Tables

**Figure 1:**
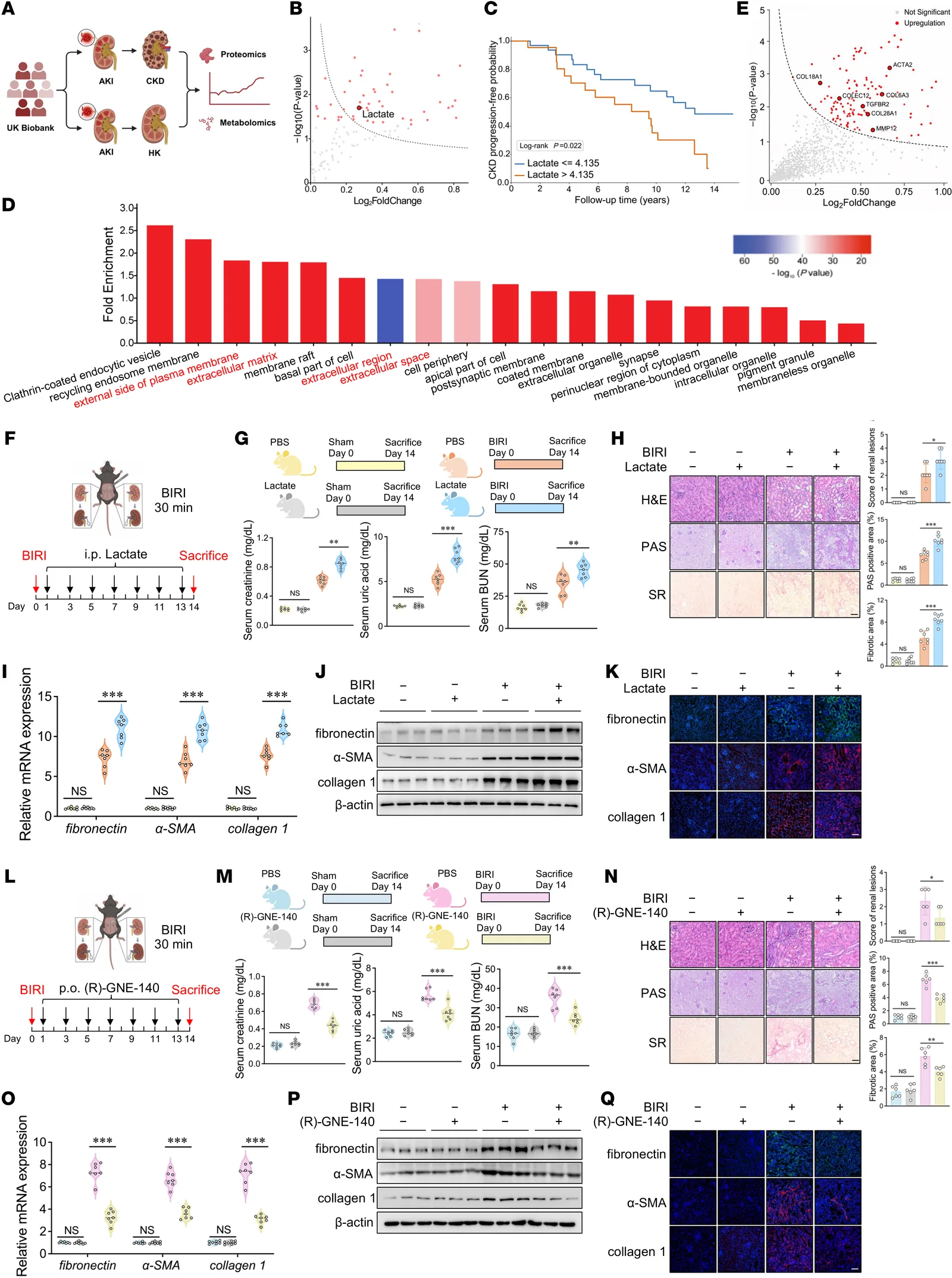
Elevated lactate levels accelerate the progression from AKI to CKD. (**A**–**E**) UK Biobank cohort analysis. (**A**) Study workflow for a nested case-control analysis within the UK Biobank. Cases comprised baseline AKI patients who progressed to CKD, whereas nonprogressing AKI patients served as controls. Baseline proteomic and metabolomic profiles were analyzed. (**B**) Upregulated metabolites in AKI patients progressing to CKD, with a marked increase in lactate. (**C**) Kaplan-Meier curve of cumulative CKD incidence among baseline AKI patients stratified by high (>4.135 mmol/L) and low (≤4.135 mmol/L) circulating lactate levels. (**D**) Gene Ontology (GO) enrichment of upregulated proteins in patients who progressed to CKD, highlighting ECM remodeling pathways. (**E**) Volcano plot illustrating proteomic alterations between AKI patients with and without CKD progression. (**F**–**K**) Lactate exacerbates renal fibrosis in a murine BIRI model. (**F**) Experimental design for lactate administration (2 g/kg, i.p.) in BIRI mice. (**G**) Serum creatinine, uric acid, and BUN were assessed on day 14 after injury (*n* = 7 per group). (**H**) Representative H&E, periodic acid–Schiff (PAS), and Sirius Red (SR) staining and quantitative scoring showing enhanced tubular injury and collagen deposition. Scale bar: 100 μm. (**I**–**K**)Fibrotic markers (fibronectin, α-SMA, and collagen type I) analyzed by qPCR (**I**), Western blotting (**J**), and immunofluorescence (**K**). Scale bar: 100 μm. (**L**–**Q**) LDHA inhibition attenuates AKI-to-CKD progression. (**L**) Timeline for (R)-GNE-140 (100 mg/kg) treatment. (**M**) Improved renal function parameters in the inhibitor-treated group (*n* = 7 per group). (**N**) Representative histopathology (H&E, PAS, and Sirius Red) and quantitative scoring showing reduced tubular injury and collagen deposition. Histopathological amelioration of fibrosis features. Scale bar: 100 μm. Decreased mRNA (**O**), protein (**P**), and histological levels (**Q**) of fibrotic markers following LDHA inhibition. Representative data from 1 of 3 independent experiments are shown. Data in **G**–**I** and **M**–**O** were analyzed by 2-way ANOVA. *P < 0.05, **P < 0.01, ***P < 0.001.

**Figure 2:**
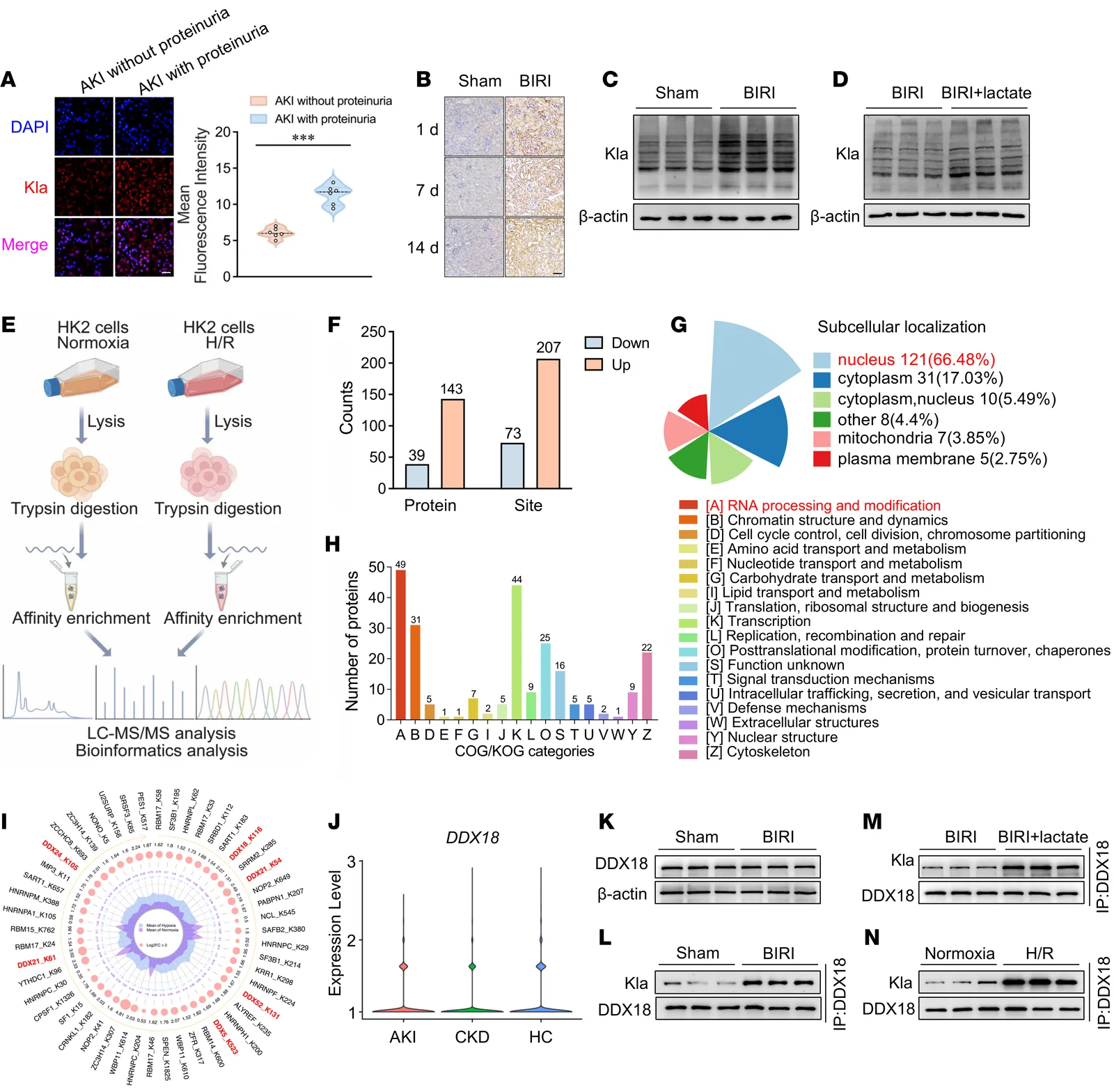
Protein lactylation dynamics in kidney injury and TECs under hypoxic conditions. (**A**) Renal immunofluorescence showing increased global lysine lactylation (pan-Kla) in AKI patients with versus without proteinuria. Scale bar: 100 μm. (**B**–**D**) Temporal changes in murine BIRI. (**B**) Pan-Kla immunohistochemistry showing progressive lactylation in renal TECs after injury. (**C**) Immunoblotting confirming sustained renal lactylation on day 14. (**D**) Lactate supplementation (2 g/kg, i.p.) further increased renal lactylation. (**E**–**I**) Lactylomic profiling of HK-2 cells under H/R. (**E**) Experimental workflow (*n* = 3). (**F**) Quantitative analysis identified 280 lactylation sites across 182 differentially lactylated proteins. (**G**) Subcellular distribution of these proteins. (**H**) Clusters of Orthologous Groups (COG) and Eukaryotic Orthologous Groups (KOG) analysis showing enrichment in RNA-processing pathways. (**I**) Radar plot of proteins enriched in RNA processing and modification; blue and purple values indicate relative abundance under normoxia and H/R, and black values indicate log_2_ fold change. (**J** and **K**) DDX18 expression remained unchanged during renal injury. (**J**) Single-nucleus RNA-seq data (GEO: GSE183277) showing comparable *DDX18* expression in AKI, CKD, and healthy controls (HC). (**K**) Renal DDX18 protein levels in BIRI mice. (**L**–**N**) BIRI increases DDX18 lactylation. (**L**) Renal DDX18-Kla was elevated after BIRI. (**M**) Lactate supplementation further enhanced DDX18 lactylation. (**N**) H/R induced DDX18 lactylation in primary TECs without altering total DDX18 protein. Representative data from 1 of 3 independent experiments are shown. Data in **A** were analyzed using an unpaired 2-tailed Student’s *t* test. ***P < 0.001.

**Figure 3:**
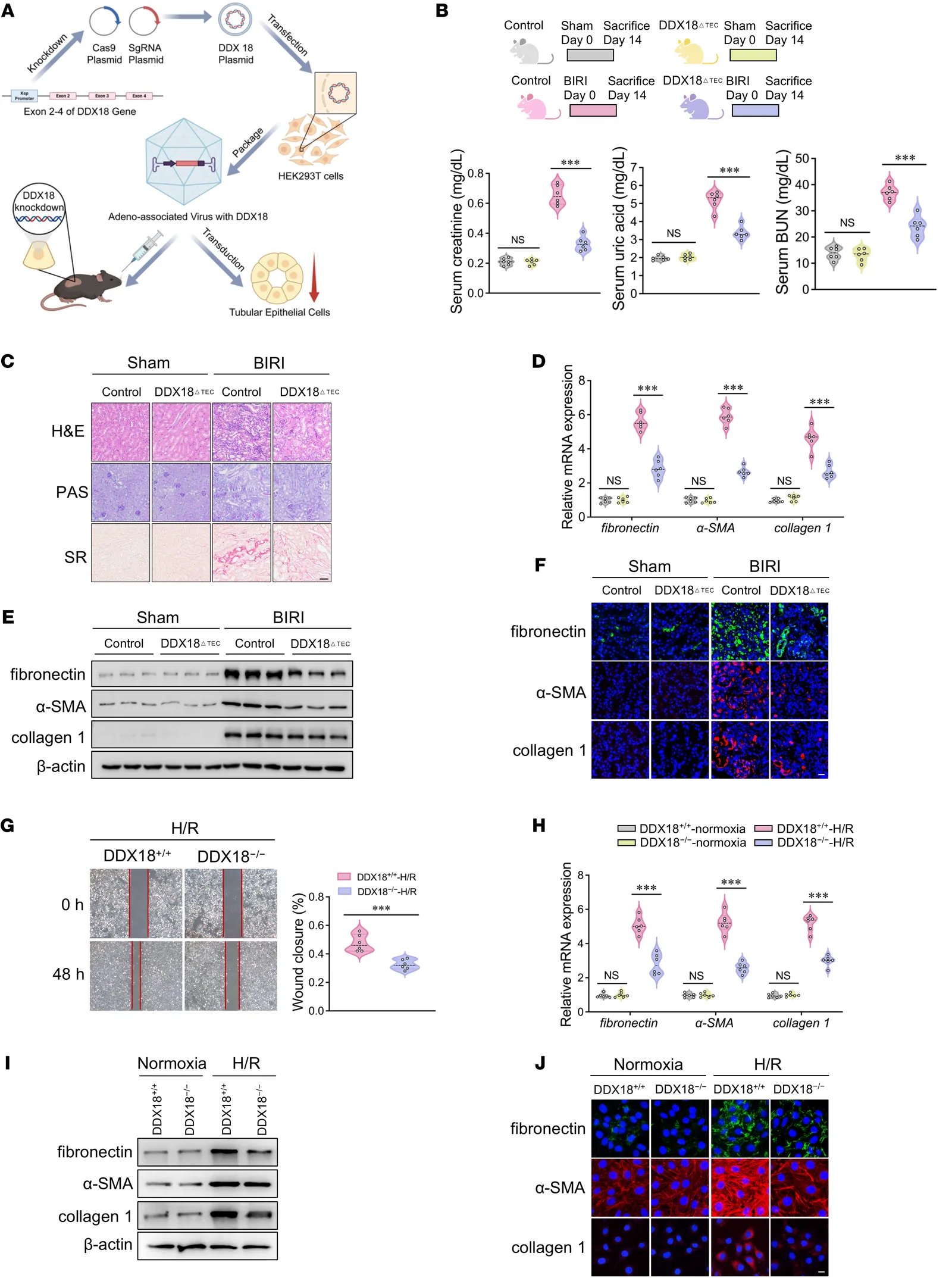
TEC-specific ablation of *DDX18* mitigates AKI-to-CKD progression in BIRI mice. (**A**) Schematic of TEC-specific *DDX18* knockout using a Ksp promoter–driven AAV9 vector. (**B**–**F**) Control and TEC-specific *DDX18*-knockout mice underwent BIRI, and kidneys were collected on day 14. (**B**) Improved renal function, indicated by reduced serum creatinine, uric acid, and BUN. (**C**) Representative H&E, PAS, and Sirius Red staining. Scale bar: 100 μm. Downregulation of fibrotic markers at mRNA (**D**), protein (**E**), and histological (**F**) expression levels in TEC-specific *DDX18*-knockout BIRI mice. Scale bar: 100 μm. (**G**–**J**) In vitro validation using *DDX18*-deficient TECs under H/R conditions. (**G**) Scratch wound assay showing impaired migration of *DDX18*^−/−^ versus *DDX18*^+/+^ HK-2 cells under H/R, with wound closure (%) quantified at 48 h after scratch. (**H**–**J**) Decreased fibrotic marker expression by RT-qPCR (**H**), Western blotting (**I**), and immunofluorescence (**J**). Scale bar: 20 μm. Representative data from 1 of 3 independent experiments are shown. Data in **B**, **D**, and **H** were analyzed using 2-way ANOVA and in **G** using an unpaired 2-tailed Student’s *t* test. ***P < 0.001.

**Figure 4:**
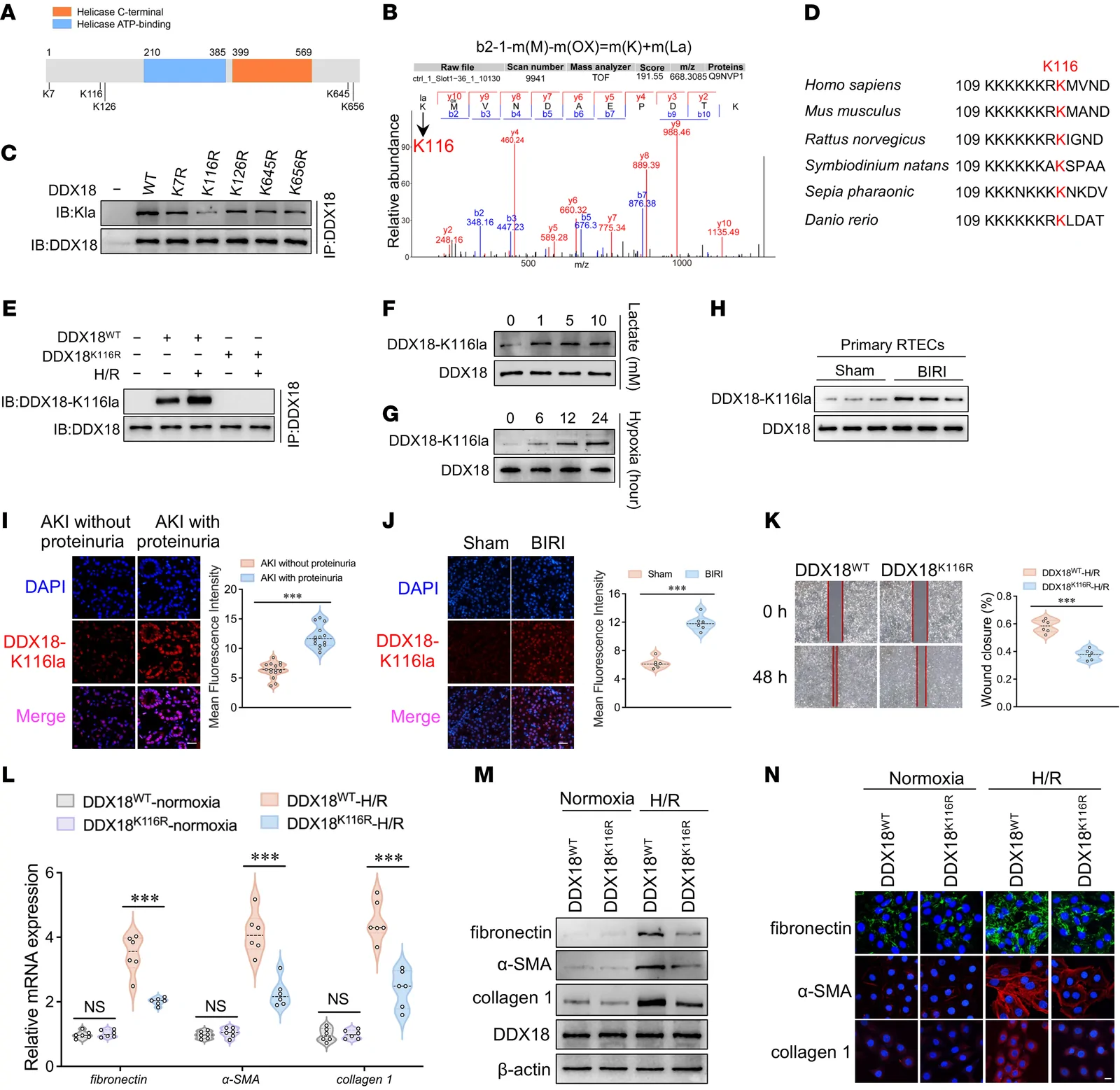
DDX18-K116 lactylation drives the AKI-to-CKD transition in BIRI mice. (**A**) Domain architecture of DDX18 highlighting putative lactylation sites. (**B**) MS analysis confirming lactylation at K116. The *x* axis represents the mass‑to‑charge ratio (*m*/*z*), corresponding to the molecular mass divided by the charge state of the peptide. The *y* axis represents the intensity of the fragment ion peaks. Red peaks correspond to b‑ions and blue peaks to y‑ions. For sequencing, b‑ions are counted from the N‑terminus, while y‑ions are counted from the C‑terminus. The calculation formula is b2-1-m(M) – m(OX) = m(K) + m(La). (**C**) DDX18 lactylation in HK-2 cells expressing DDX18^WT^ or multiple K→R mutants of DDX18. (**D**) Cross-species alignment showing conserved K116 (red) across 7 species. (**E**) DDX18-K116 lactylation in HK-2 cells expressing DDX18^WT^ or the DDX18^K116R^ mutant under H/R stress. (**F**) DDX18-K116 lactylation in HK-2 cells treated with lactate. (**G**) Hypoxia treatment induced lactylation of DDX18-K116. (**H**) DDX18 K116 lactylation was significantly increased in primary renal TECs from BIRI mice. (**I** and **J**) Spatial distribution of DDX18-K116 lactylation in vivo. (**I**) Increased DDX18-K116 lactylation in renal tissues from AKI patients with proteinuria versus without proteinuria. Scale bar: 100 μm. (**J**) Immunofluorescence staining of DDX18-K116 lactylation in kidney sections from BIRI mice after 24 h of reperfusion. Scale bar: 100 μm. (**K**–**N**) In vitro validation using HK-2 cells expressing DDX18^K116R^ under H/R stress. (**K**) Wound healing assay showing the migration capacity of DDX18^WT^- or DDX18^K116R^-expressing HK-2 cells, with wound closure (%) quantified at 0 and 48 h. Fibrotic marker expression (**L**) was determined by RT-qPCR analysis. The levels of fibrosis-related proteins were determined by Western blotting (**M**) and immunofluorescence (**N**). Scale bar: 20 μm. Representative data from 1 of 3 independent experiments are shown. Data in **I**–**K** were analyzed using an unpaired 2-tailed Student’s *t* test and in **L** using 2-way ANOVA. ***P < 0.001.

**Figure 5:**
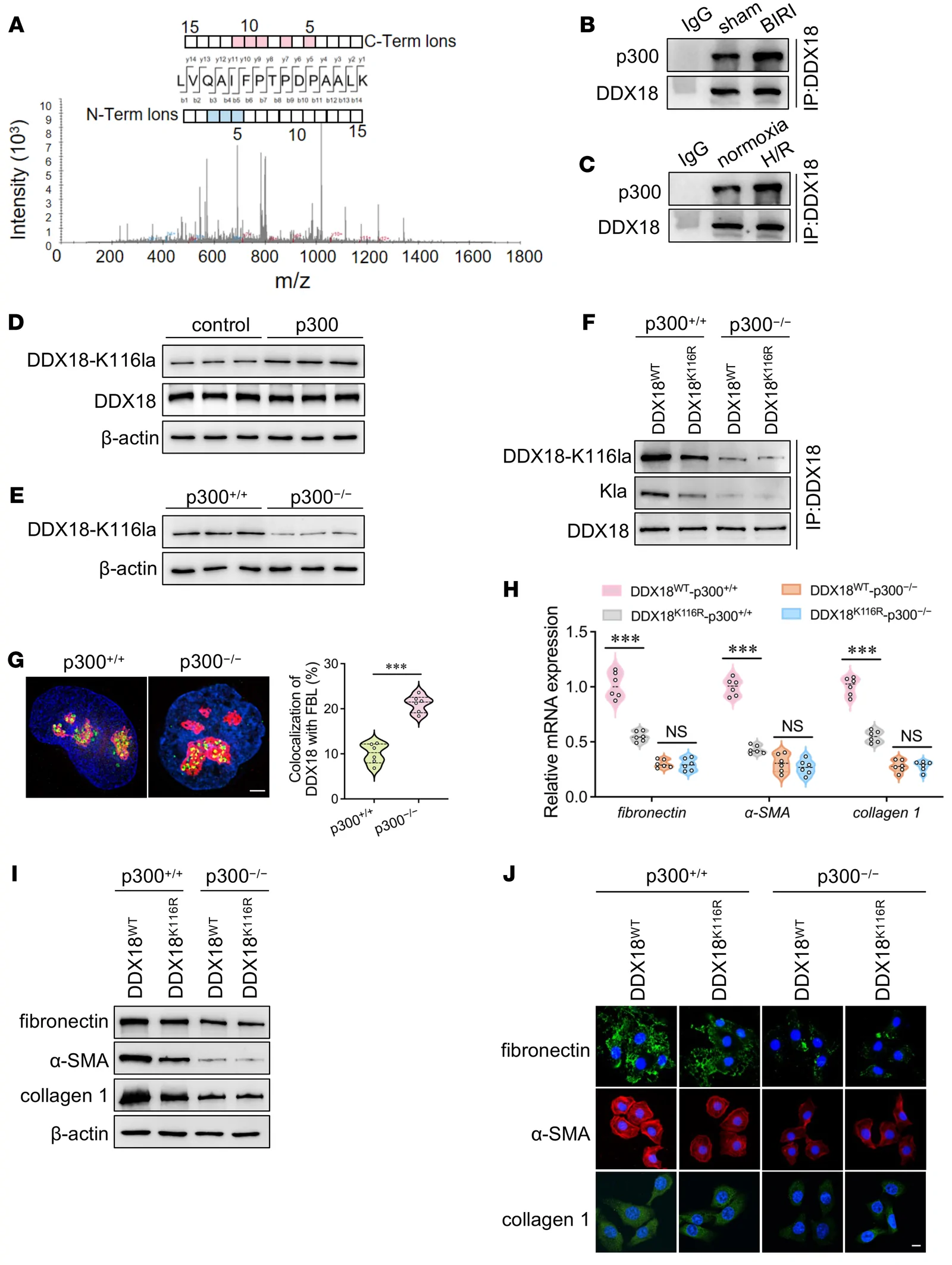
p300 mediates hypoxia-induced DDX18 lactylation, which drives fibrotic responses. (**A**–**C**) p300 interacts with DDX18 under hypoxia. (**A**) Proteomic interactome analysis of DDX18 immunoprecipitates from HK-2 cells with MS confirmed p300 association. (**B**) Co-IP validating the endogenous DDX18-p300 interaction in kidney tissues from sham and BIRI mice. (**C**) Co-IP confirming the DDX18–p300 interaction in HK-2 cells under H/R conditions. (**D**–**F**) Regulation of DDX18 lactylation by p300. (**D**) Increased K116-specific lactylation of DDX18 following p300 overexpression in H/R-treated cells. (**E**) Reduced K116-specific DDX18 lactylation in CRISPR/Cas9-mediated p300 knockout cells under H/R. (**F**) Immunoblot analysis of DDX18 lactylation in p300^+/+^ or p300^−/−^ HK-2 cells expressing DDX18^WT^ or DDX18^K116R^ under H/R conditions. (**G**) Immunofluorescence showing the subcellular localization of DDX18 (red) relative to the nucleolar marker fibrillarin (FBL; green) in WT and p300 knockout cells under H/R conditions. Scale bar: 5 μm. (**H**–**J**) Fibrotic marker expression was determined by RT-qPCR (**H**), Western blotting (**I**), and immunofluorescence (**J**). Scale bar: 20 μm. Representative data from 1 of 3 independent experiments are shown. Data in **G** were analyzed using an unpaired 2-tailed Student’s *t* test and in **H** using 2-way ANOVA. ***P < 0.001.

**Figure 6:**
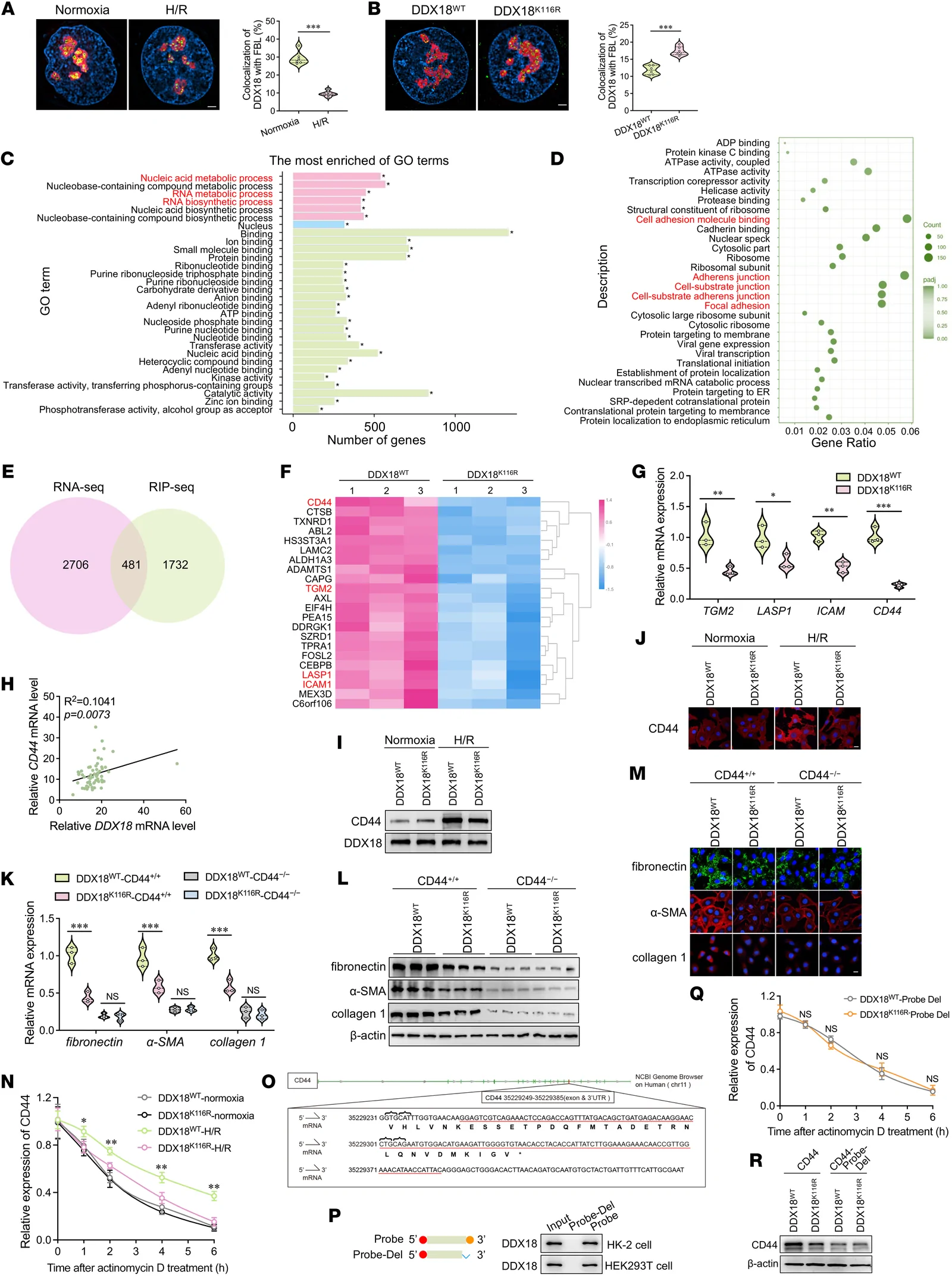
DDX18-K116 lactylation facilitates TEC activation through *CD44* RNA binding. (**A**) Immunofluorescence showing the subcellular localization of DDX18 (red) relative to the nucleolar marker fibrillarin (FBL; green) in HK-2 cells under normoxia or H/R conditions. Scale bars: 5 μm. (**B**) Subcellular localization of DDX18 (red) with nucleolar (green) in HK-2 cells expressing the DDX18^WT^ or DDX18^K116R^ mutant under H/R. Scale bars: 5 μm. (**C**) GO analysis (RIP-seq, base mean ≥ 500, IP/input ≥ 2, *P* < 0.05) of DDX18-bound transcripts in H/R-treated cells. (**D**) GO analysis (RNA-seq, fold change > 2, *P* < 0.05) of genes differentially expressed between DDX18^WT^- and DDX18^K116R^-expressing HK-2 cells under H/R. (**E**–**G**) Integrative multiomics identifies *CD44* as a key target. (**E**) Venn diagram of overlapping genes from RNA-seq and RIP-seq. (**F**) Heatmap analysis of overlapping downregulated genes in DDX18^K116R^ mutant cells, with cell adhesion–associated genes highlighted (adjusted *P* < 0.0001, |log_2_ fold change|> 0.2). (**G**) DDX18-deficient HK-2 cells were reconstituted with either DDX18^WT^ or DDX18^K116R^ before exposure to H/R conditions. Relative mRNA levels of candidate genes associated with adhesion function were quantified via RT-qPCR. (**H**) Transcriptomic data (GEO: GSE175759) identified the correlation between *CD44* and *DDX18* expression levels in microdissected tubulointerstitial tissue from patients with kidney disease. (**I** and **J**) The protein level of CD44 was determined by Western blotting (**I**) and immunofluorescence (**J**). Scale bar: 20 μm. (**K**–**M**) CD44^+/+^ and DDX18^−/−^ HK-2 cells were transfected with DDX18^WT^ or DDX18^K116R^ mutant and subsequently subjected to H/R. Fibrotic gene expression (**K**) was determined by RT-qPCR. The levels of fibrosis-related proteins were determined by Western blotting (**L**) and immunofluorescence (**M**). Scale bar: 20 μm. (**N**) *CD44* mRNA stability in DDX18^WT^- or DDX18^K116R^-expressing HK-2 cells under hypoxic conditions was detected by actinomycin D assay. (**O**) Schematics showing the DDX18-binding site on the *CD44* transcript by RIP-seq. (**P**) Biotinylated RNA probe and its deletion mutant (probe-Del, 35,229,249–35,229,385 nt deficiency) were incubated with HK-2 or HEK293T cell lysates and pulled down with streptavidin beads, and associated proteins were analyzed by Western blotting. (**Q** and **R**) CD44 and DDX18 double-knockout HK-2 cells were cotransfected with DDX18^WT^ or DDX18^K116R^ and a CD44-Del plasmid (CD44 region 35,229,249–35,229,385 nt deletion) or CD44 plasmid. (**Q**) *CD44* mRNA stability in HK-2 cells was detected by actinomycin D assay. (**R**) CD44 protein level was determined by Western blot analysis. Representative data from one of three independent experiments are shown. Data in **A**, **B**, **G**, and **Q** were analyzed using an unpaired 2-tailed Student’s *t* test, in **K** and **N** using 2-way ANOVA, and in **H** using Pearson’s correlation analysis. *P < 0.05, **P < 0.01,***P < 0.001.

**Figure 7:**
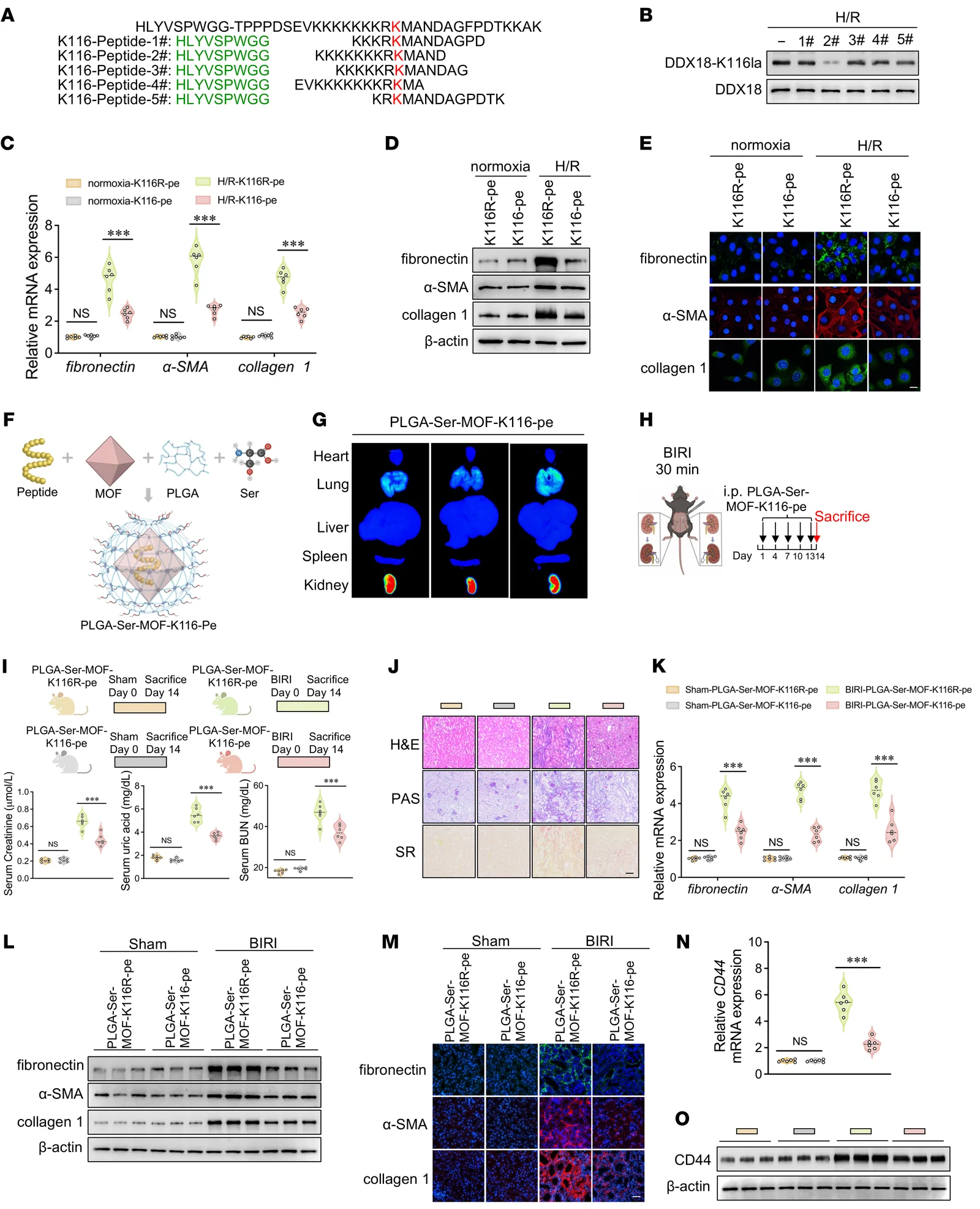
K116-targeted peptide inhibits DDX18 lactylation and attenuates AKI-to-CKD progression. (**A**–**D**) Development and in vitro validation of DDX18-K116–targeting peptides. (**A**) Structural schematic of synthetic peptides designed to competitively inhibit DDX18-K116 lactylation. (**B**) Western blot validation of peptide specificity: 5 candidate peptides were screened for their ability to inhibit DDX18-K116 lactylation in HK2 cells under H/R. (**C**–**E**) In vitro validation using HK-2 cells under H/R stress with K116-pe. (**C**) Suppression of fibrotic gene expression was determined by RT-qPCR. A concordant reduction in fibrosis-related proteins was demonstrated by Western blot (**D**) and immunofluorescence (**E**). Scale bar: 20 μm. (**F** and **G**) Engineering and biodistribution of PLGA-Ser-MOF-K116-pe nanoparticles. (**F**) Schematic of PLGA-Ser-MOF-K116-pe nanoparticle synthesis. (**G**) Ex vivo fluorescence imaging of mCherry-labeled PLGA-Ser-MOF-K116-pe nanoparticles in major organs 12 h after injection. (**H**–**O**) Therapeutic efficacy of PLGA-Ser-MOF-K116-pe in murine AKI-to-CKD progression. (**H**) Treatment schedule for targeted delivery to injured renal tubules in BIRI mice. (**I**) Improved renal function in PLGA-Ser-MOF-K116-pe–treated BIRI mice, shown by reduced serum creatinine, uric acid, and BUN levels at experiment end. (**J**) Histopathological assessment using H&E, PAS, and Sirius Red staining. Scale bar: 100 μm. Downregulation of fibrotic markers across mRNA (**K**), protein (**L**), and histological (**M**) expression levels in PLGA-Ser-MOF-K116-pe–treated BIRI mice. Scale bars: 100 μm. Reduced CD44 expression in kidney tissues from PLGA-Ser-MOF-K116-pe–treated BIRI mice was detected at both the mRNA (**N**) and protein (**O**) levels. Representative data from 1 of 3 independent experiments are shown. Data in **C**, **I**, **K**, and **N** were analyzed using 2-way ANOVA. ***P < 0.001.

## Version 1 08/13/2026

In-Press Preview

## Version 2 10/01/2026

Electronic publication
