# Supplementary material for: Lactylation-dependent DDX18 nucleolar escape regulates CD44 mRNA stability to drive fibrotic progression in kidney injury
Source: J Clin Invest. 2026 Aug 13;136(19):e201848. doi: 10.1172/JCI201848 (PMC13626828; doi:10.1172/JCI201848)
Supplement: Supplementary file 1 [file jci-136-201848-s330.pdf]

Supplementary Materials for

# **Lactylation-dependent DDX18 nucleolar escape regulates *CD44* mRNA stability to drive fibrotic progression in kidney injury**

## **The PDF file includes**

Figs. S1 to S15
Table S1 and S2

## **Other Supplementary Materials for this manuscript includes the following:**

Table S3

**Supplementary Figure 1**

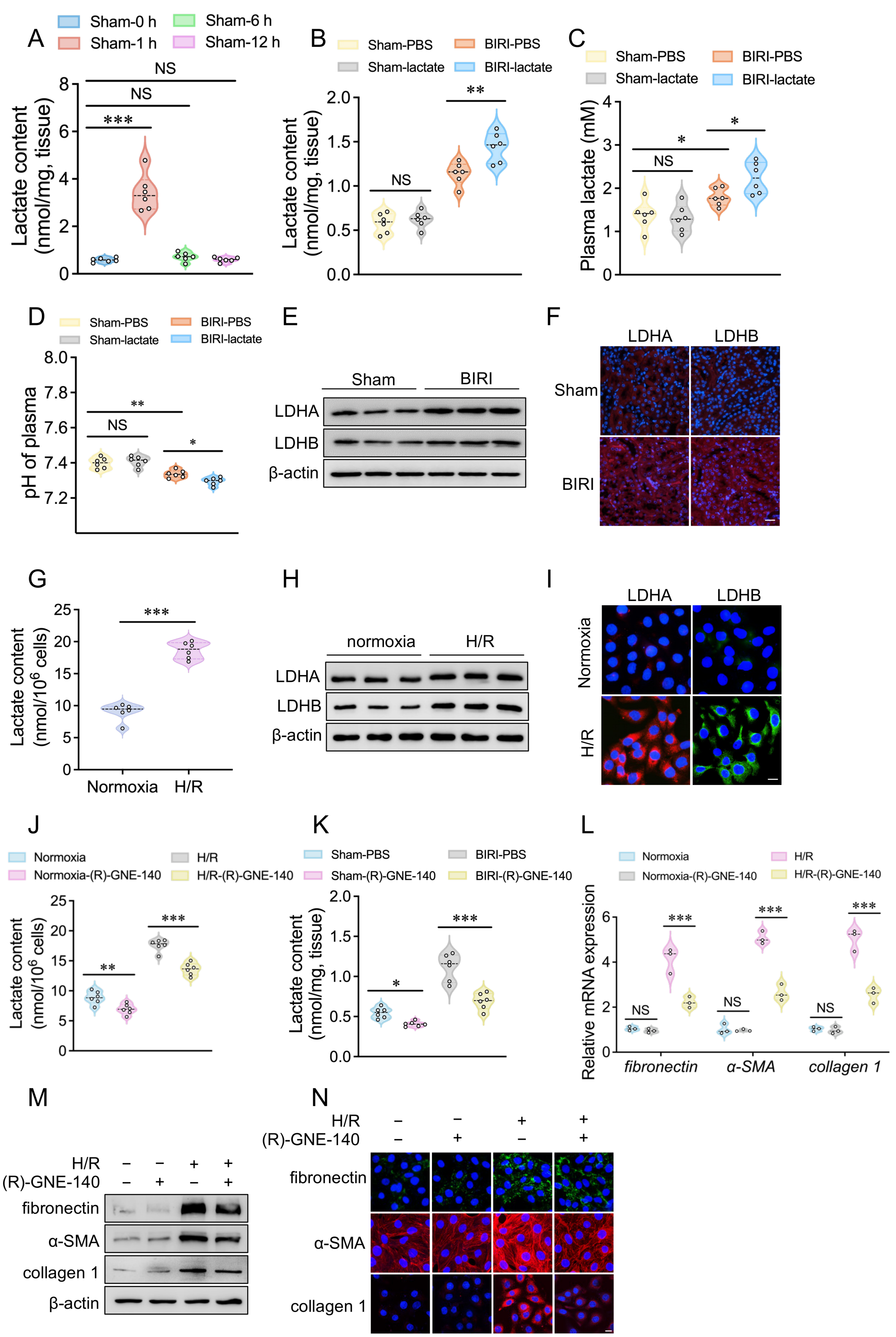

**Figure S1. Lactate is upregulated in renal tissues after BIRI and in TECs exposed to hypoxia stress**

**A,** Sham-operated mice received lactate (2 g/kg, i.p.), and renal lactate levels were measured in mice without lactate treatment and at 1, 6, and 12 h after lactate administration. **B-D,** Mice underwent 30 min of renal ischemia, and starting on day 1 of reperfusion, lactate (2 g/kg, i.p.) was administered every other day. Mice were sacrificed on day 14 post-surgery, and renal lactate levels were measured **(B)**. Plasma lactate **(C)** and pH **(D)** levels of the mice were measured on day 14. **E, F,** LDHA and LDHB expression in kidney tissues 14 days post-BIRI or sham surgery was assessed by western blotting **(E)** and immunofluorescence **(F)**. Scale bar=100 μm. **G-I**, HK-2 cells were subjected to 24 hours of hypoxia followed by 24 hours of reoxygenation. The lactate level in the culture supernatant was determined with a lactate assay kit **(G)**. The protein expression of LDHA and LDHB in HK-2 cells was assessed by western blot **(H)** and immunofluorescence analyses **(I)**. Scale bar=20 μm. **J,** Mice were subjected to sham or BIRI treatment and administered (R)-GNE-140 (100 mg/kg) via oral gavage every other day. Kidney lactate levels were assessed after 14 days. **K,** Lactate levels in HK-2 cells treated with the selective LDHA inhibitor (R)-GNE-140 (2 μM) under H/R conditions. **L-N**, Pharmacological LDHA inhibition attenuates fibrotic responses in HK-2 cells under H/R conditions. HK-2 cells were treated with the selective LDHA inhibitor (R)-GNE-140 during H/R, and fibrotic markers were assessed by quantitative RT-qPCR **(L)**, western blotting **(M)**, and immunofluorescence **(N)**. Scale bar=20 μm. Representative data from one of three independent experiments are shown. One-way ANOVA used for statistical analysis in **A**, two-way ANOVA was used for statistical analysis in **B**, **C**, **D**, **J**, **K**, **L**. Unpaired t-test was used for statistical analysis in **G**. *p<0.05, **p<0.01, ***p<0.001. NS, not significant.

**Supplementary Figure 2**

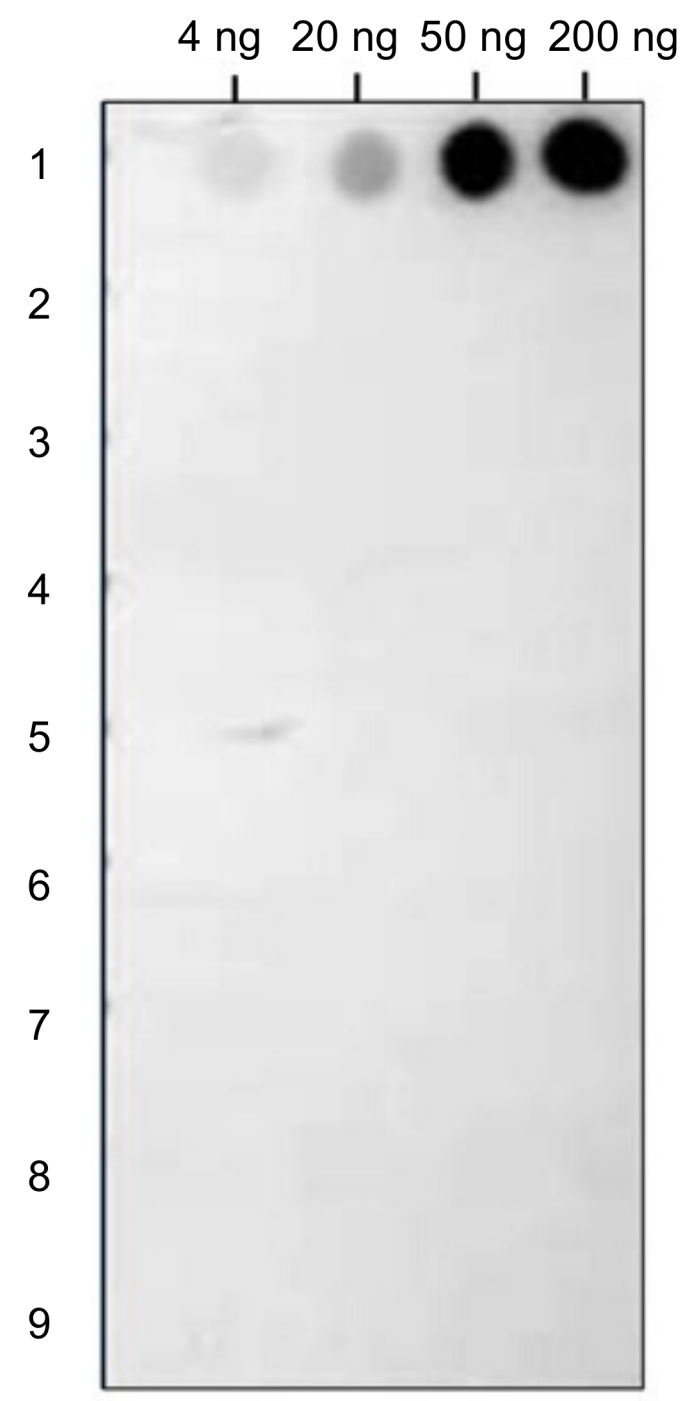


**Figure S2. Dot blot analysis demonstrating Kla antibody specificity**

1. L-lactyl lysine peptide library, 2. D-lactyl lysine peptide library, 3. Carboxyethyl lysine peptide library, 4. Acetyl lysine peptide library, 5. Butyryl lysine peptide library, 6. Crotonyl lysine peptide library, 7. 2-hydroisobutyryl lysine peptide library, 8. Succinyl lysine peptide library, 9. Control lysine peptide library.

## Supplementary Figure 3

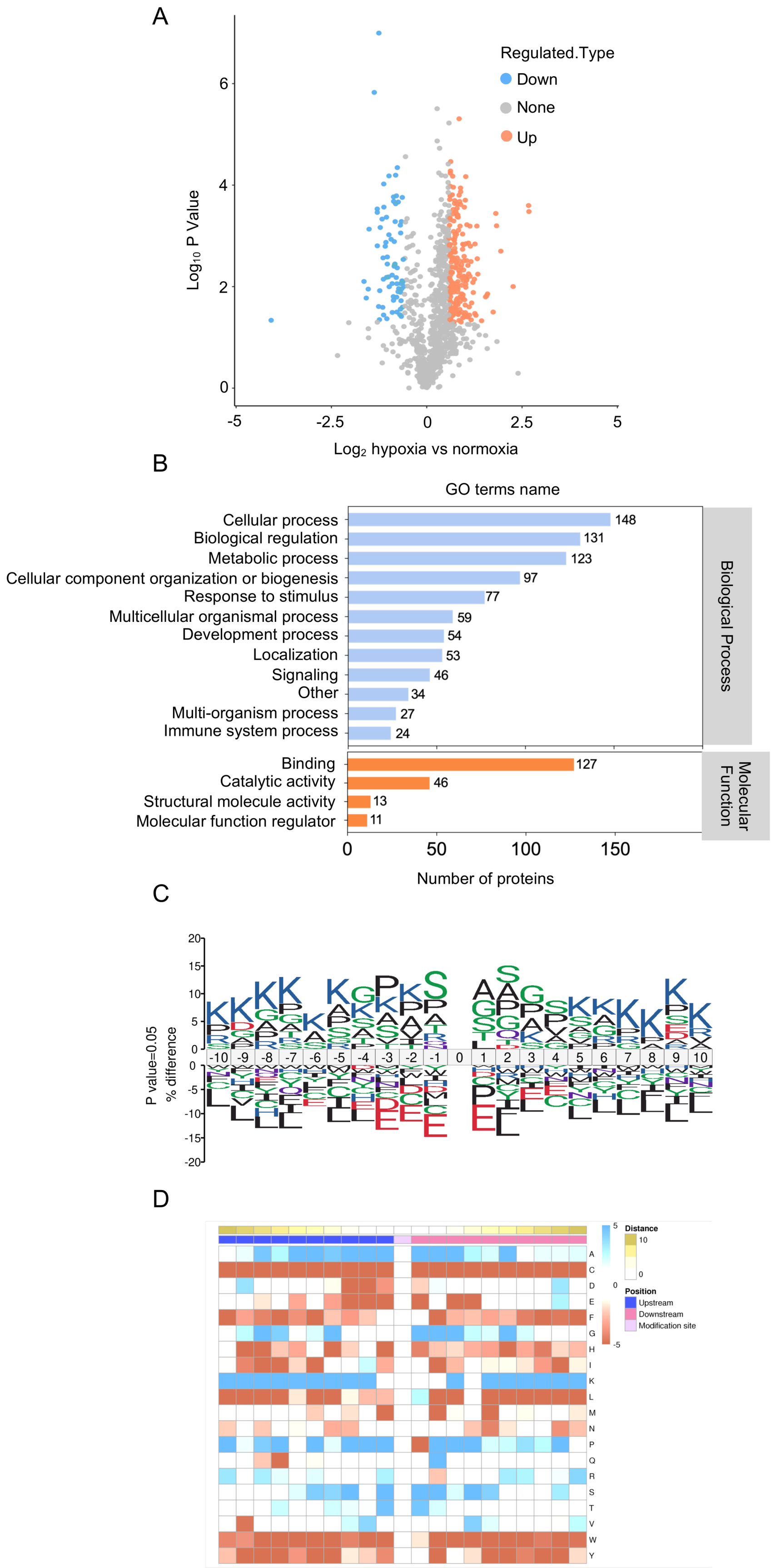

**Figure S3. Global profiling of lysine lactylation in hypoxia-exposed TECs**

**A,** Scatter plot analysis showing the quantitative distribution of identified lysine lactylation (Kla) sites plotted against peptide intensity in HK-2 cells under normoxia versus H/R conditions. **B,** GO biological process enrichment of proteins with significantly upregulated lactylation sites (FDR <0.05) following hypoxic stress. **C,** Icelogo plot demonstrating the flanking sequence preferences for identified Kla sites. **D,** Motif analysis of all identified Kla proteins. Heatmap of the amino acid compositions of the Kla sites demonstrating the frequency of certain amino acids around the modified lysine. Blue indicates a high frequency, and red indicates a low frequency.

**Supplementary Figure 4**

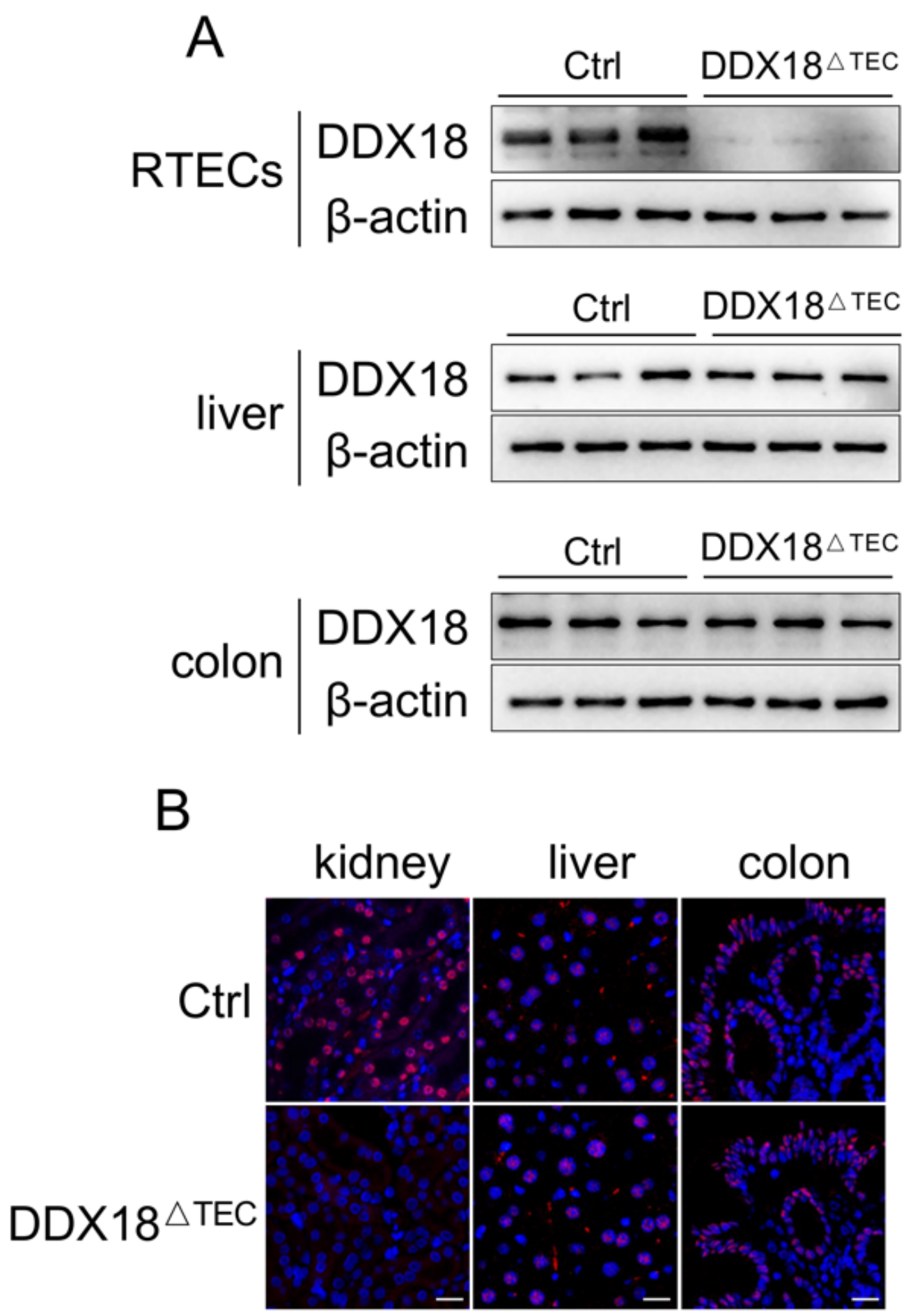


**Figure S4. Validation of TEC-specific DDX18 knockout in the mouse kidney**

TEC-specific DDX18 knockout was achieved using an AAV9 vector dirven by the Ksp promoter. DDX18 expression levels in the primary renal tubular epithelial cells, liver, and colon tissues of mice were evaluated by immunoblotting **(A)** and immunofluorescence **(B)**. Scale bar=20μm. Representative data from one of three independent experiments are shown.

Supplementary Figure 5

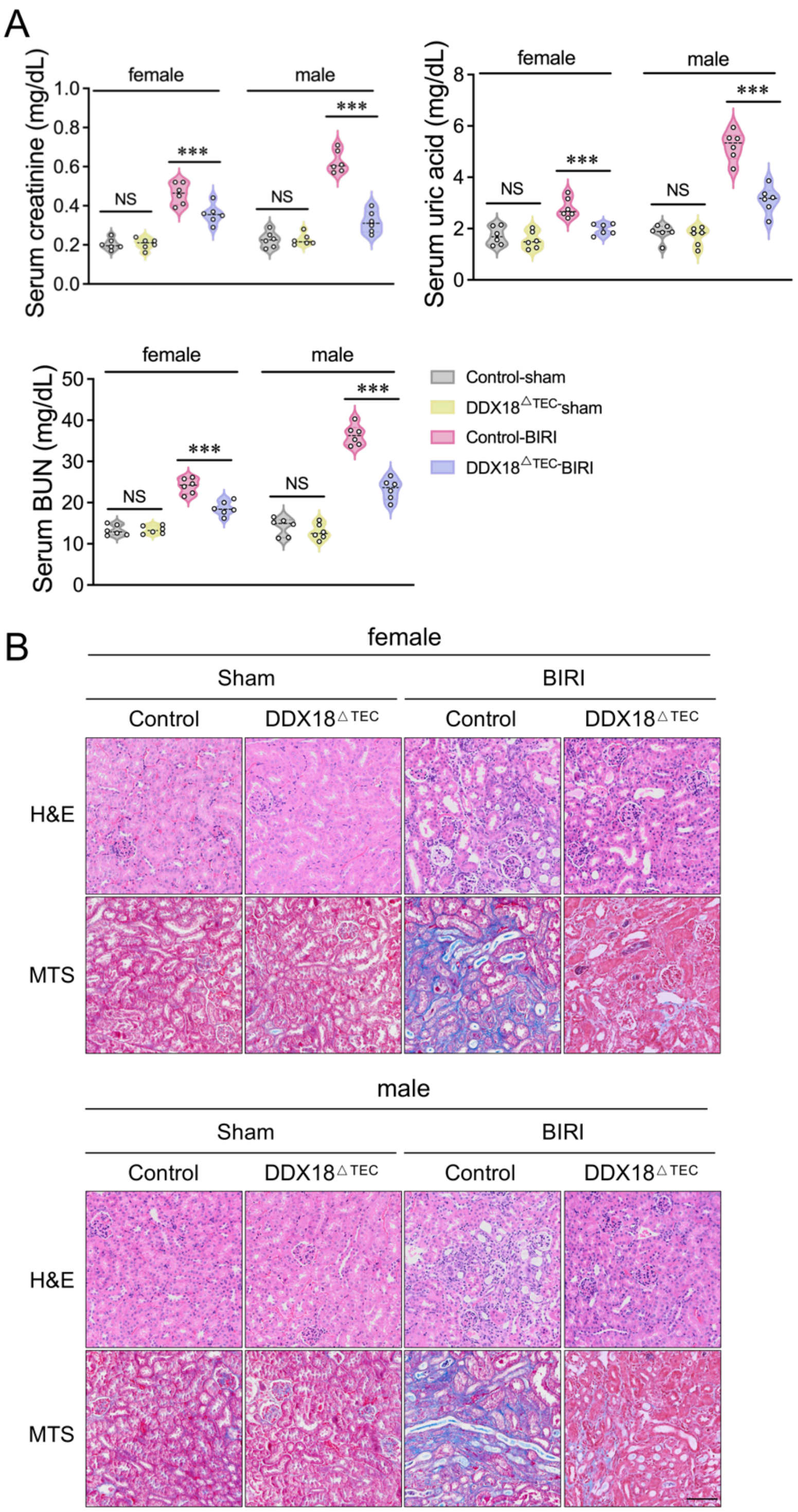


**Figure S5. TEC-specific ablation of DDX18 mitigates kidney dysfunction in BIRI female mice**

Control and DDX18$^{\Delta TEC}$ female mice underwent BIRI to induce the AKI-to-CKD transition, with kidneys harvested at 14 days postinjury. **A**, Renal function parameters (serum creatinine, uric acid, and

BUN) were assessed on day 14 postinjury. **B** Histopathological assessment of kidney sections was done using H&E, and Masson's trichrome staining (MTS). Scale bar=100 μm. Representative data from one of three independent experiments are shown. Two-way ANOVA was used for statistical analysis. ***$p<0.001$. NS, not significant.

**Supplementary Figure 6**

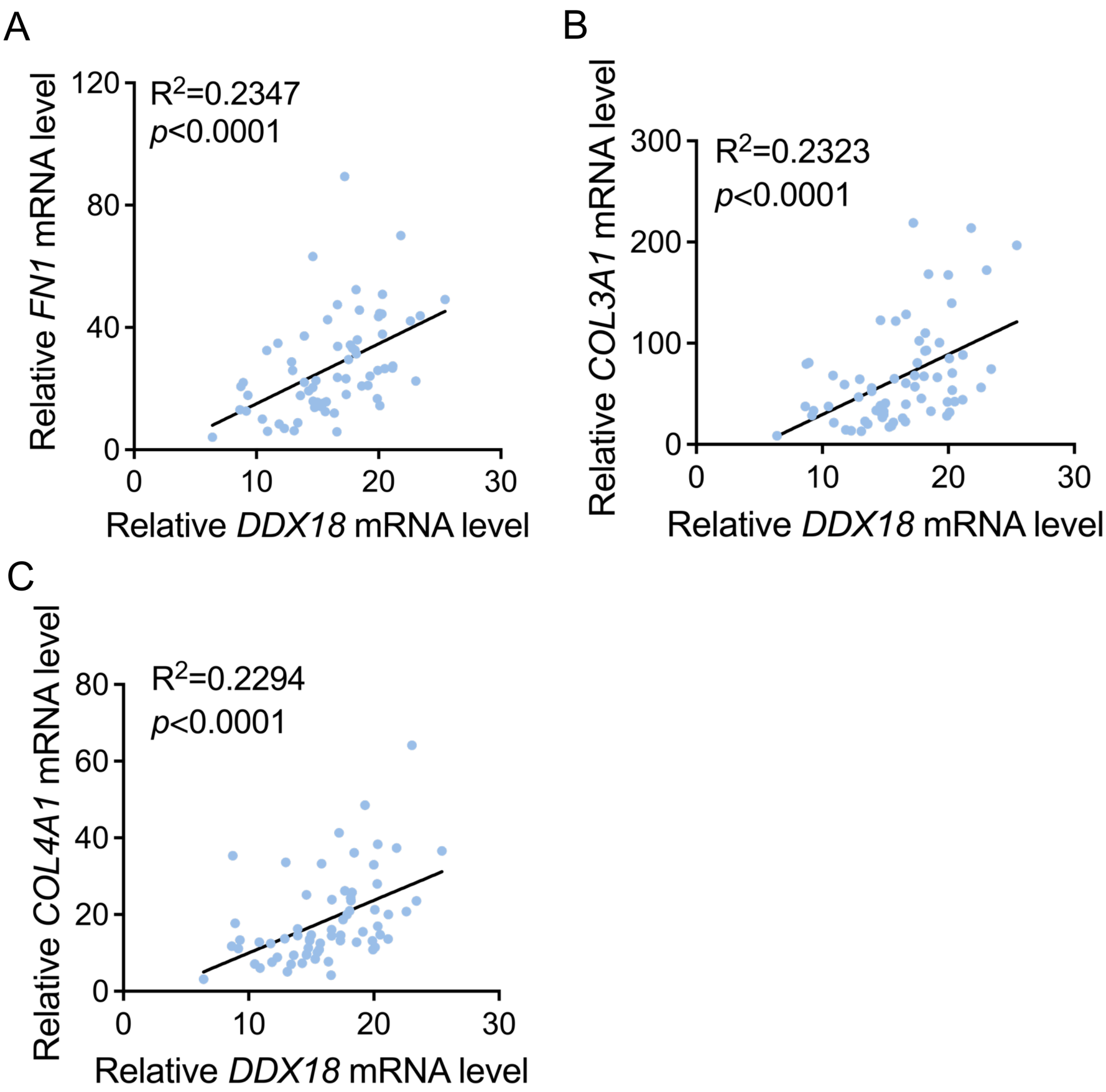


**Figure S6. Correlation analysis of DDX18 mRNA with fibrosis-related genes in the kidney of CKD patients**

Linear correlation analysis between DDX18 expression and fibrosis-associated genes **(A)** *FN1*, **(B)** *COL3A1*, **(C)** *COL4A1* in the kidney of CKD patients was conducted using normalized gene expression data retrieved from the GEO database (GSE175759). *P* value was calculated by Pearson's correlation analysis.

**Supplementary Figure 7**

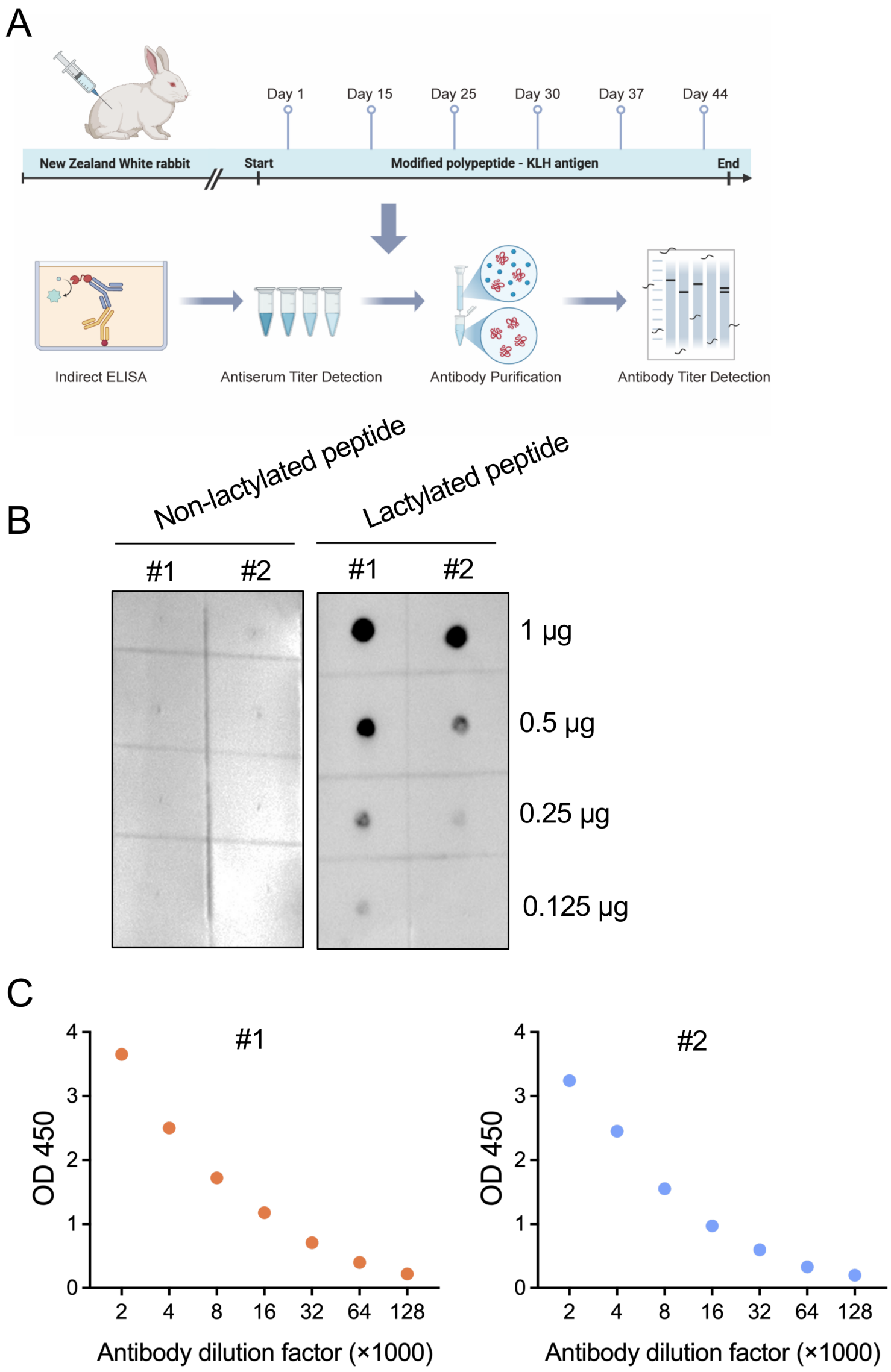


**Figure S7. Generation and validation of the DDX18-K116 lactylation-specific antibody**

**A**, Immunization protocol for generating anti-DDX18-K116lac antibodies. Rabbits were immunized with KKRK(lac)MVNDAEPDTK-Cys-BSA conjugates (0.5 mg/dose) according to the schedule shown. Antisera were collected and affinity-purified using antigen-coupled chromatography columns. **B**, Dot blot analysis demonstrating antibody specificity. Serial dilutions of lactylated nonlactylated (KKRKMVNDAEPDTK-Cys-BSA) and lactylated (KKRK(lac)MVNDAEPDTK-Cys-BSA) peptides were spotted on nitrocellulose membranes and probed with purified antibodies from two immunized

rabbits (#1 and #2), respectively, which revealed the selective recognition of the lactylated epitope. **C**, Indirect ELISA quantification of the antibody titer using plate-bound lactylated peptide antigen.

**Supplementary Figure 8**

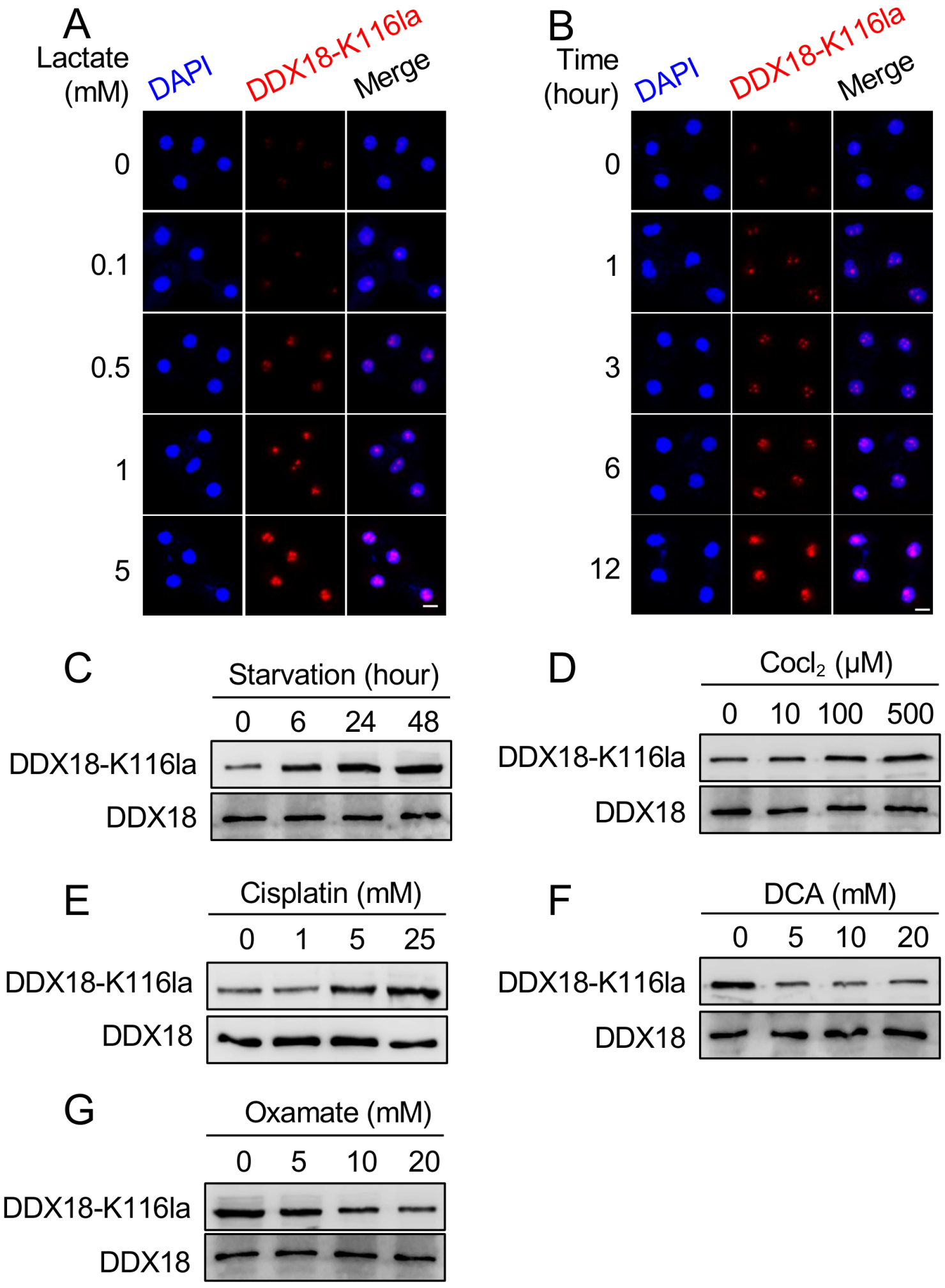


**Figure S8. DDX18-K116 lactylation is dynamically regulated by metabolic and cellular stress in TECs**

**A**, Immunofluorescence analysis of DDX18-K116lac levels in HK-2 cells exposed to increasing concentrations of exogenous lactate (0-5 mM) for 24 hours. Scale bar=20 μm. **B**, Time-course immunofluorescence assessment of DDX18-K116lac following treatment with 5 mM lactate for the indicated durations. Scale bar=20 μm. **C-G**, Western blot analysis of DDX18 K116 lactylation in HK-2 cells subjected to various stress conditions: serum starvation **(C)**, $CoCl_2$ **(D)**, cisplatin **(E)**, DCA **(F)**, and oxamate **(G)**. Data from one representative experiment of three independent experiments are presented.

**Supplementary Figure 9**

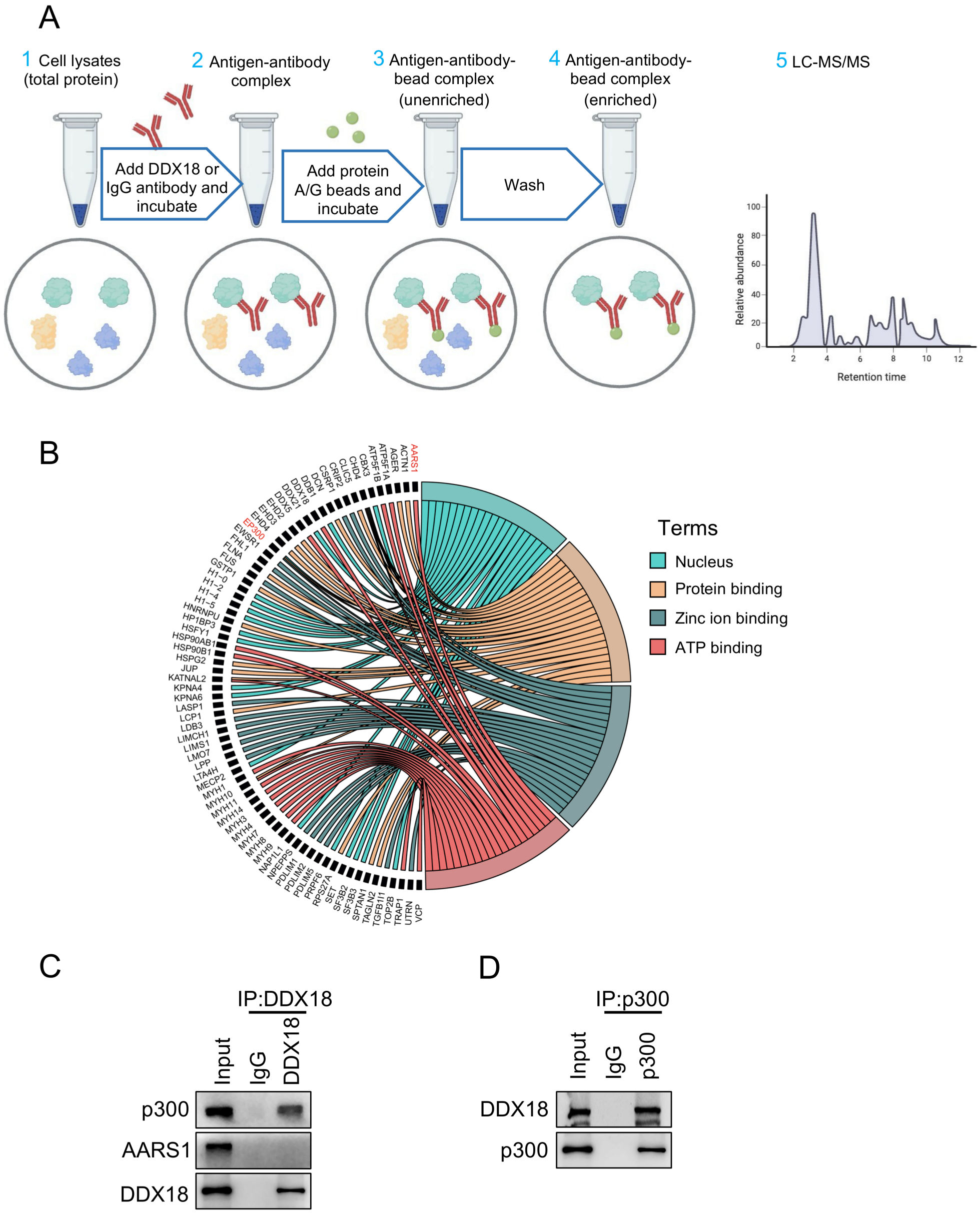


**Figure S9. Proteomic identification and characterization of the DDX18-interacting proteins**

**A**, Schematic workflow for identifying DDX18-interacting proteins using immunoprecipitation coupled with mass spectrometry (IP-MS). DDX18 complexes were immunoprecipitated from HK-2 cell lysates using anti-DDX18 antibodies, followed by on-bead tryptic digestion and LC-MS/MS analysis. **B**, Subcellular distribution of DDX18-associated proteins identified by IP-MS and InterPro domain enrichment analysis of the DDX18 interactome. **C**, Validation of interactions between DDX18

and known lactylation/acetylation regulatory enzymes by coimmunoprecipitation. HK-2 cell lysates were immunoprecipitated with an anti-DDX18 antibody, followed by immunoblotting for the indicated proteins. **D**, Reciprocal coimmunoprecipitation confirming the DDX18-p300 interaction. The cell lysates were immunoprecipitated with an anti-p300 antibody and immunoblotted for DDX18. Data from one representative experiment of three independent experiments are presented.

**Supplementary Figure 10**

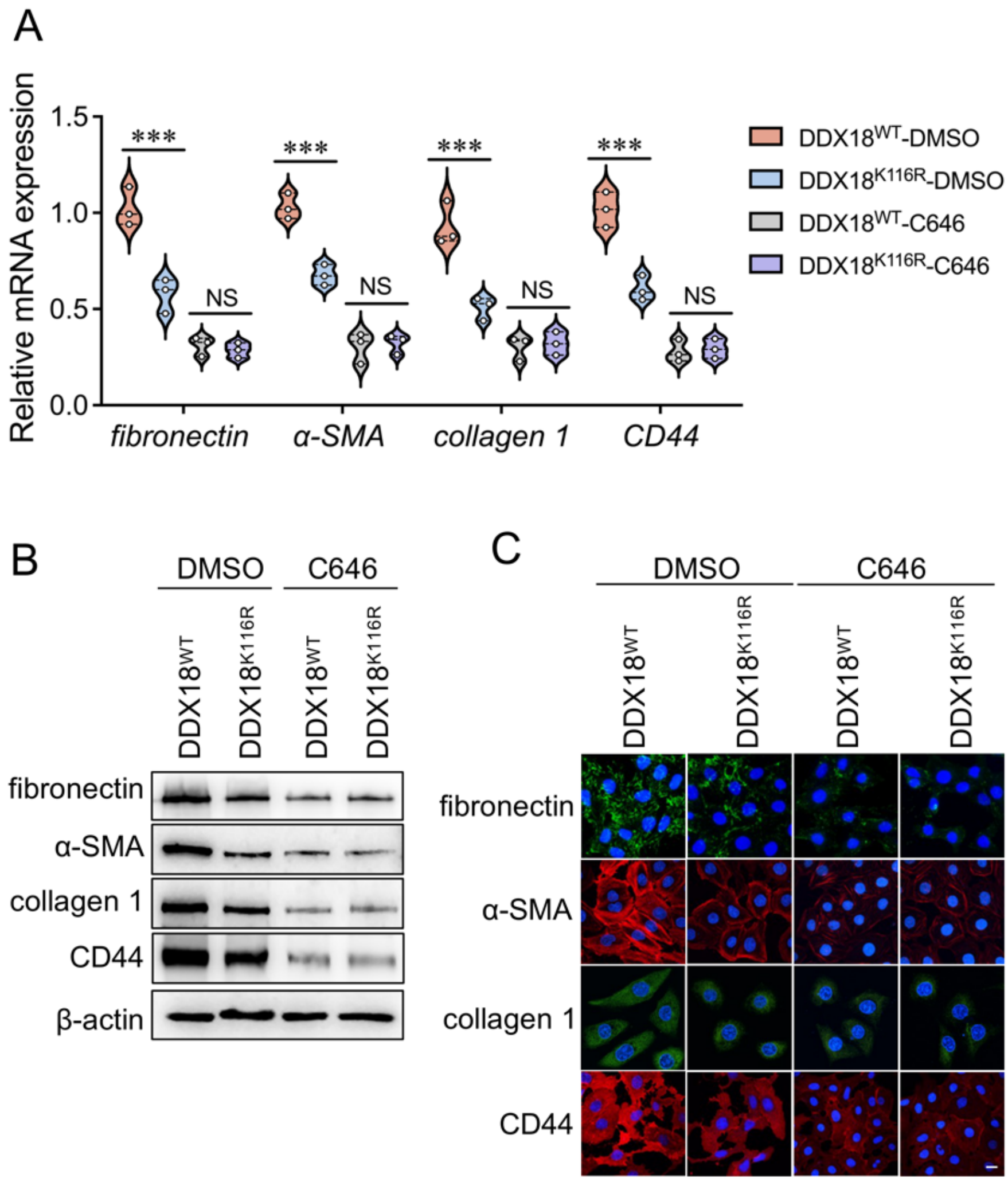


**Figure S10. Pharmacological inhibition of p300 attenuates lactylation-dependent fibrotic responses in TECs**

HK-2 cells overexpressing DDX18$^{WT}$ or DDX18$^{K116R}$ were treated with DMSO or C646 (1 mM) for 24 hours under normoxia or H/R conditions. The expression levels of *fibronectin*, *α-SMA*, *collagen 1*, and *CD44* were evaluated in HK-2 cells through quantitative RT-PCR **(A)**, immunoblotting **(B)** and immunofluorescence **(C)**. Representative data from one of three independent experiments are shown. *P* value was calculated by two-way ANOVA. ***p<0.001. NS, not significant.

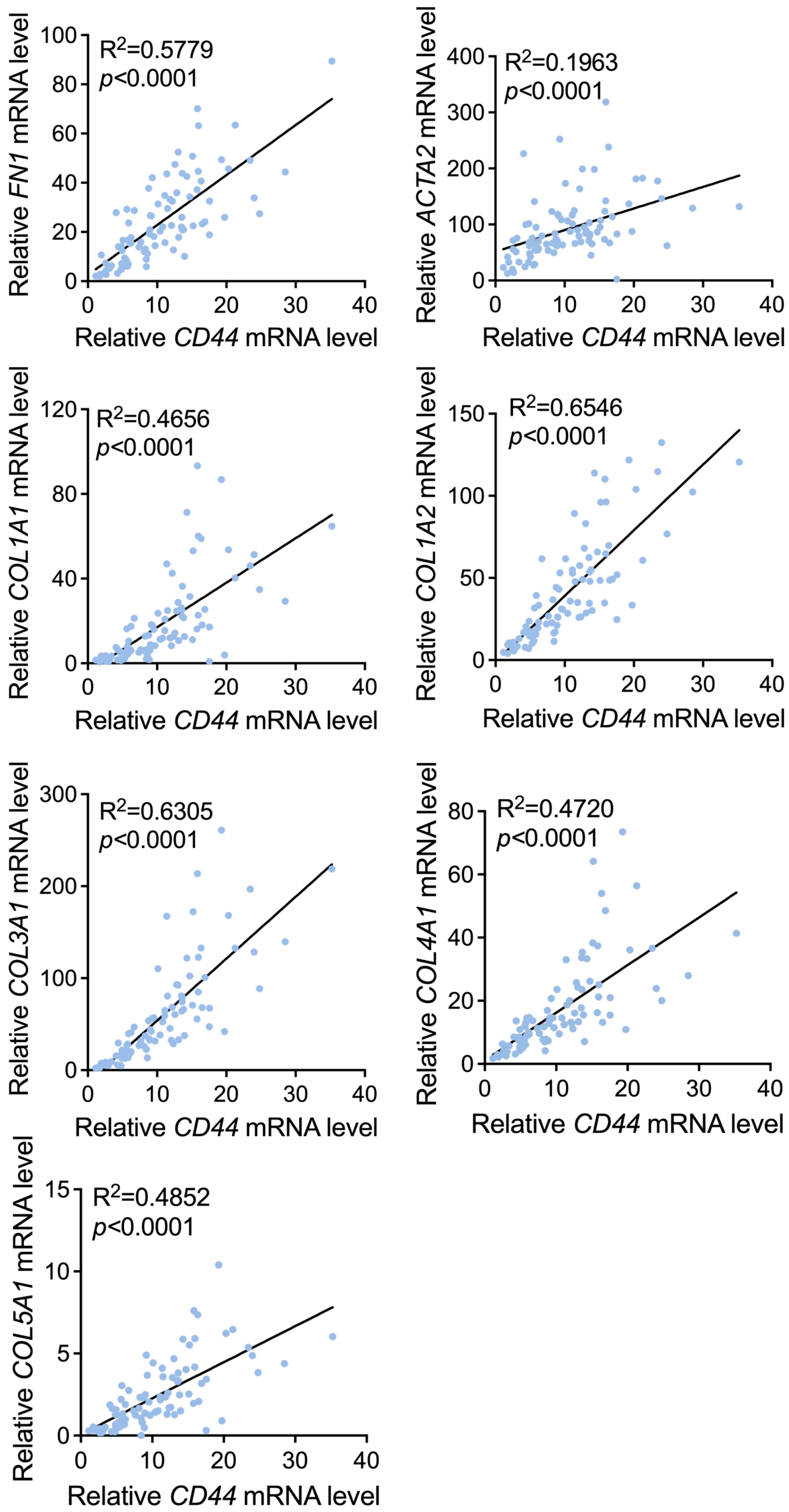


**Figure S11. Correlation analysis of *CD44* mRNA with fibrosis-related genes in the kidney of CKD patients**

Linear correlation analysis between *CD44* expression and fibrosis-associated genes (*FN1, ACTA2, COL1A1, COL2A1, COL3A1, COL4A1, COL5A1*) in the kidney of CKD patients was conducted using normalized gene expression data retrieved from the GEO database (GSE175759). *P* value was calculated by Pearson's correlation analysis.

**Supplementary Figure 12**

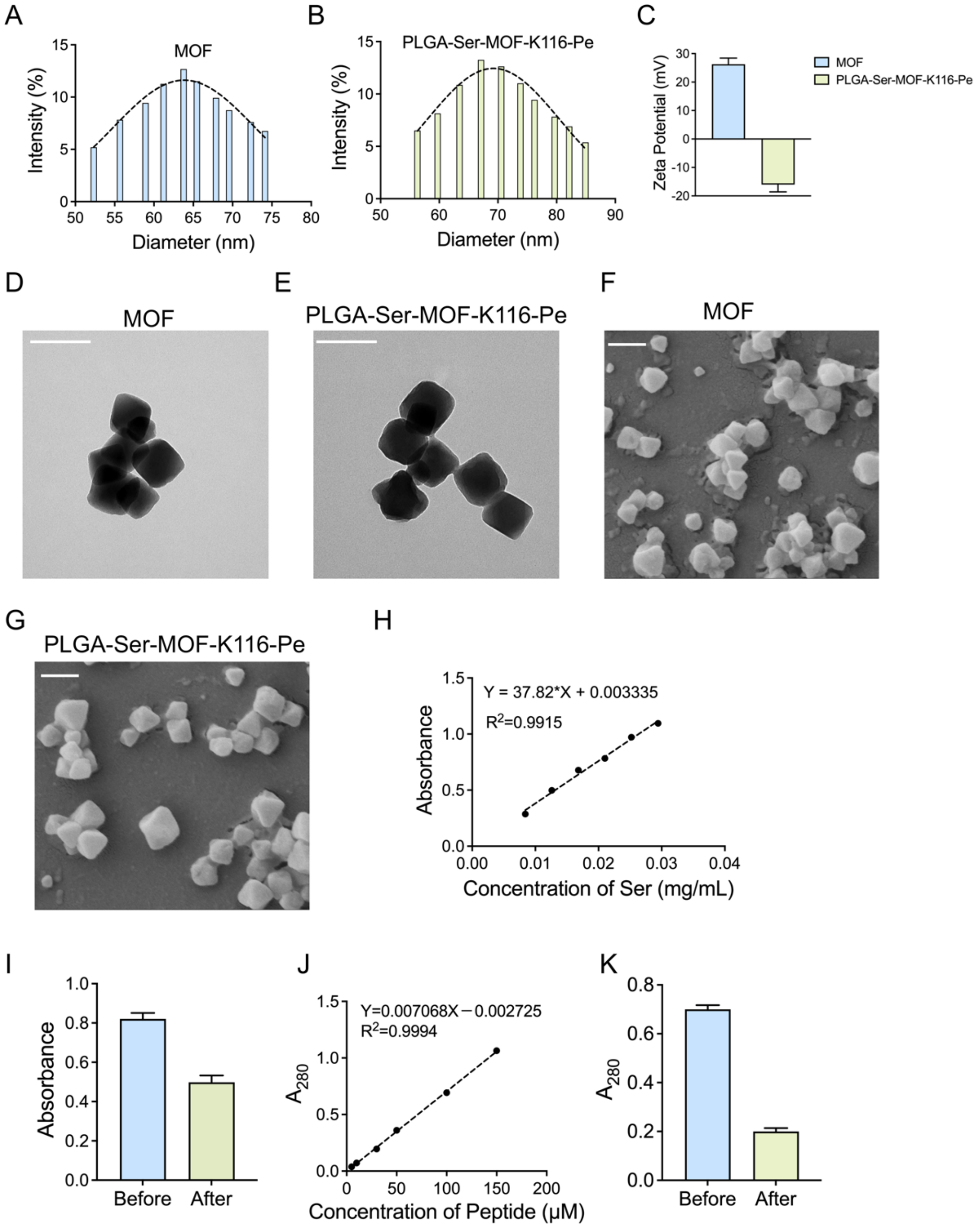


**Figure S12**. **Characterization results of kidney-targeted MOF-based nanocomposites**

**A**, Particle size distribution of the MOF. **B**, Particle size distribution of PLGA-Ser-MOF-K116-Pe. **C,** Zeta potential results of MOF and PLGA-Ser-MOF-K116-Pe. **D**, **E**, Transmission electron microscopy (TEM) images of MOF and PLGA-Ser-MOF-K116-Pe. Scale bar=100 nm. **F**, **G**, Scanning electron microscopy (SEM) images of MOF and PLGA-Ser-MOF-K116-Pe. Scale bar=100 nm. **H**, Standard

calibration curve of serine. **I**, Changes in serine concentration before and after MOF modification. **J**, Standard calibration curve of the peptides. **K**, Changes in peptide concentration before and after MOF modification.

Supplementary Figure 13

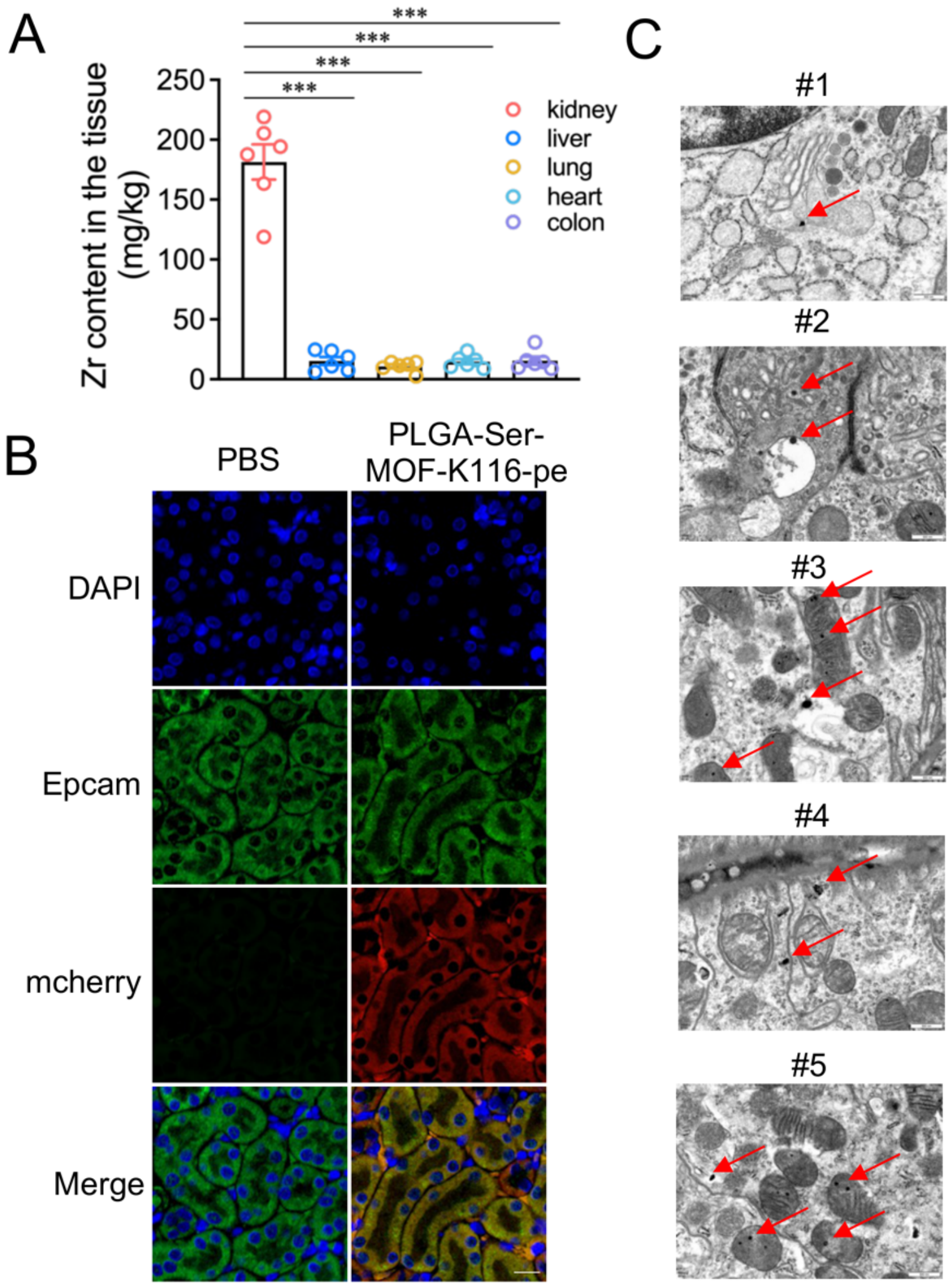


**Figure S13. Distribution of PLGA-Ser-MOF-K116-pe in the mouse kidney**

C57BL/6J mice were intravenously injected with PLGA-Ser-MOF-K116-pe (0.5mg/kg, 0.1mg/mL), its distribution in the kidney was evaluated by ICP-MS measurement of Zr content in multiple organs **(A)**, immunofluorescence analysis of renal tubular epithelial cells **(B)**, and SEM observation of particle localization in the kidney **(C)**. Representative data from one of three independent experiments are shown. One-way ANOVA was used for data in A statistical analysis.

**Supplementary Figure 14**

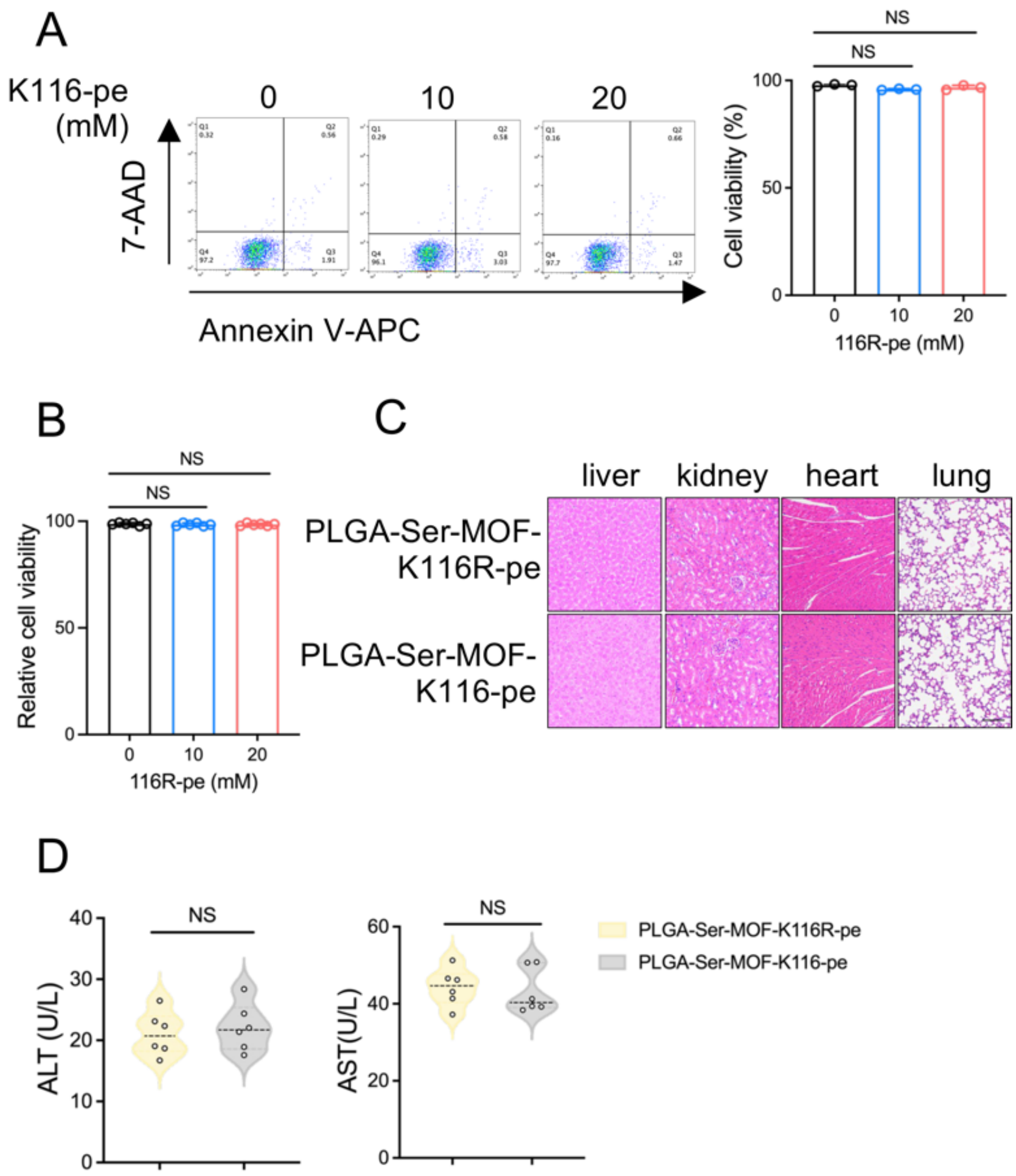


**Figure S14. Safety profile of the DDX18-K116 lactylation-blocking peptide *in vitro* and *in vivo***

**A**, **B**, Cytotoxicity assessment of the K116 peptide in HK-2 cells. The cells were treated with increasing concentrations of K116 peptide (0-20 μM) for 24 hours, and viability was assessed *via* flow cytometry with Annexin V/PI staining (**A**) and a CCK-8 proliferation assay **(B)**. **C**, **D**, *In vivo* safety evaluation of the PLGA-Ser-MOF-K116 peptide. Male C57BL/6J mice (n=6 per group) received PLGA-Ser-MOF-K116-peptide or PLGA-Ser-MOF-K116R-peptide (control) every other day for 14 days. Representative H&E-stained sections of kidney, liver, heart, and lung tissues showing normal histological architecture in both treatment groups. Scale bar=100 μm (**C**). Serum alanine aminotransferase (ALT) and aspartate aminotransferase (AST) levels demonstrating preserved liver

function in peptide-treated mice (**D)**. Representative data from one of three independent experiments are shown. One-way ANOVA was used for statistical analysis in data **A**, and **B**. Unpaired two-tailed Student's t-test was used for statistical analysis in data **D**. NS, not significant.

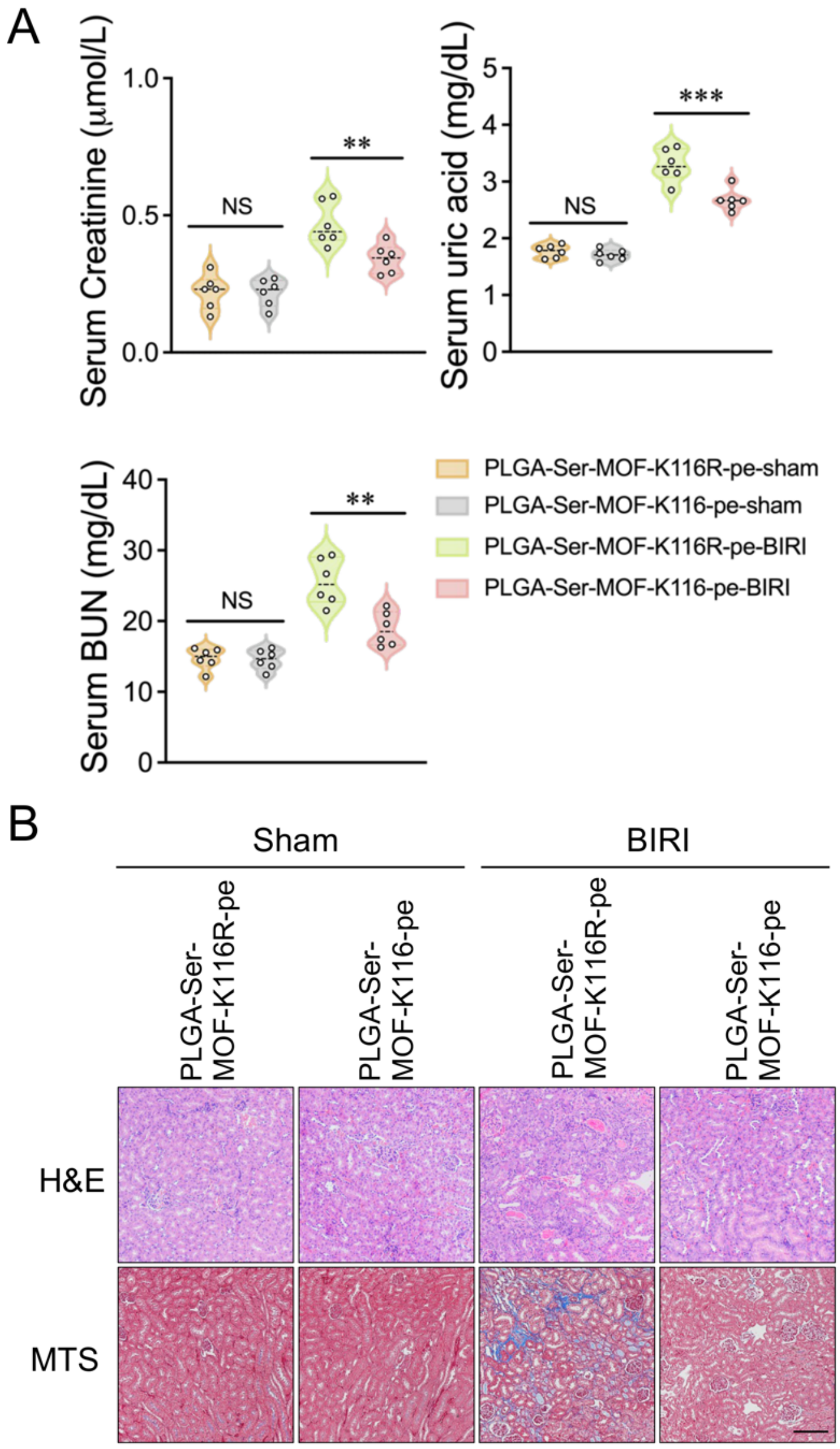


**Figure S15. K116-targeted peptide repairs I/R induced kidney dysfunction in female mice**

Female C57BL/6J mice (n=6 per group) received PLGA-Ser-MOF-K116-peptide or PLGA-Ser-MOF-K116R-peptide (control) every other day for 14 days. **A,** Renal function parameters (serum creatinine, uric acid, and BUN) were assessed on day 14 postinjury. **B**, Histopathological assessment of kidney sections using H&E and Masson's trichrome staining. Scale bar=100 μm. Representative data from

one of three independent experiments are shown. Two-way ANOVA was used for data **A** statistical analysis.

**Table S1. Baseline creatinine according to lactate group among participants with AKI within 1 year before recruitment**

| Characteristic | Low lactate ≤4.135mmol/L | High lactate > 4.135mmol/L | P value |
|---|---|---|---|
| Participants, n | 36 | 22 | |
| Baseline creatinine, mmol/L, median (IQR) | 0.092 (0.072-0.113) | 0.099 (0.078-0.116) | 0.557 |

**Table S2. Clinical characteristics of the participants**

| Variable | Total (N=29) | AKI with proteinuria (n=14) | AKI without proteinuria (n=15) | P value |
|---|---|---|---|---|
| Age, year (SD) | 50 (19.7) | 54.4 (5.1) | 50.4 (4.6) | 0.554 |
| Male, no. (%) | 18 (62.0) | 6 (33.3) | 5 (45.5) | 0.597 |
| Laboratory values | | | | |
| Blood hemoglobin, g/L (SD) | 118.1 (25.3) | 118.2(6.9) | 112.4 (7.9) | 0.862 |
| Serum creatinine, μmol/L (SD) | 366.5 (244.5) | 314.3 (45.7) | 452.5 (75.6) | 0.125 |
| Serum albumin, g/L (SD) | 27.9 (10.7) | 22.6 (2.3) | 36.3 (1.7) | <0.001 |
| Serum complement 3, g/L (SD) | 1.03 (0.23) | 1.08 (0.08) | 0.99 (0.06) | 0.336 |
| Urine protein excretion, g/24h (SD) | 4.37 (6.48) | 7.58 (1.84) | 0.17 (0.02) | <0.001 |

Data are presented as mean (SD) normally distributed continuous variables and number (%) for categorical variables.

**Table S3. Information List of DDX18 Modification Site Identification**

Detailed characteristics of DDX18 lactylation sites, including modification position and peptide sequence. This is provided as a separate file, Table S3.
