# Supplementary material for: Lactylation-dependent DDX18 nucleolar escape regulates CD44 mRNA stability to drive fibrotic progression in kidney injury
Source: J Clin Invest. 2026 Aug 13;136(19):e201848. doi: 10.1172/JCI201848 (PMC13626828; doi:10.1172/JCI201848)

Full unedited gel for Figure 1J

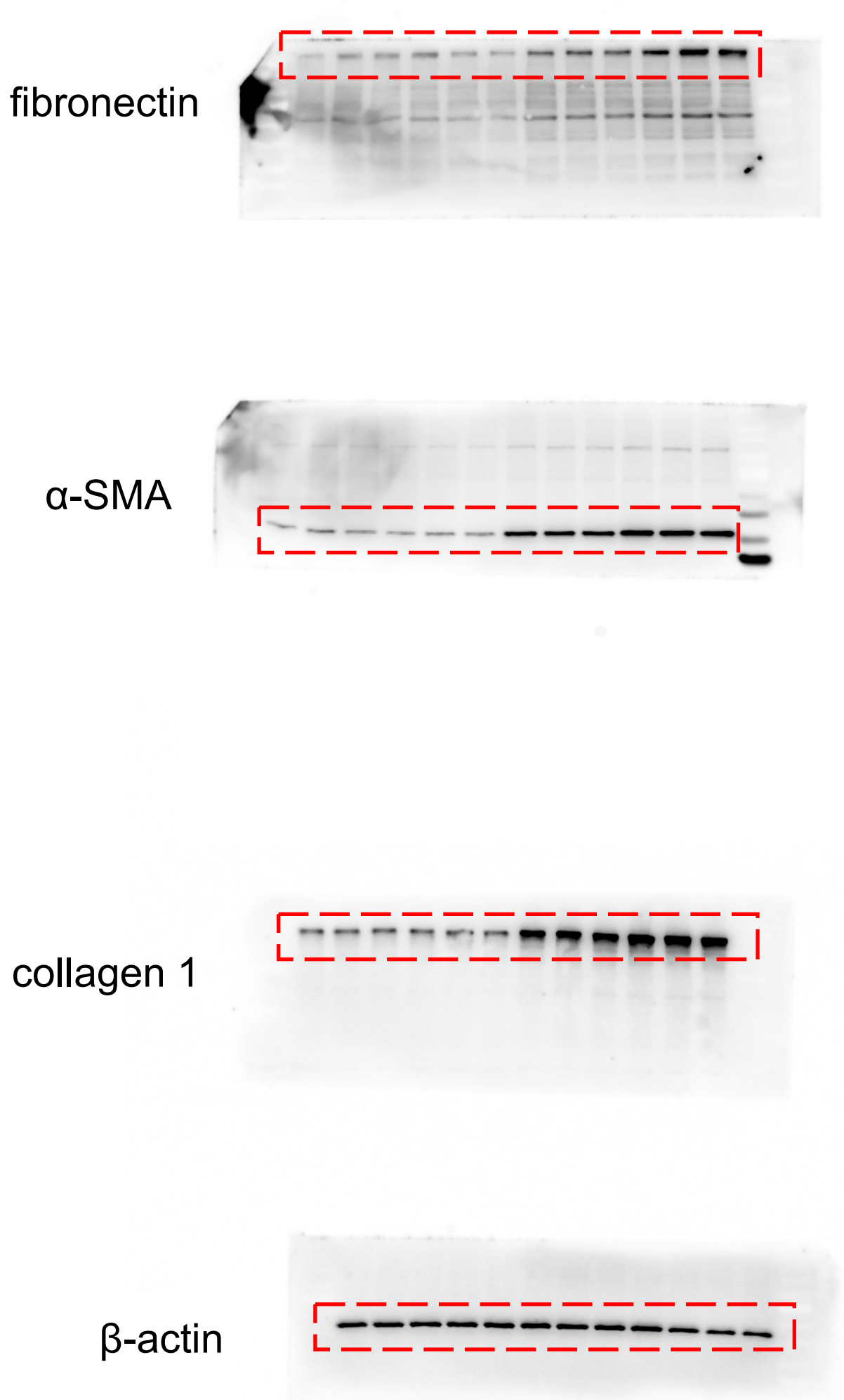

Full unedited gel for Figure 1P

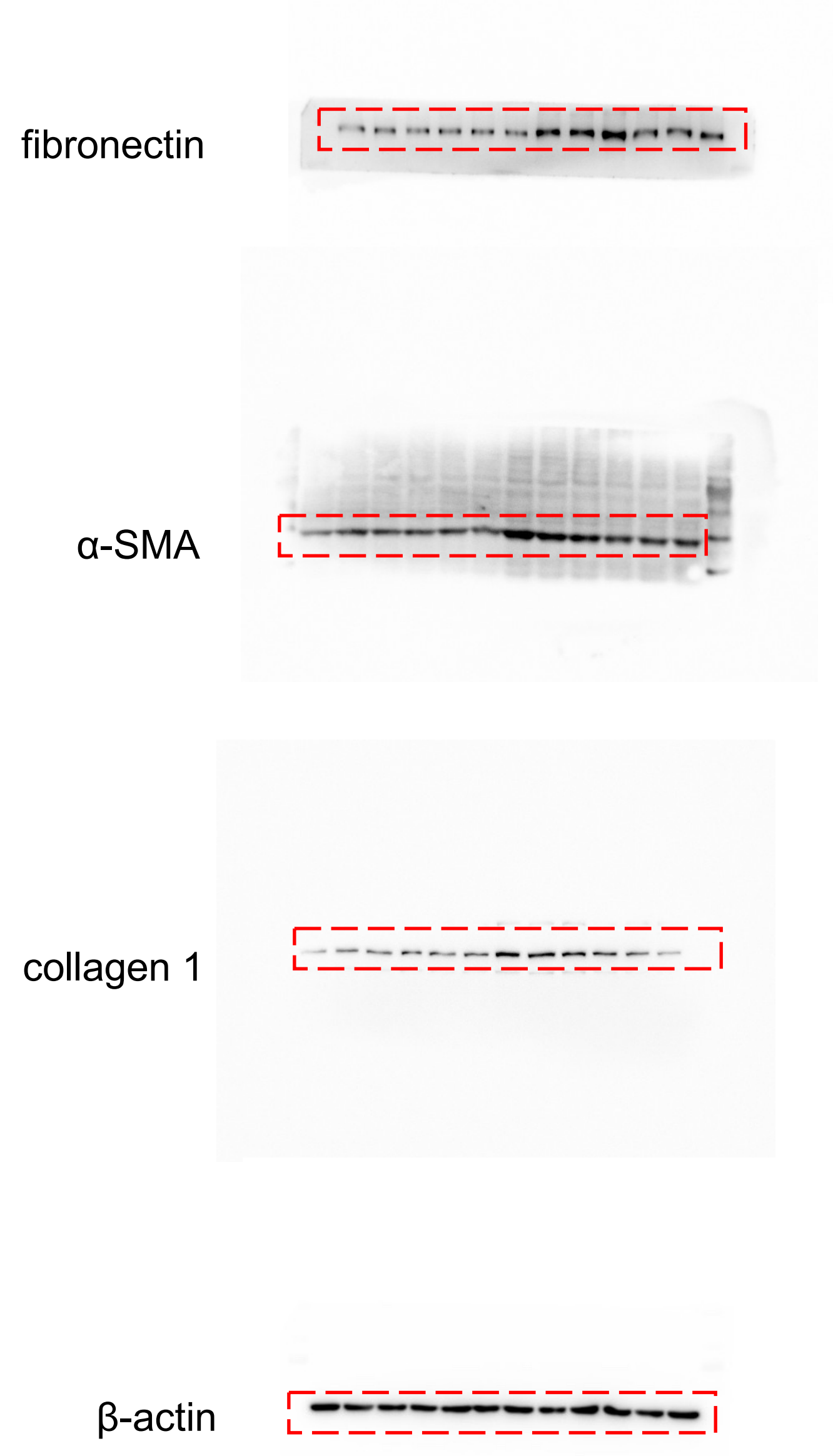

Full unedited gel for Figure 2C

Kla

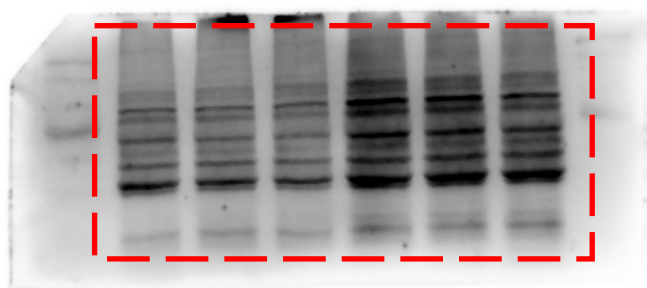

β-actin

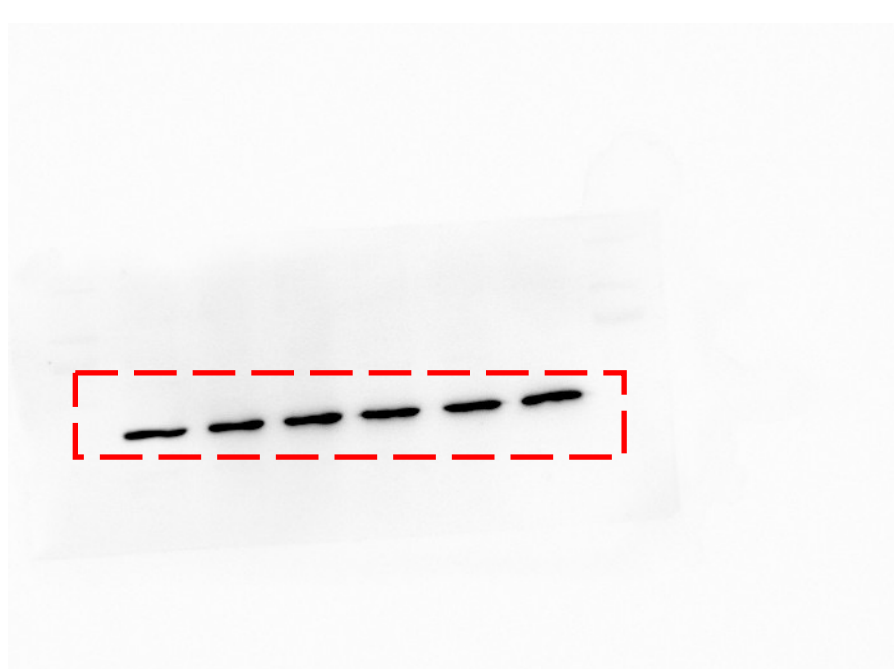

Full unedited gel for Figure 2D

Kla

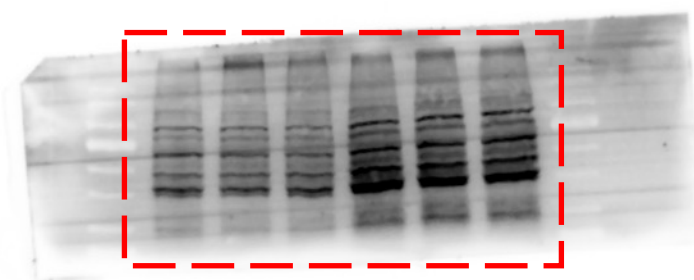

β-actin

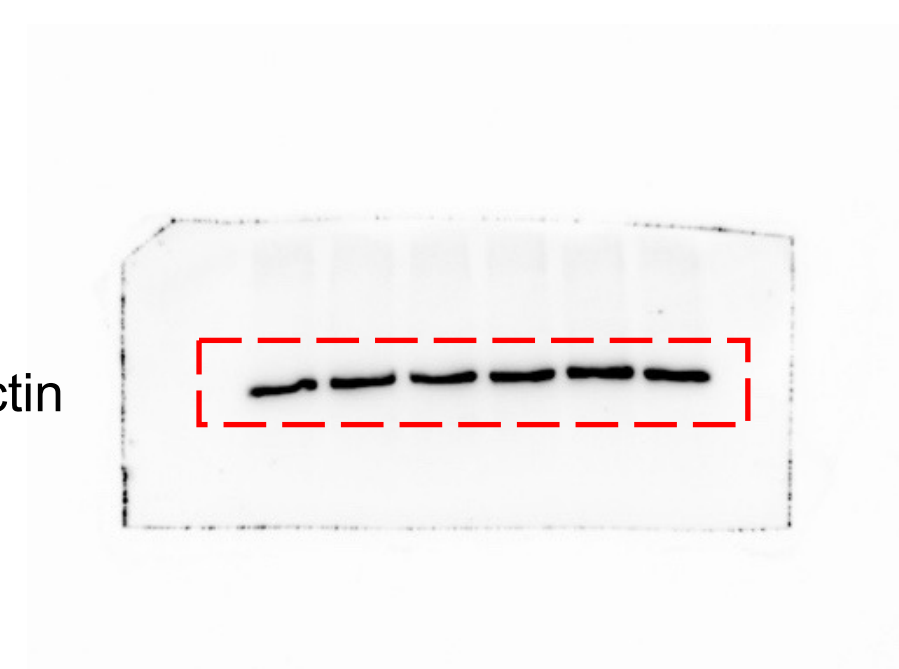

Full unedited gel for Figure 2K

DDX18

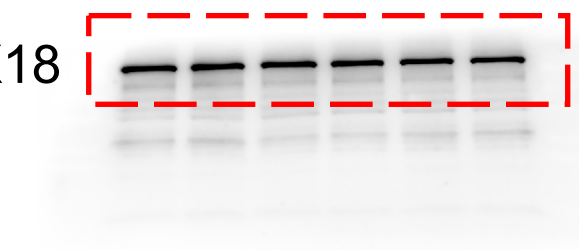

β-actin

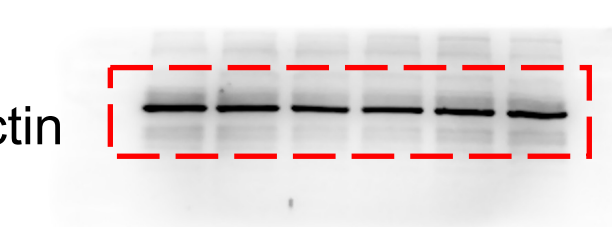

Full unedited gel for Figure 2L

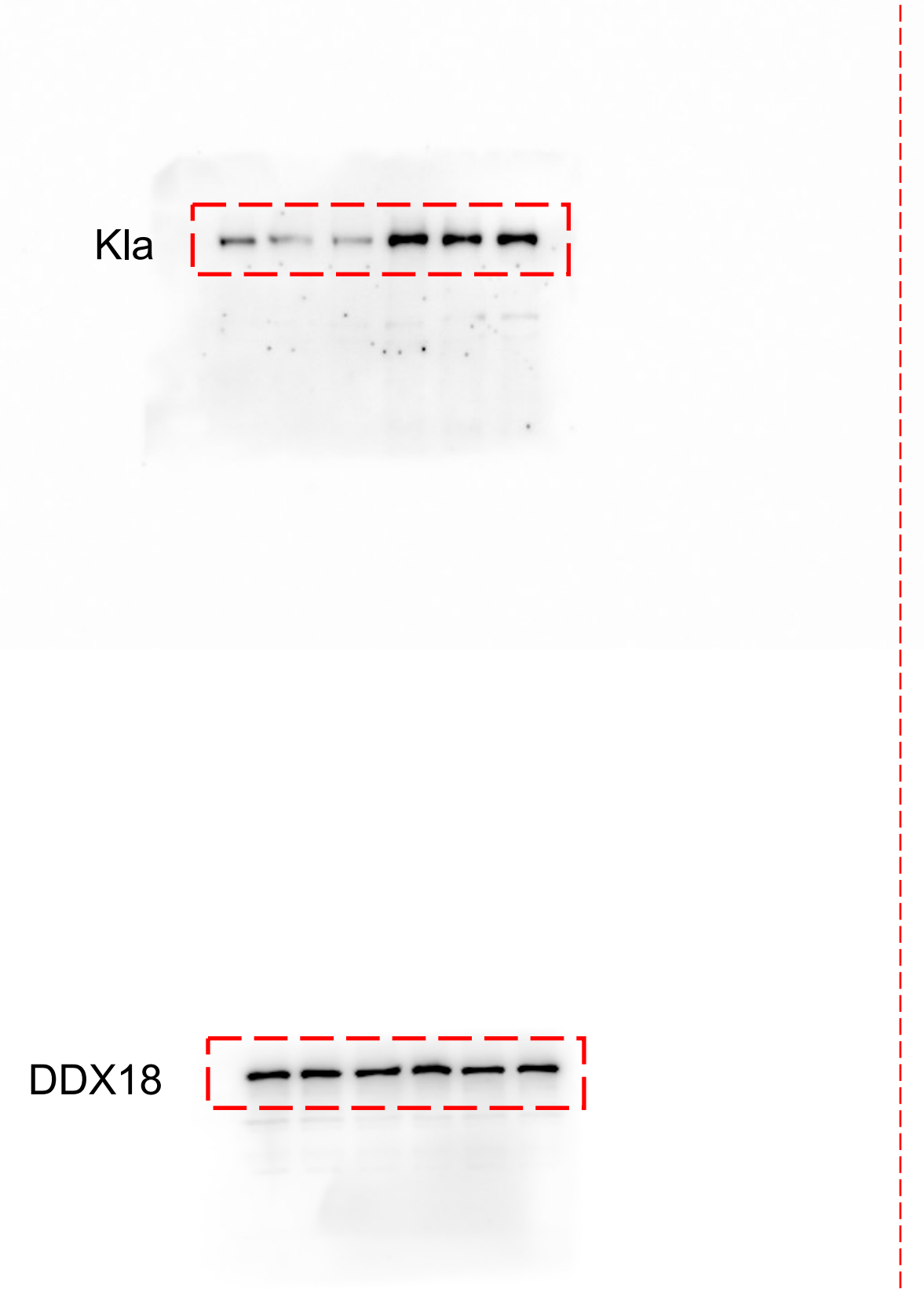

Full unedited gel for Figure 2M

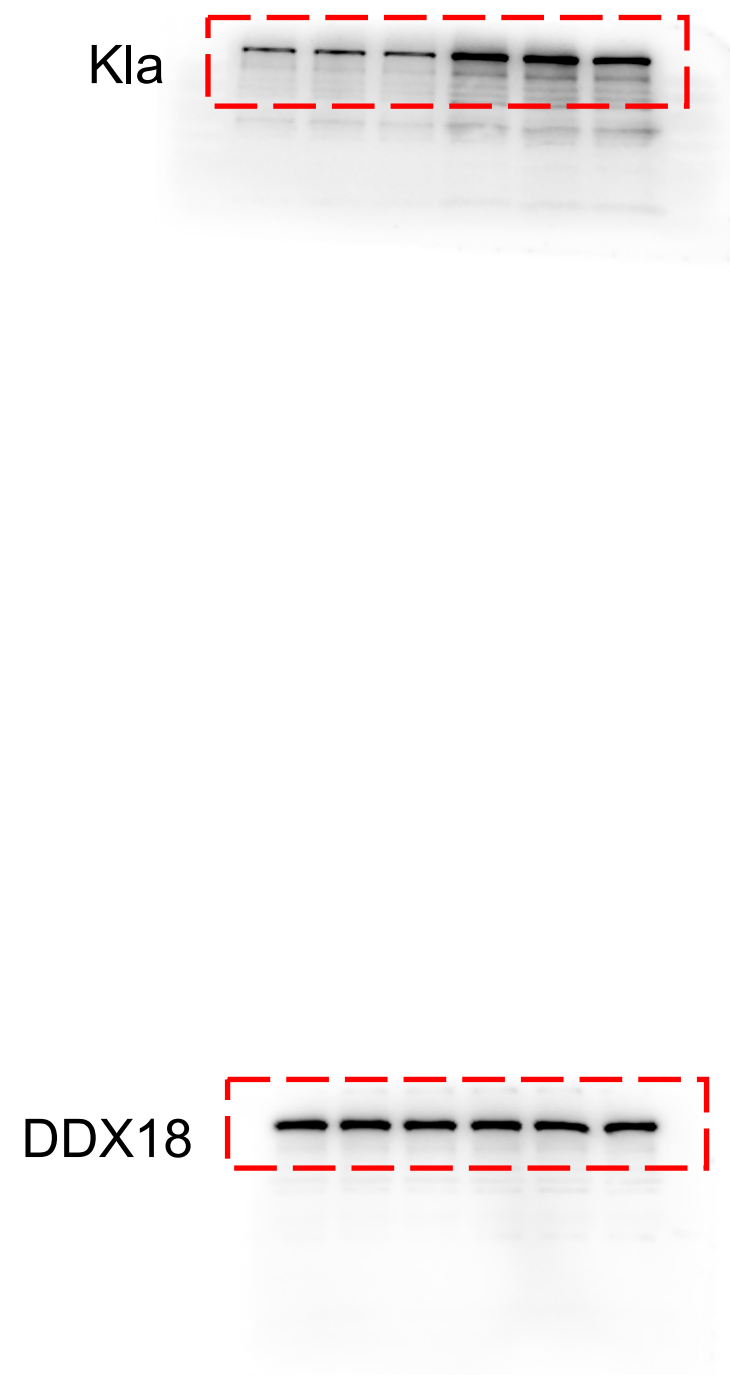

Full unedited gel for Figure 2N

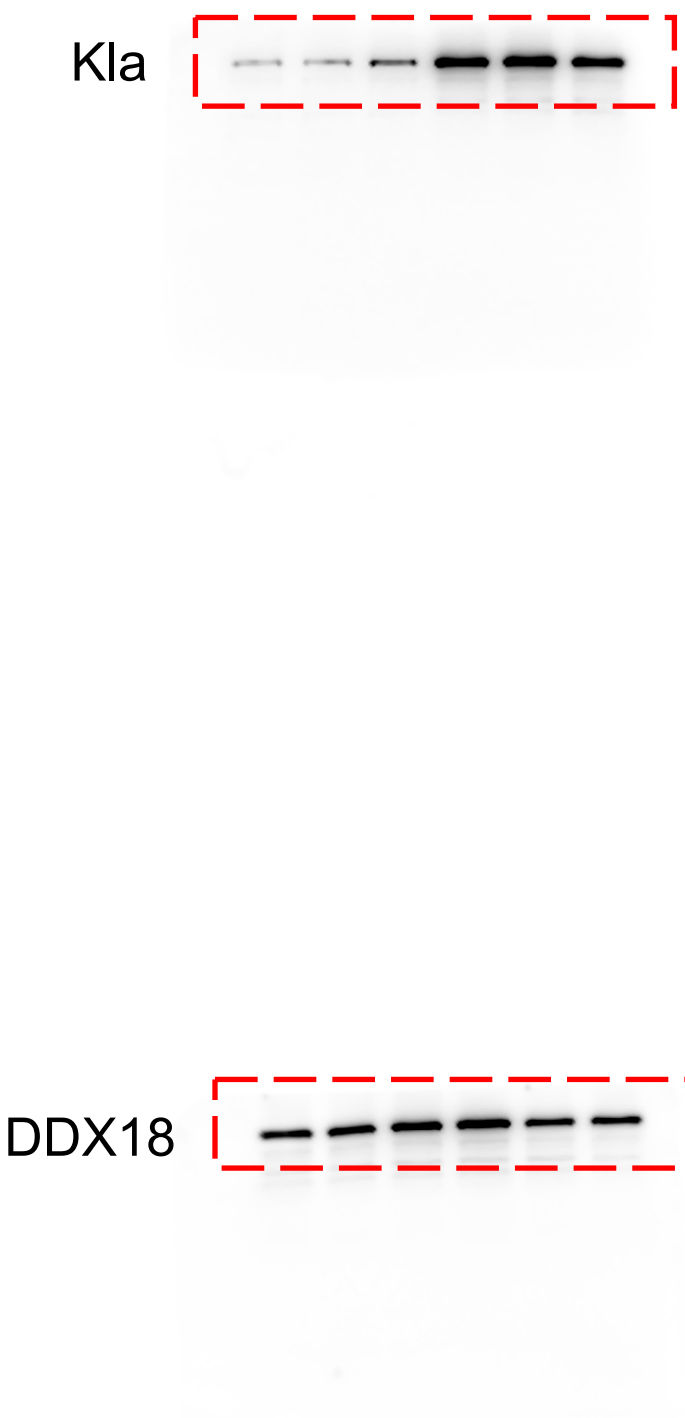

Full unedited gel for
Figure 3E

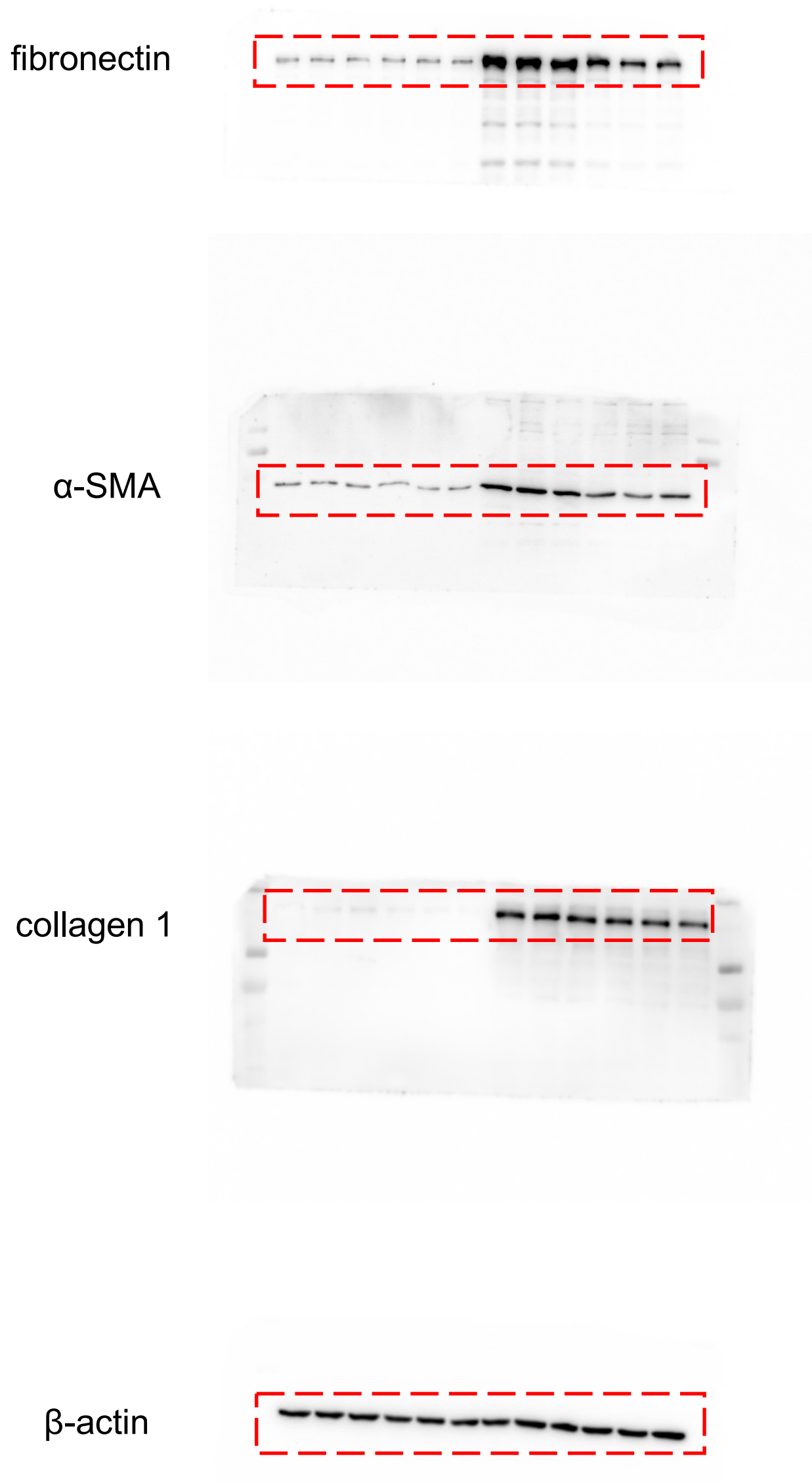

Full unedited gel for
Figure 3I

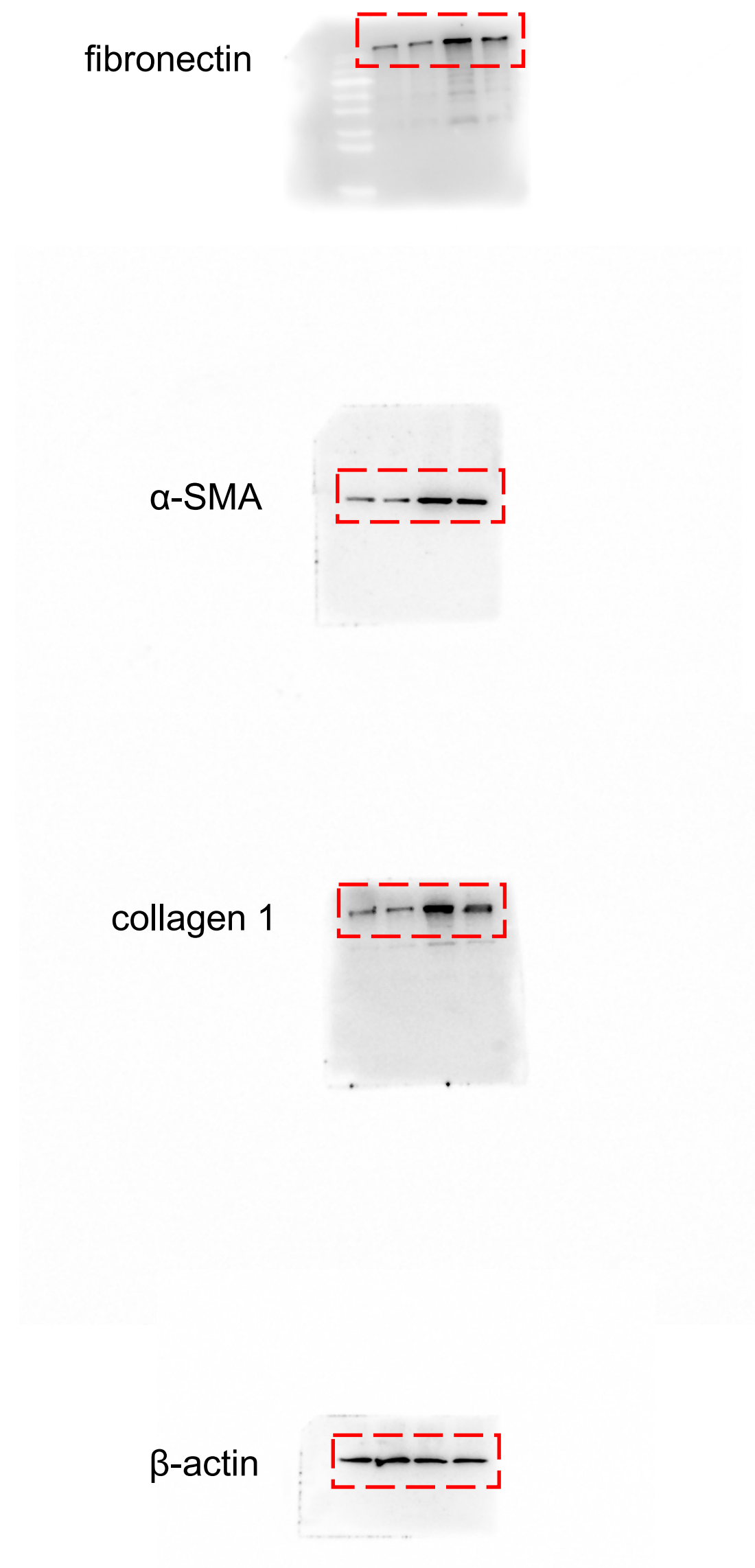

Full unedited gel for Figure 4C

IB:Kla

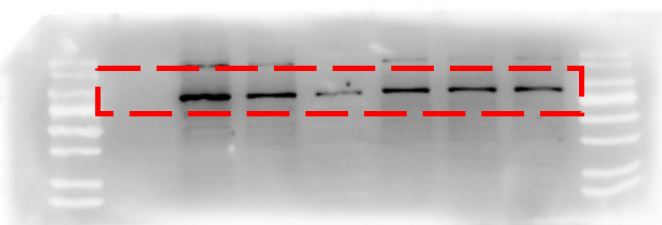

IB:DDX18

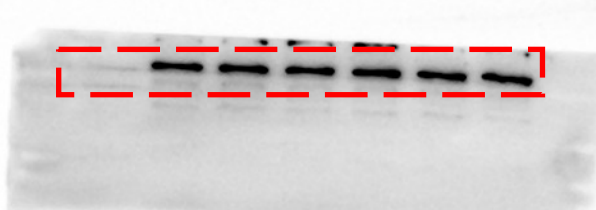

Full unedited gel for Figure 4E

IB:DDX18-K116la

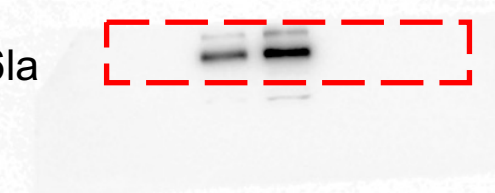

IB:DDX18

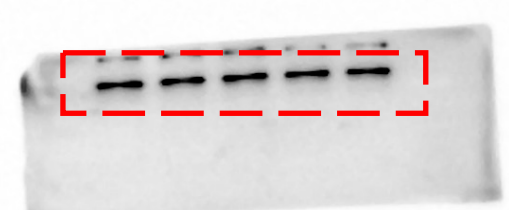

Full unedited gel for Figure 4F

DDX18-K116la

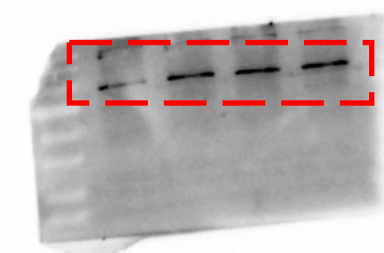

DDX18

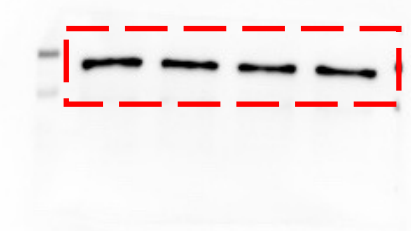

Full unedited gel for Figure 4G

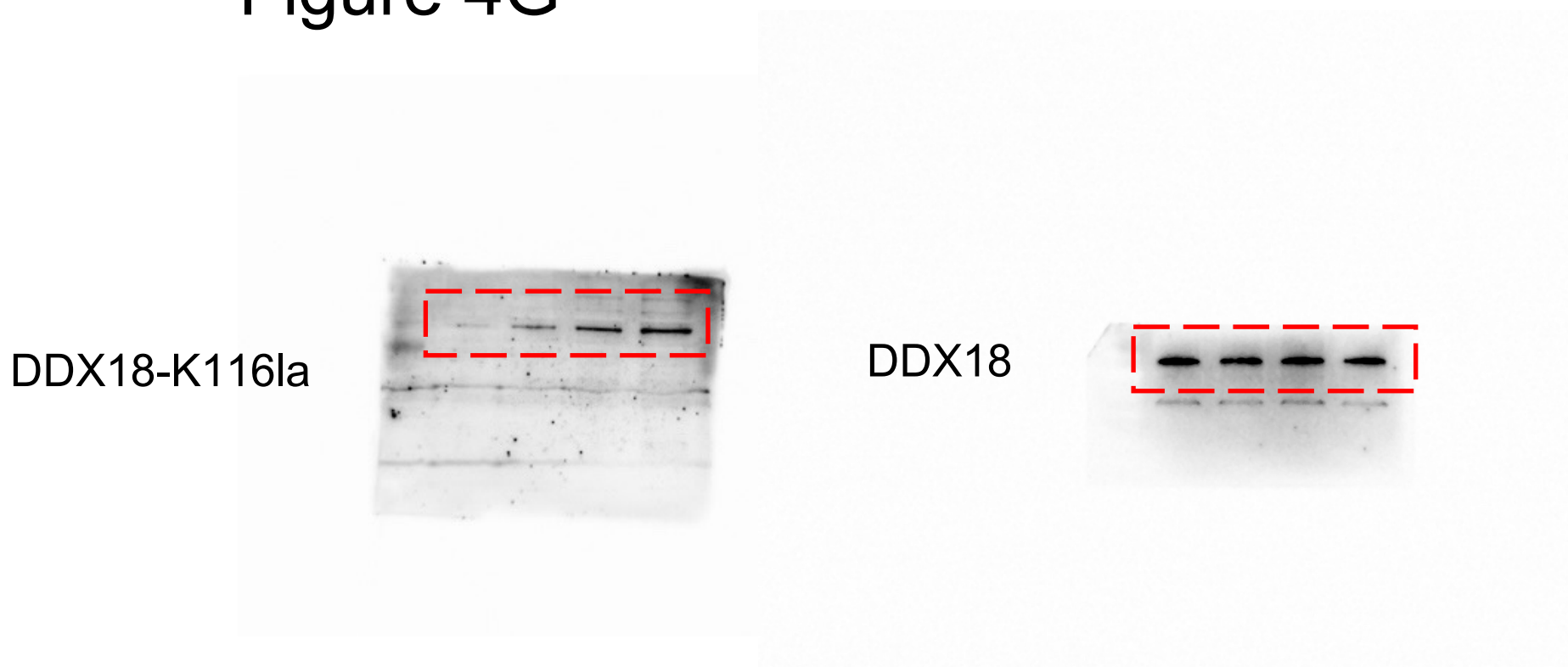

Full unedited gel for Figure 4M

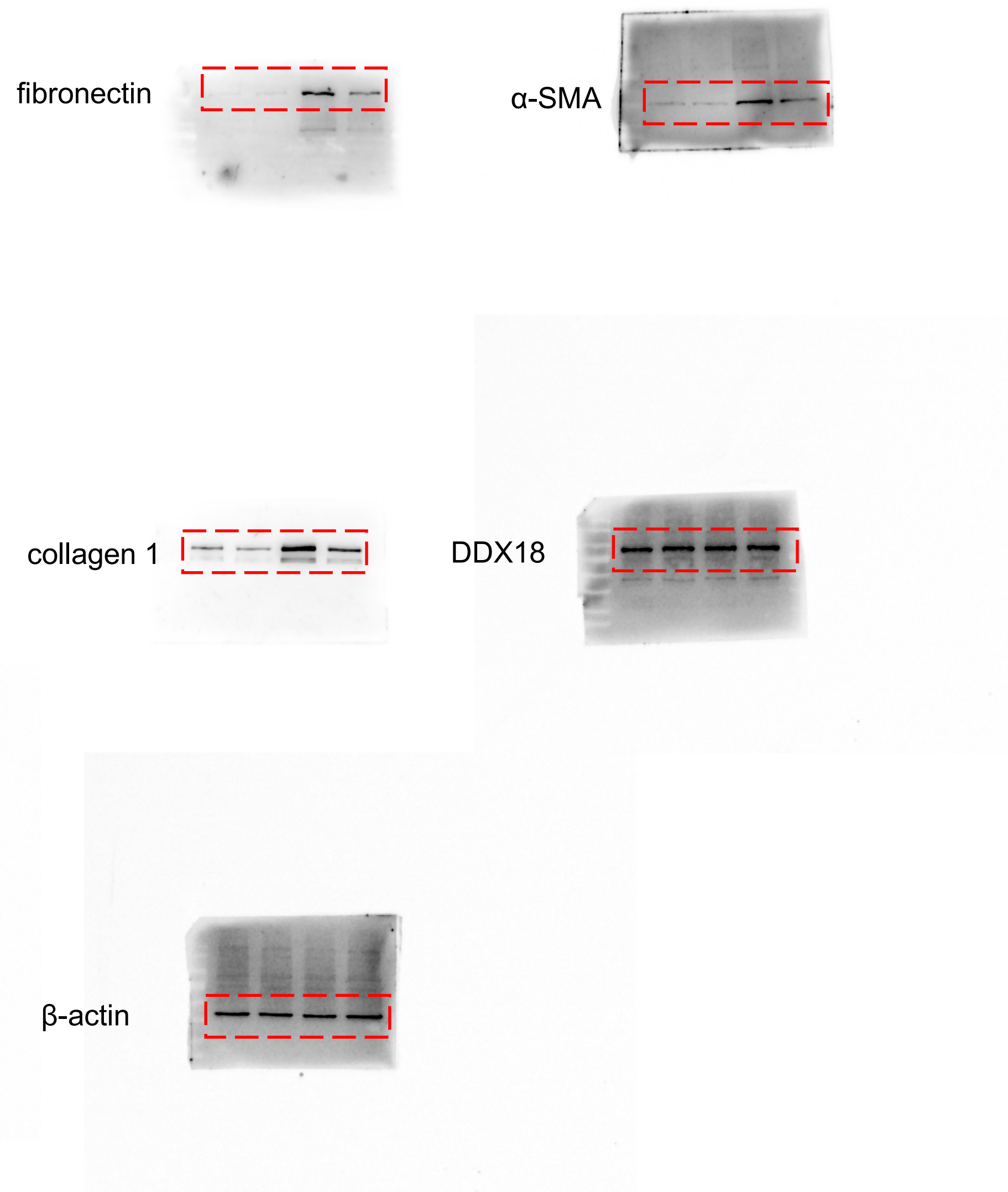

Full unedited gel for Figure 4H

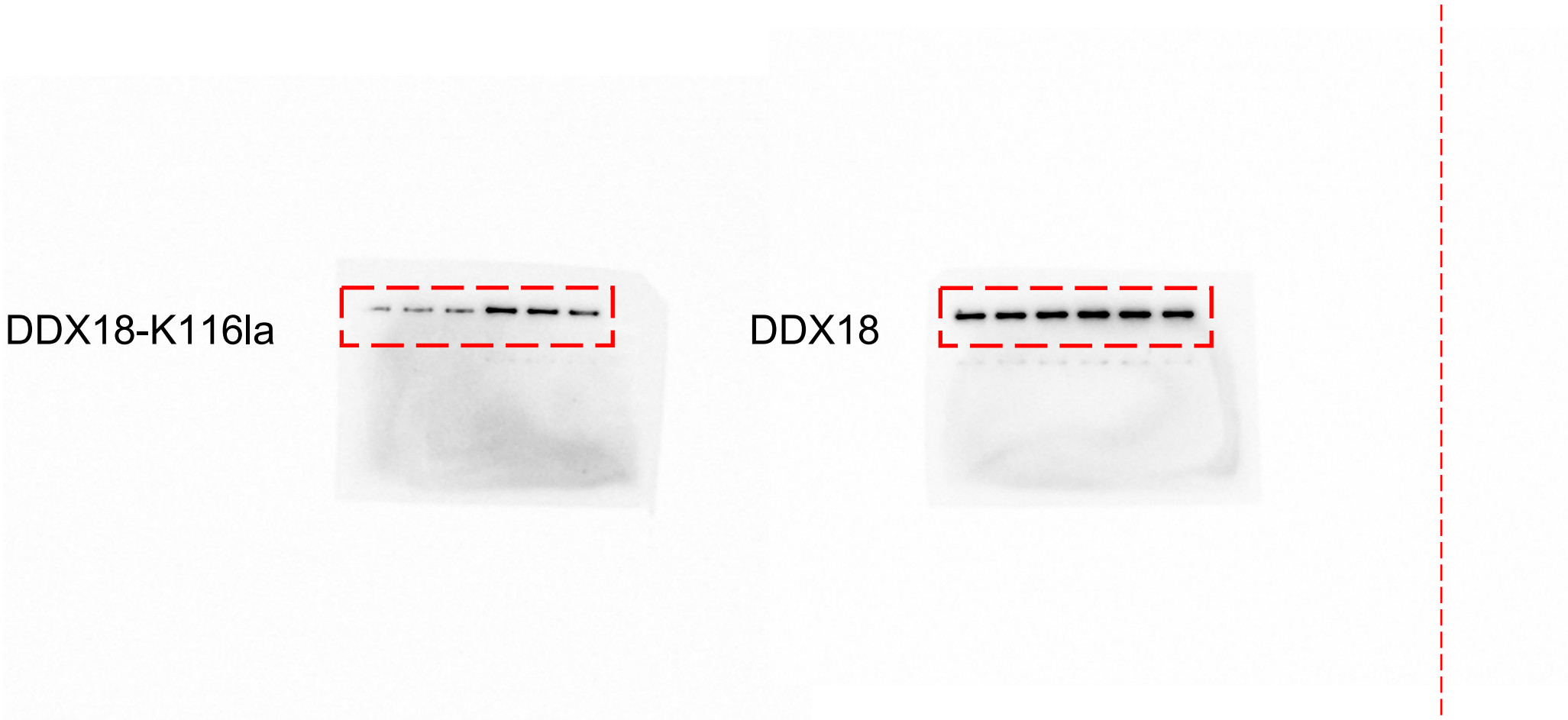

Full unedited gel for Figure 5B

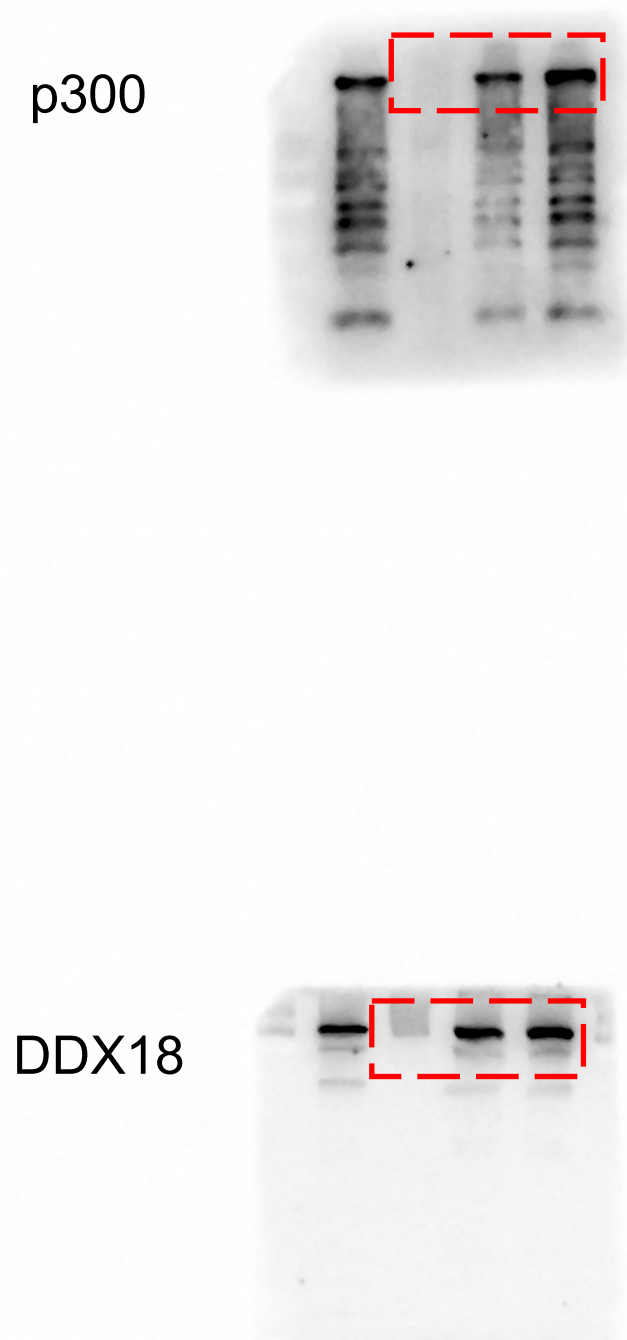

Full unedited gel for Figure 5C

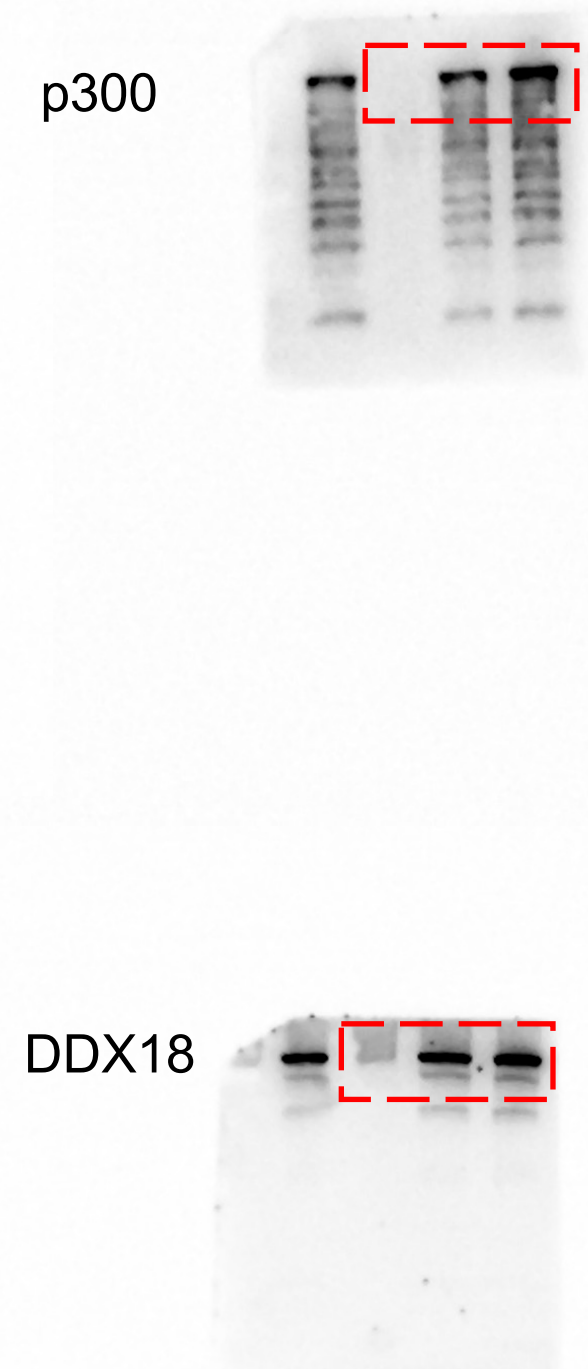

Full unedited gel for Figure 5D

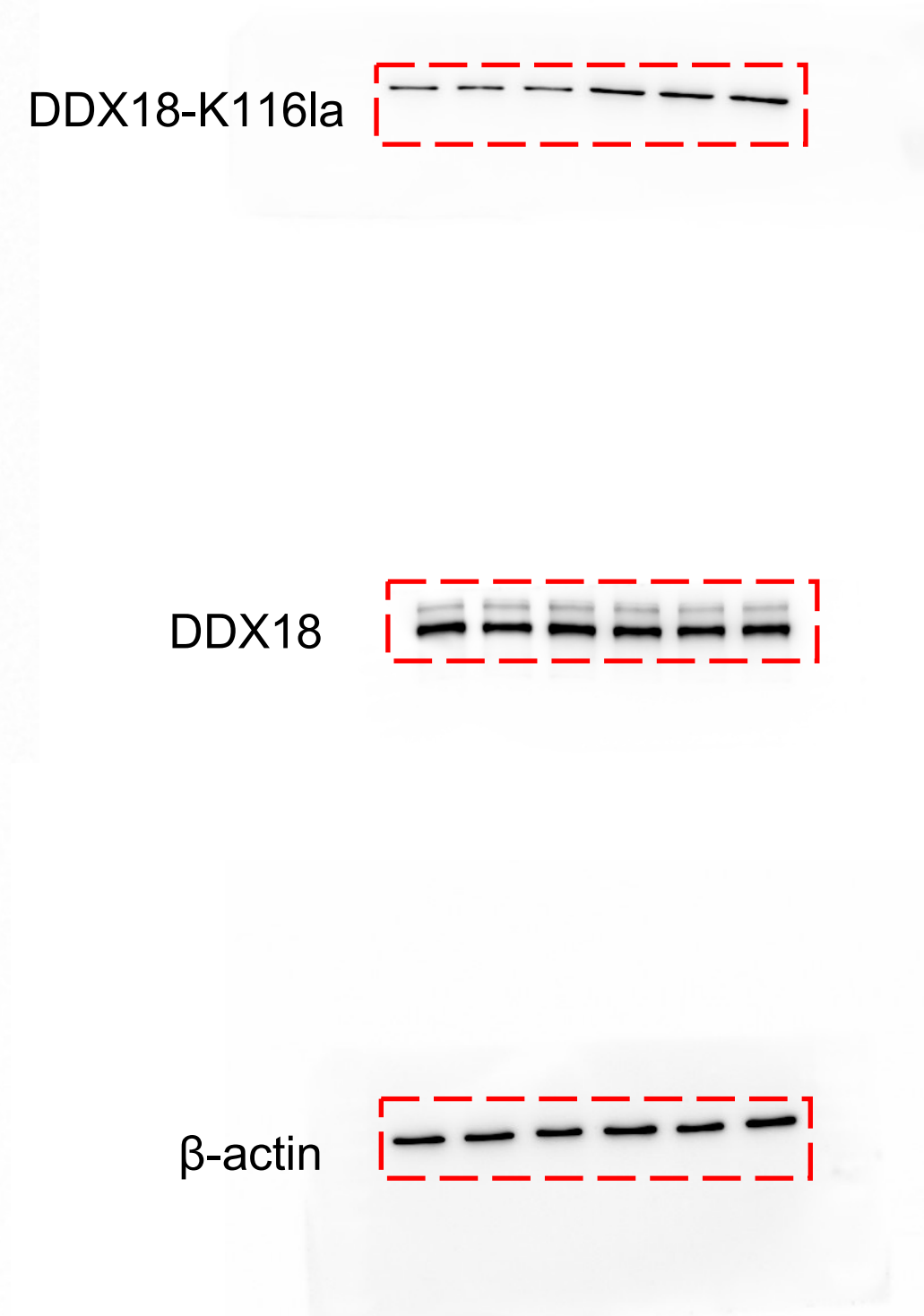

Full unedited gel for Figure 5E

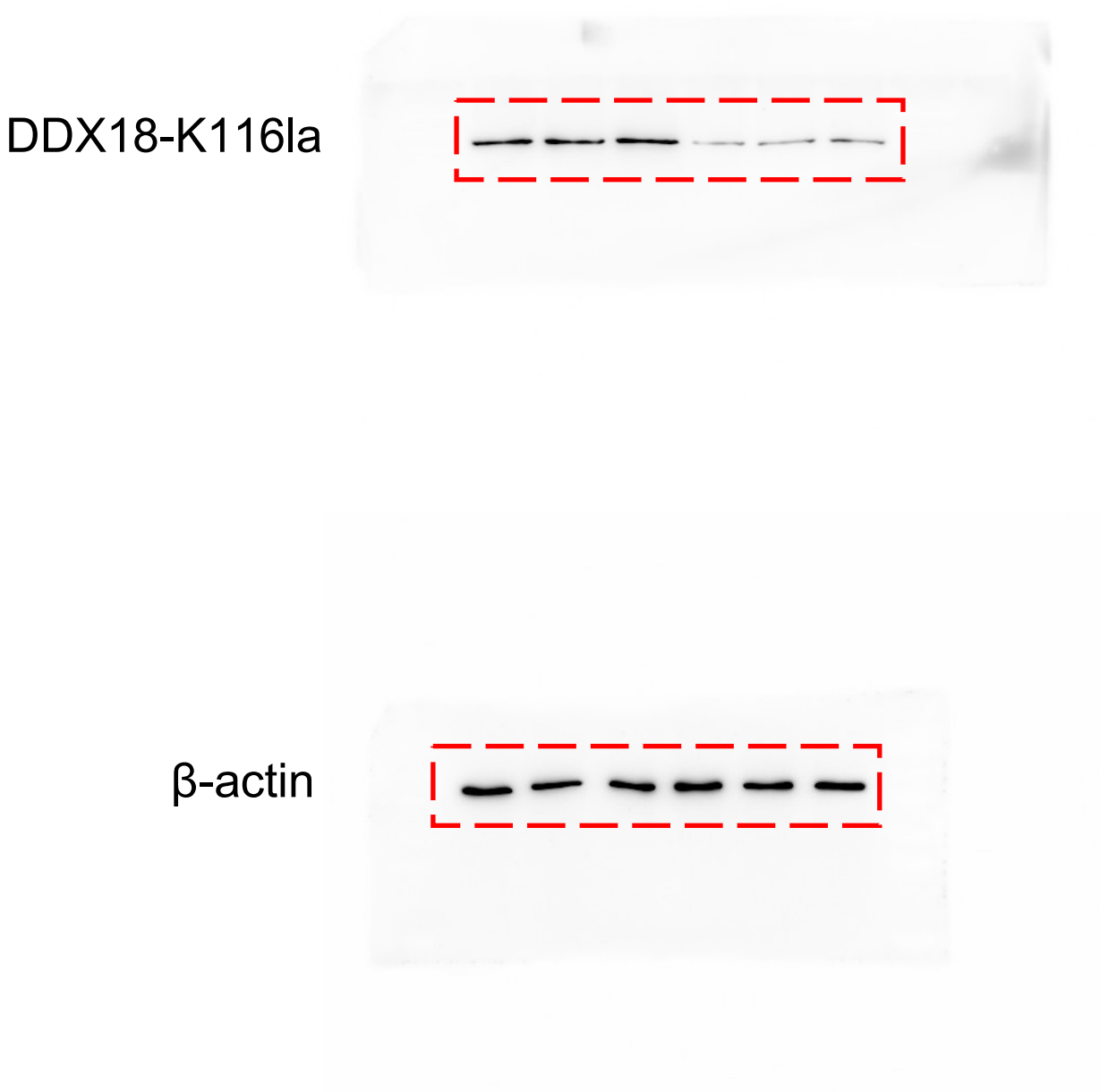

Full unedited gel for Figure 5F

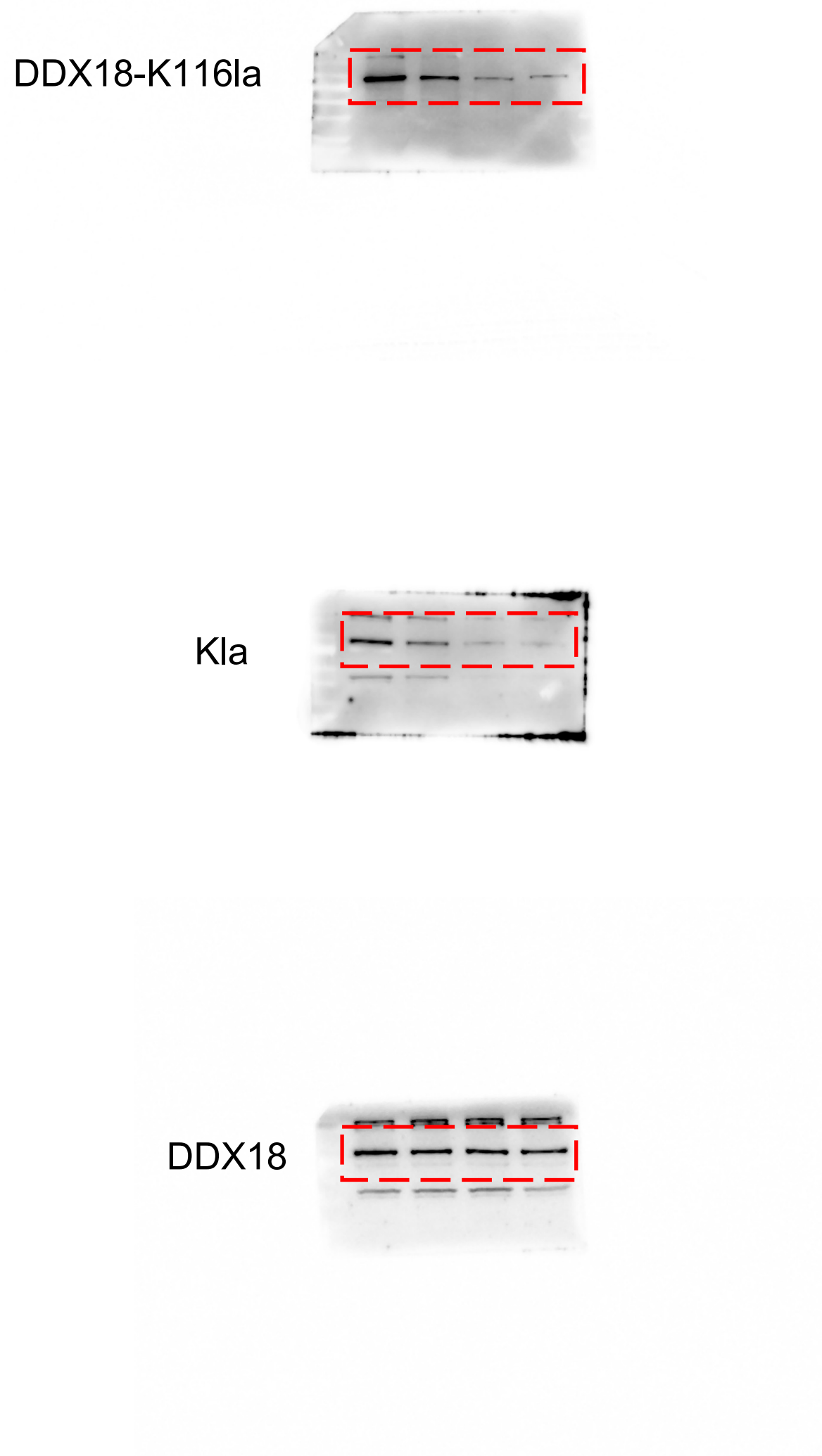

Full unedited gel for Figure 5I

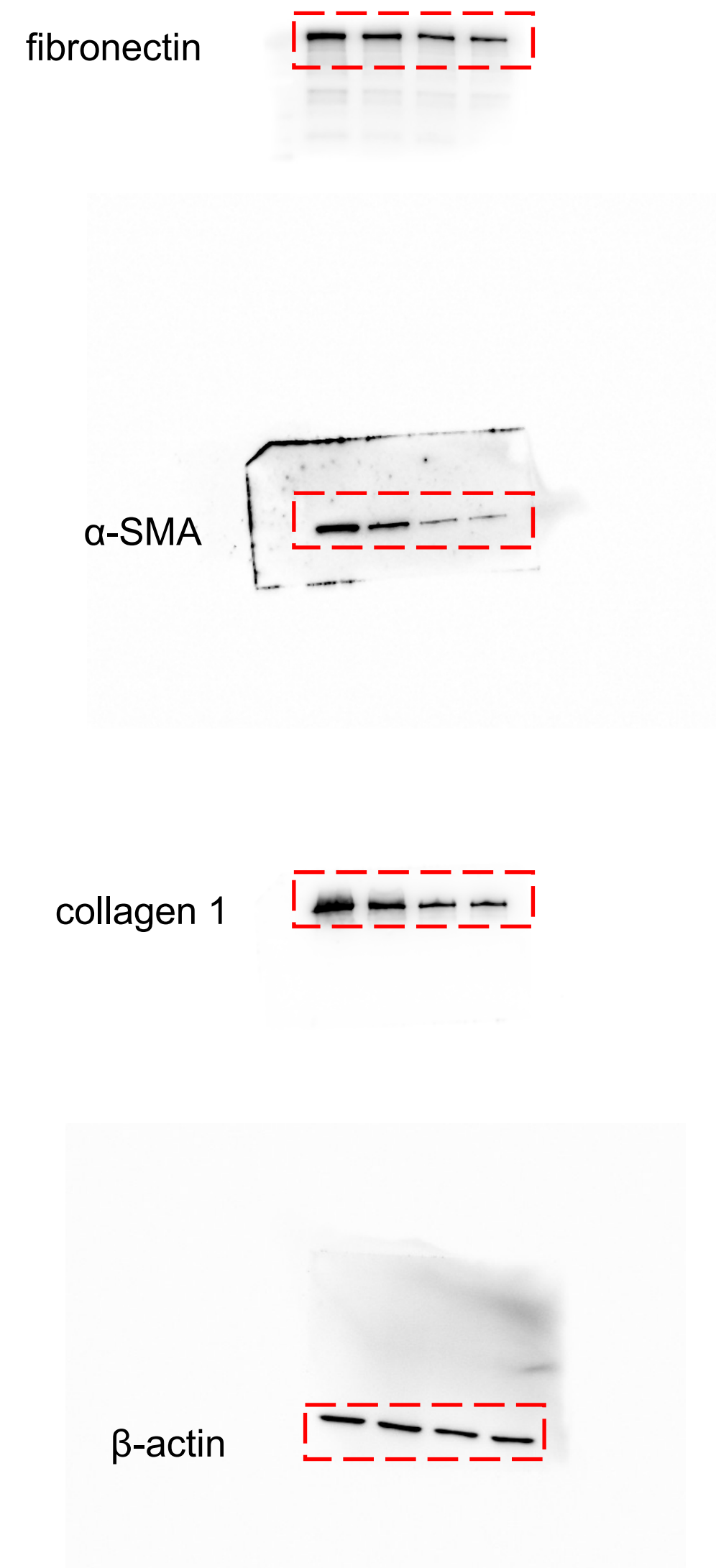

Full unedited gel for Figure 6I

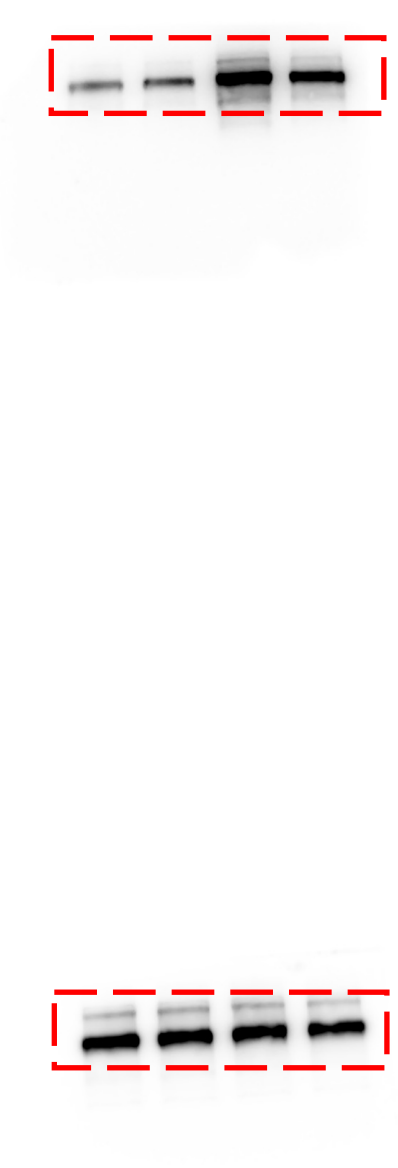

Full unedited gel for Figure 6L

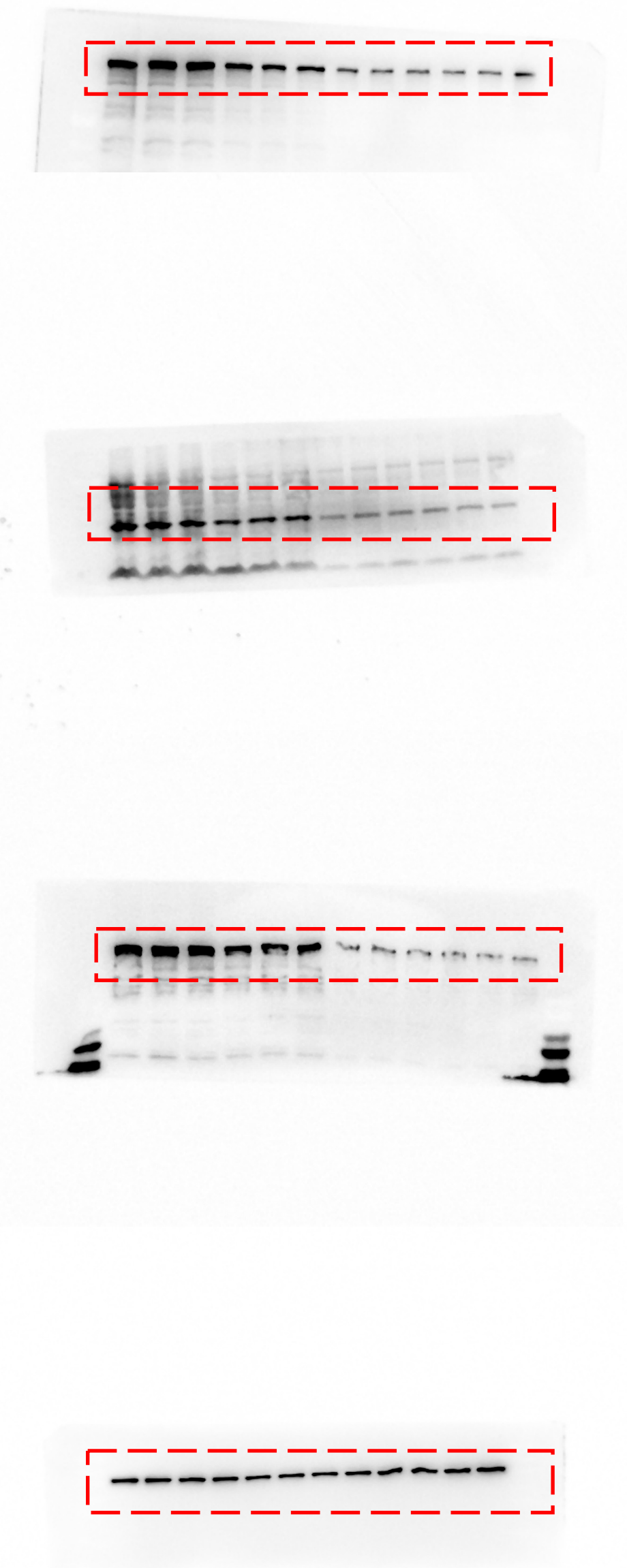

Full unedited gel for Figure 6P

DDX18

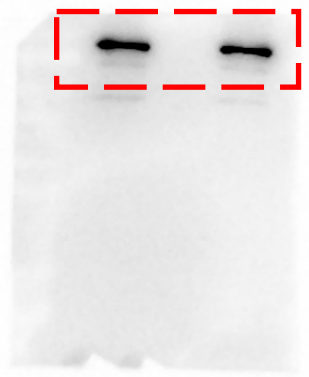

DDX18

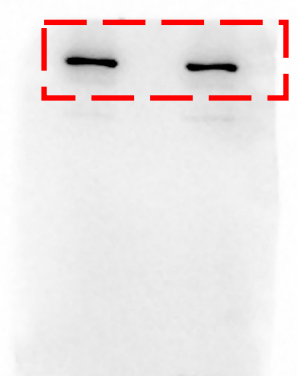

Full unedited gel for Figure 6Q

CD44

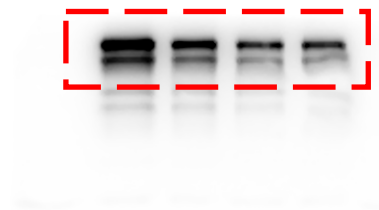

β-actin

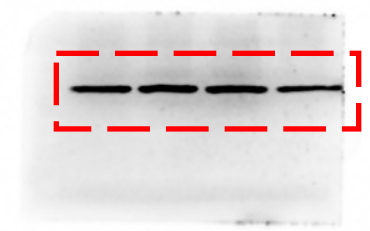

Full unedited gel for Figure 7B

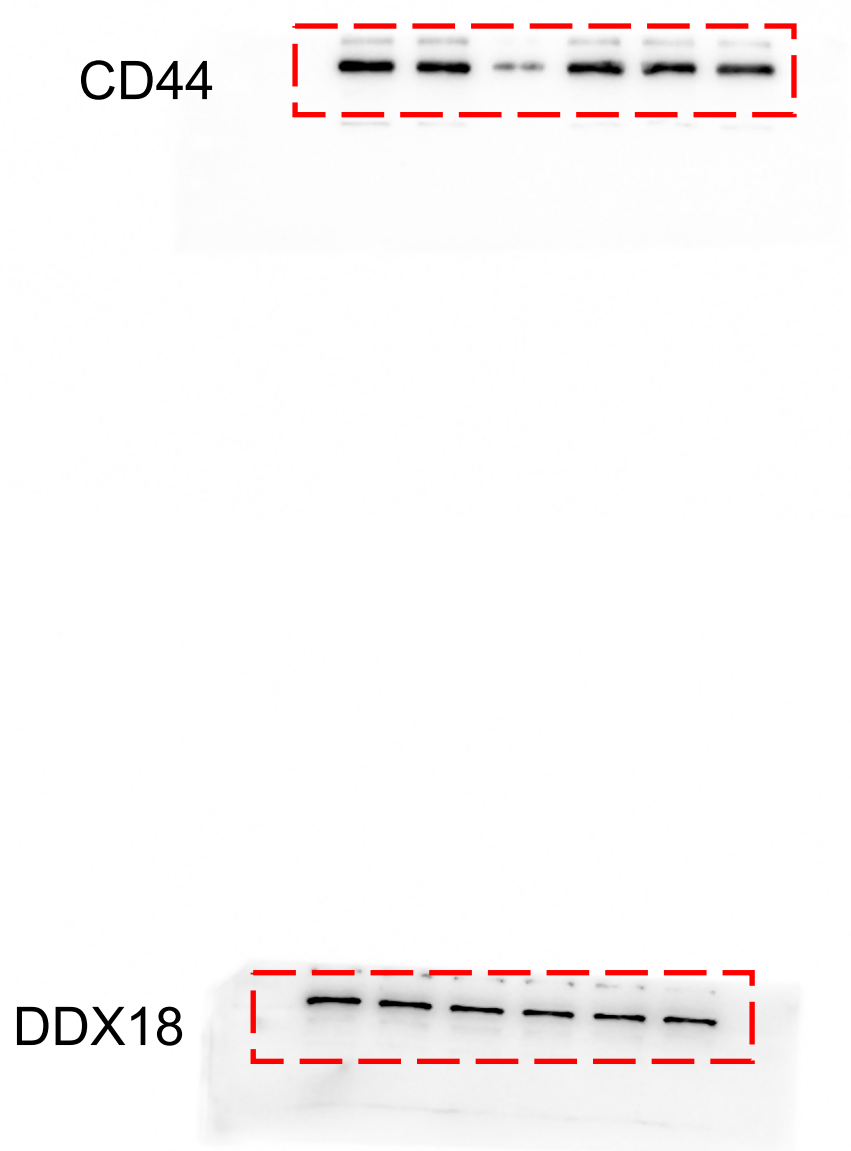

Full unedited gel for Figure 7D

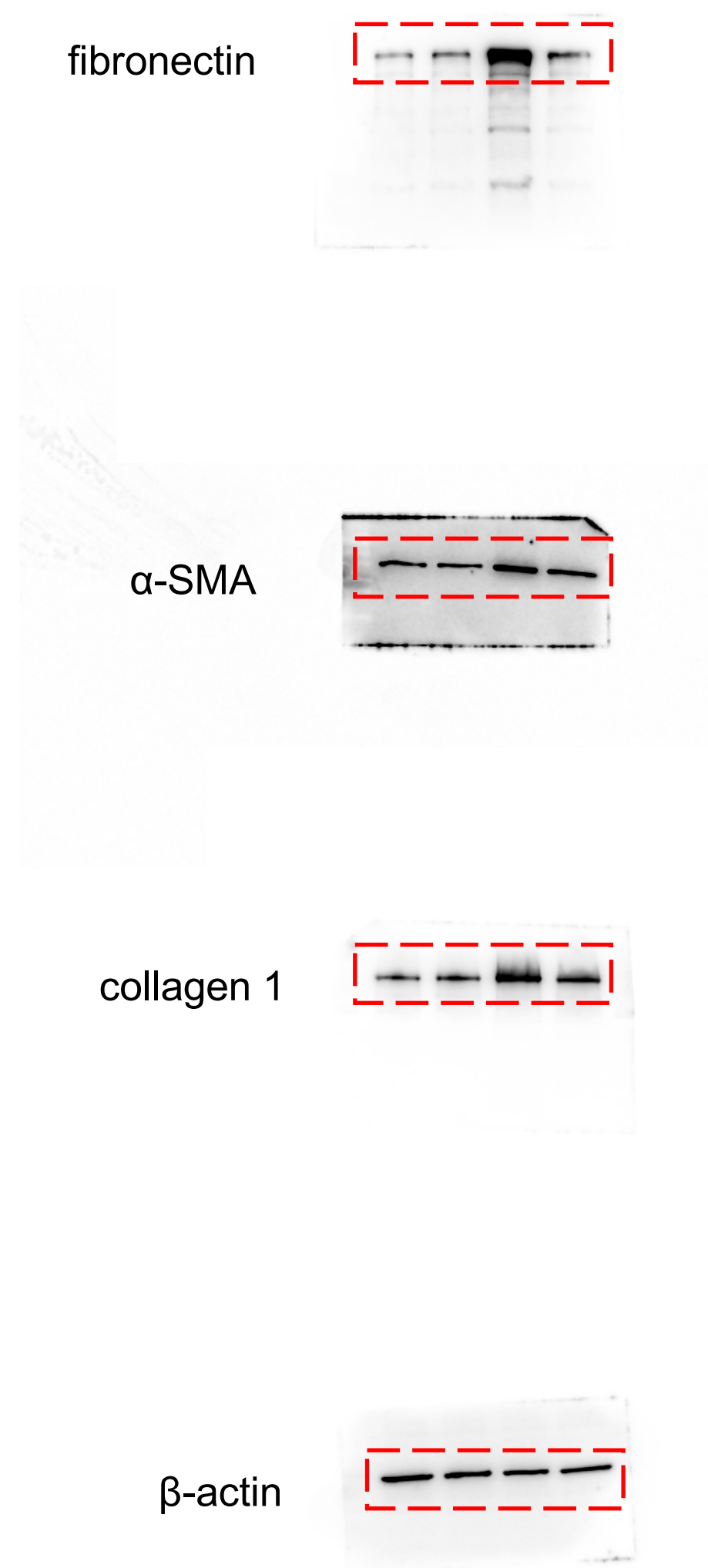

Full unedited gel for Figure 7L

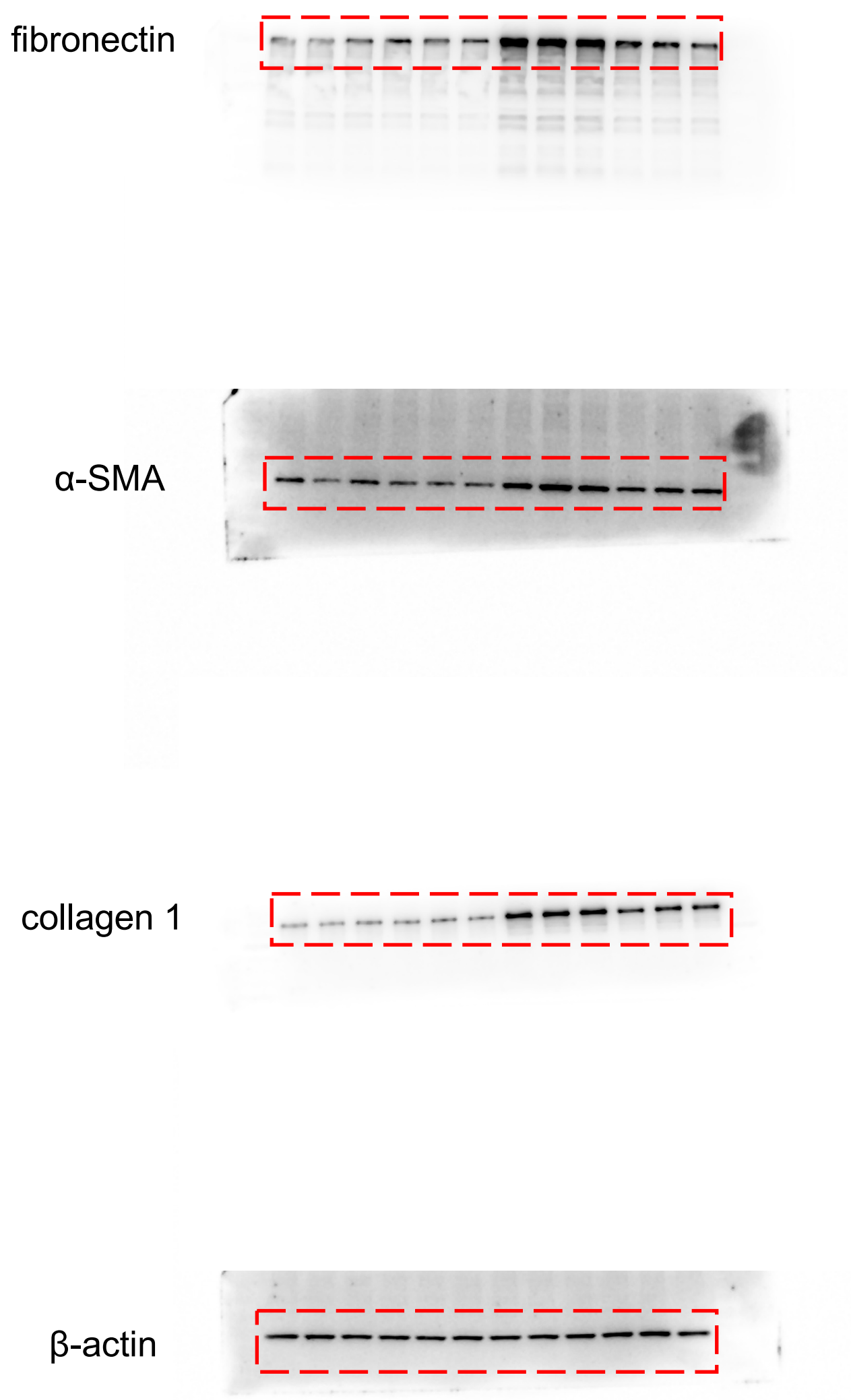

Full unedited gel for Figure 7O

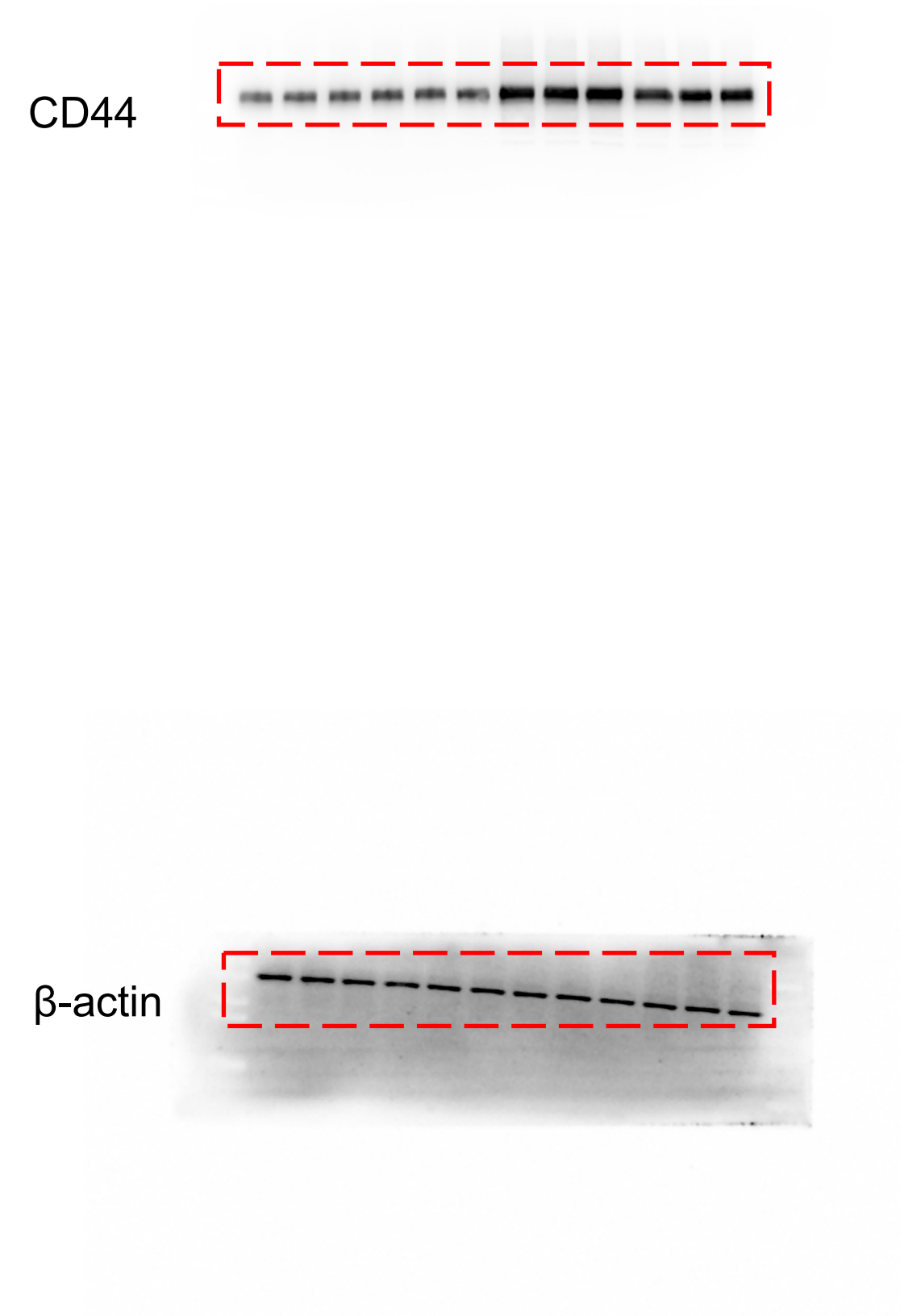

Full unedited gel for Supplementary Figure 1E

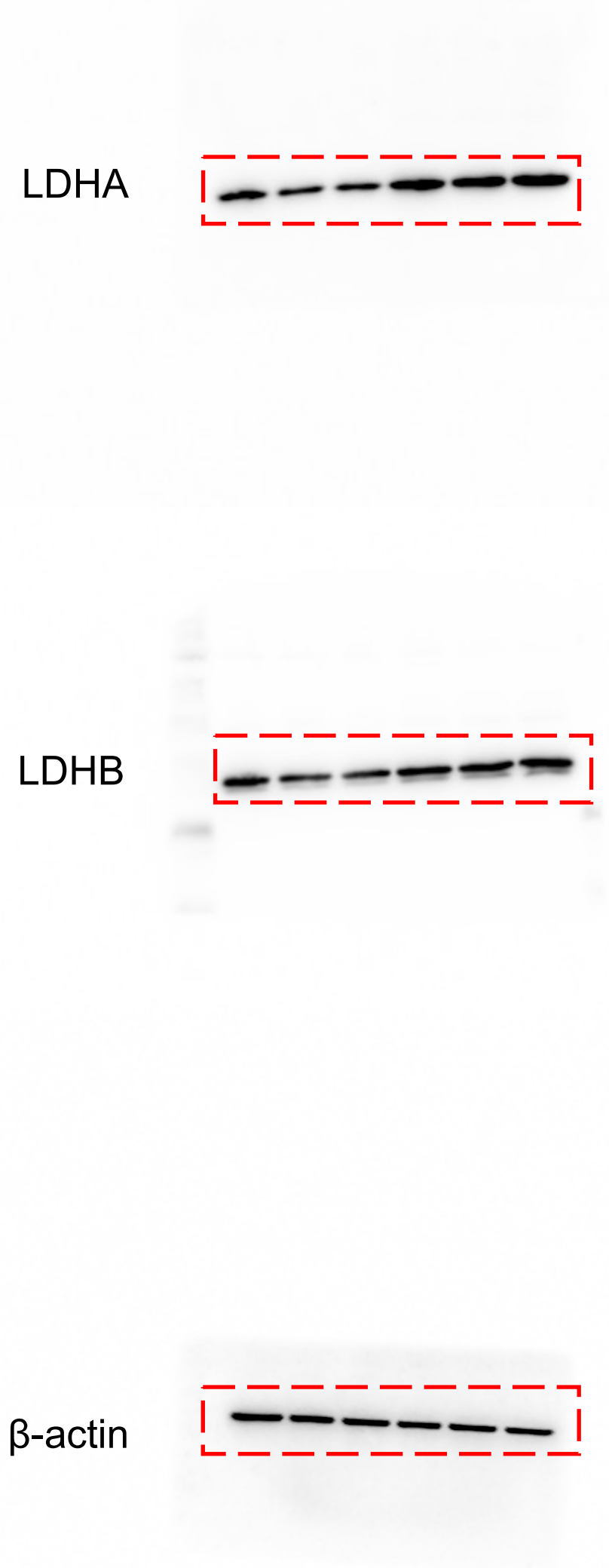

Full unedited gel for Supplementary Figure 1H

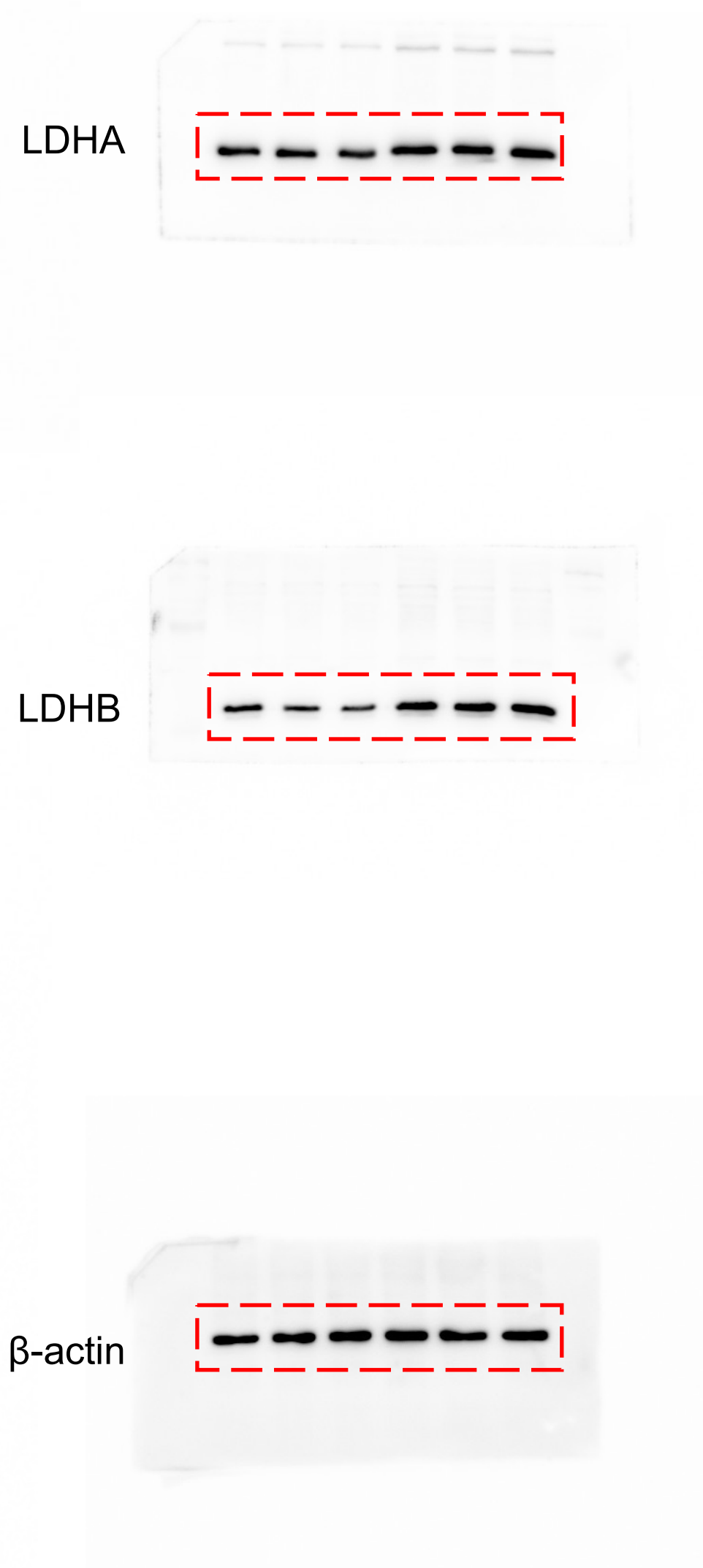

Full unedited gel for Supplementary Figure 1M

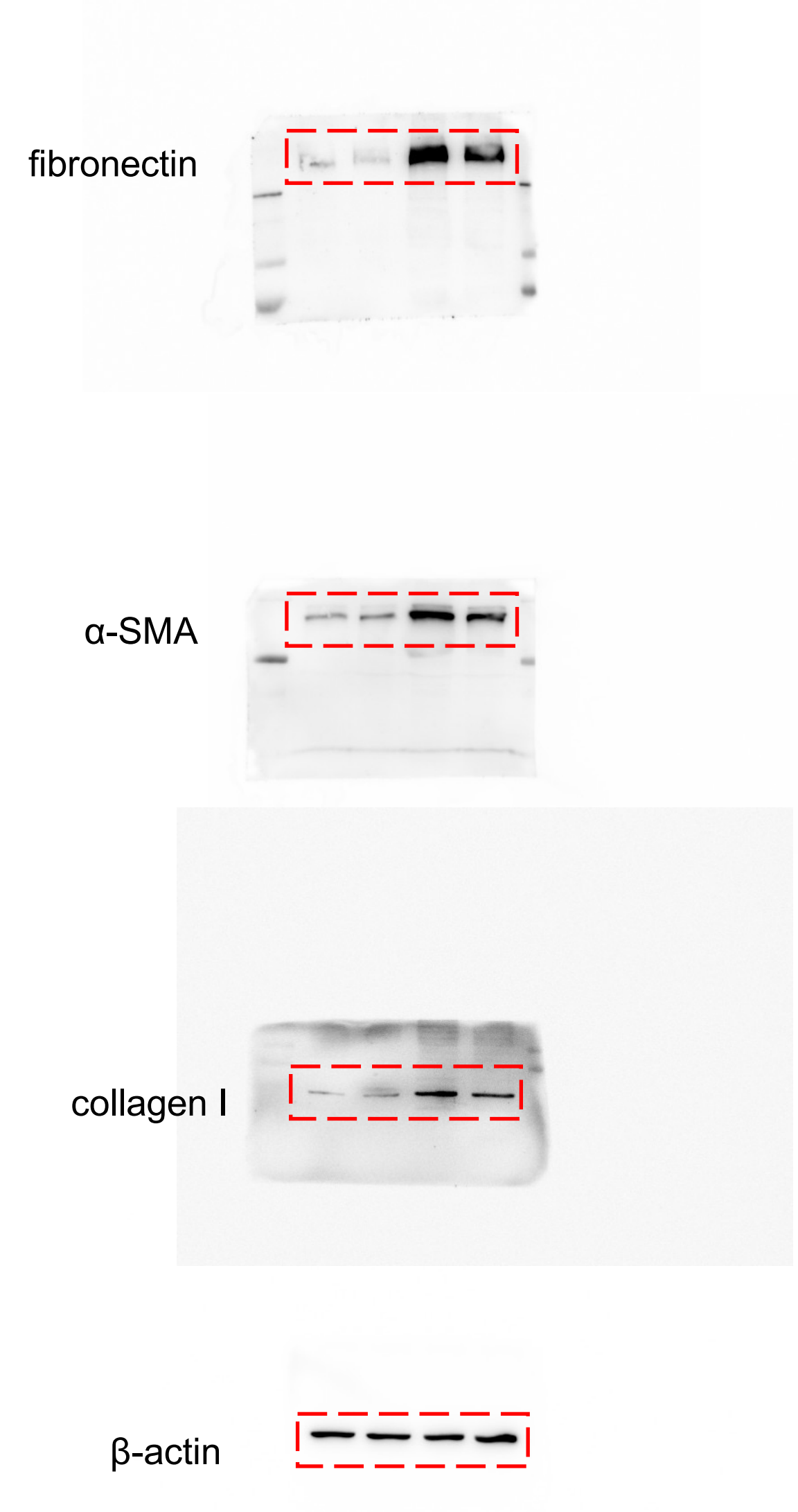

Full unedited gel for
Supplementary Figure 4A

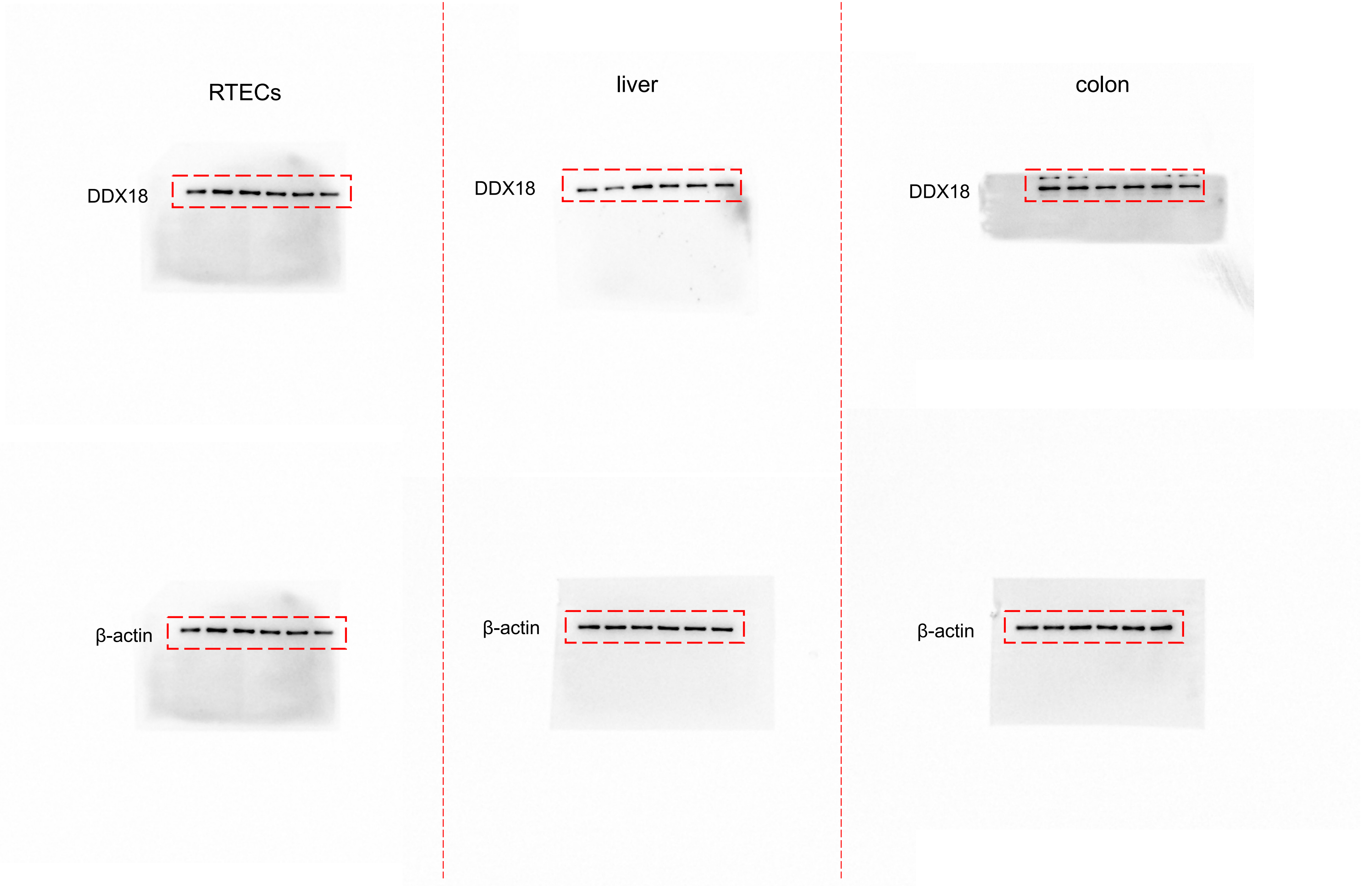

Full unedited gel for
Supplementary Figure 7B

Lactylated peptide Non-lactylated peptide

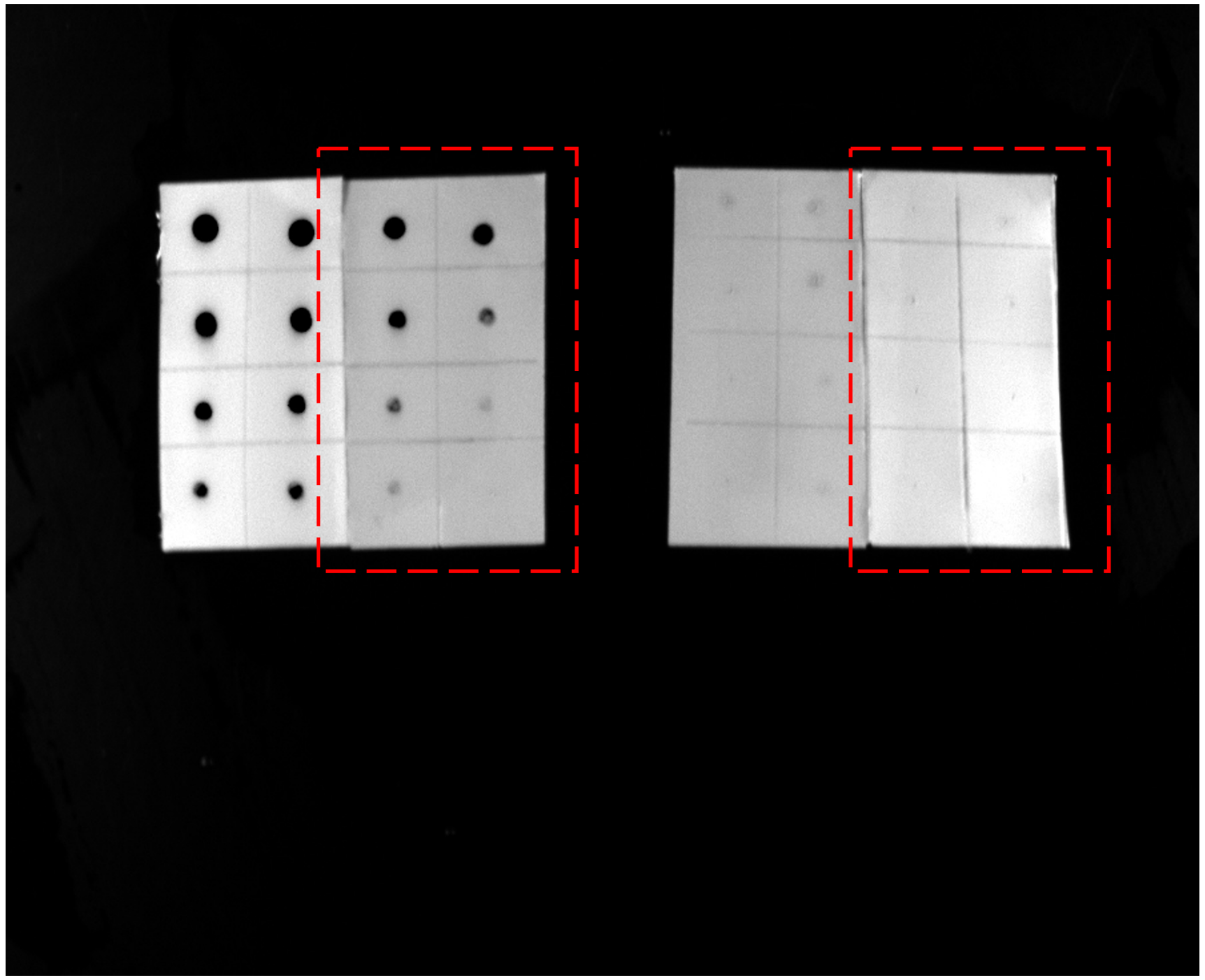

Full unedited gel for Supplementary Figure 8C

DDX18-K116la

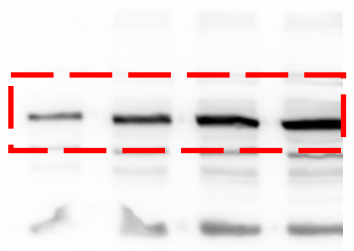

DDX18

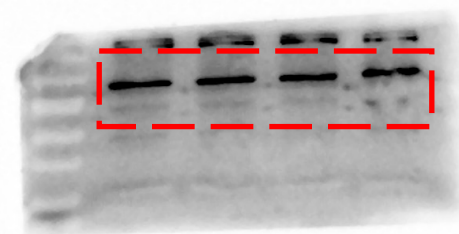

Full unedited gel for Supplementary Figure 8F

DDX18-K116la

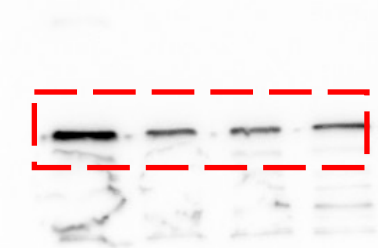

DDX18

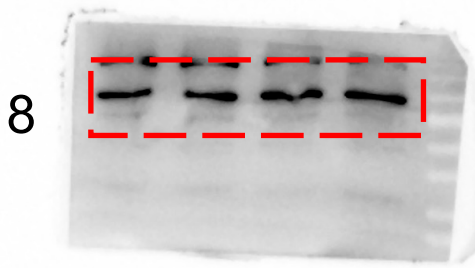

Full unedited gel for Supplementary Figure 8D

DDX18-K116la

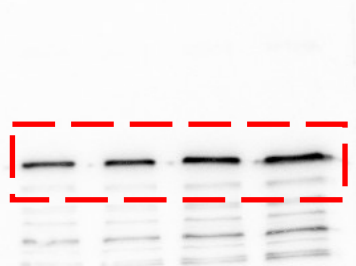

DDX18

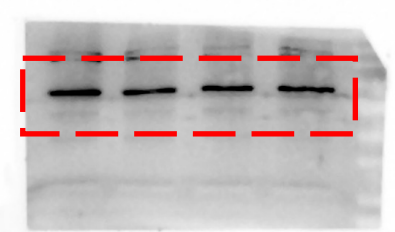

Full unedited gel for Supplementary Figure 8G

DDX18-K116la

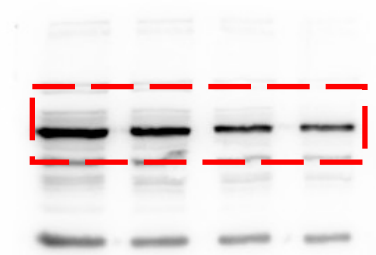

DDX18

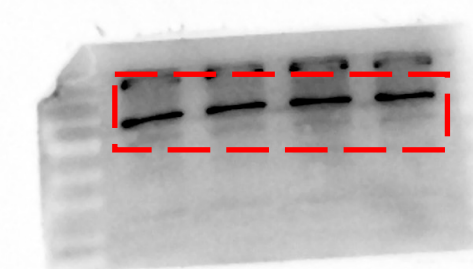

Full unedited gel for Supplementary Figure 8E

DDX18-K116la

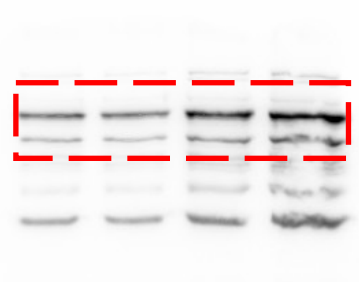

DDX18

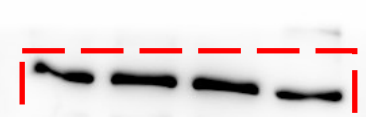

Full unedited gel for Supplementary Figure 9C

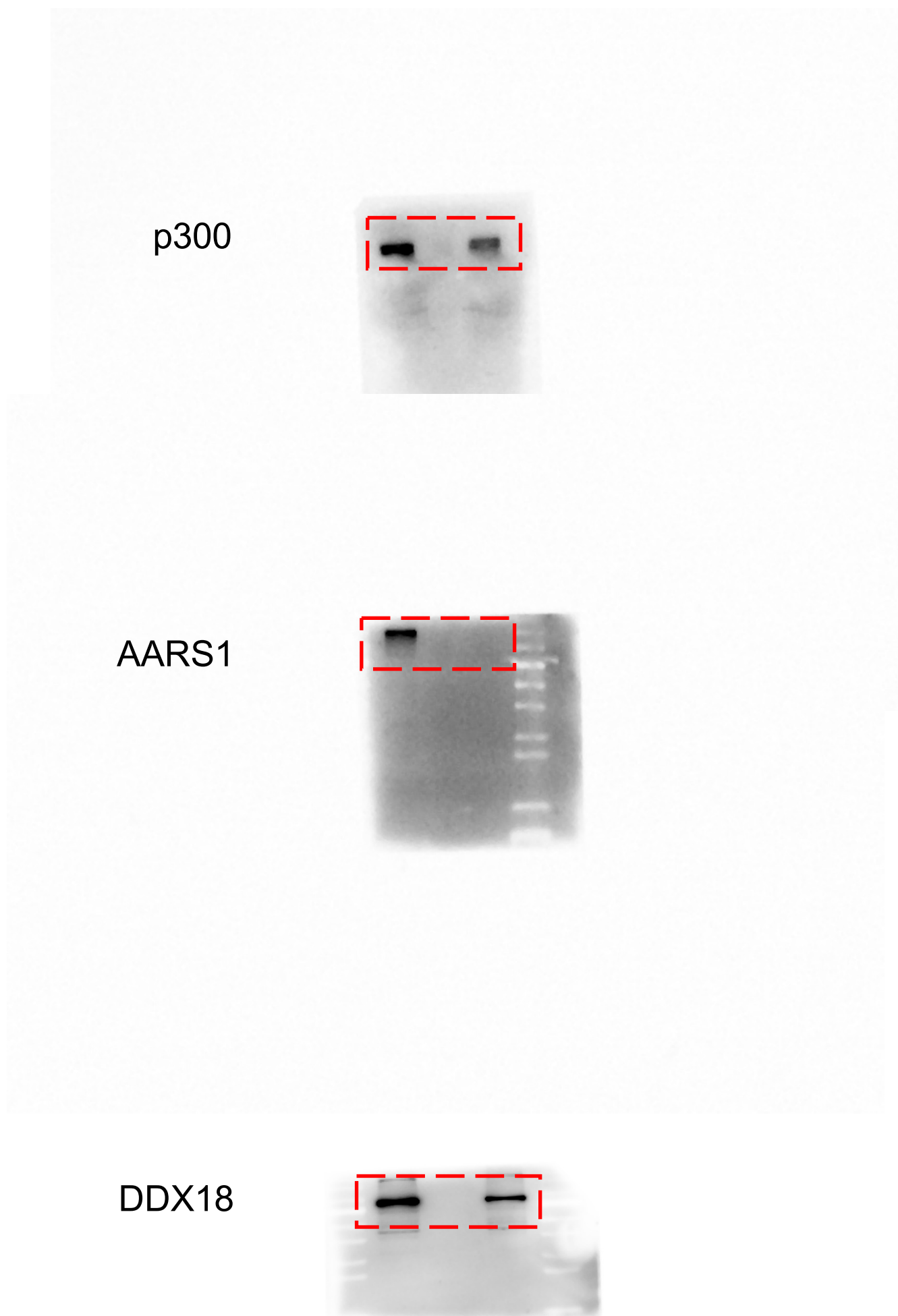

Full unedited gel for Supplementary Figure 9D

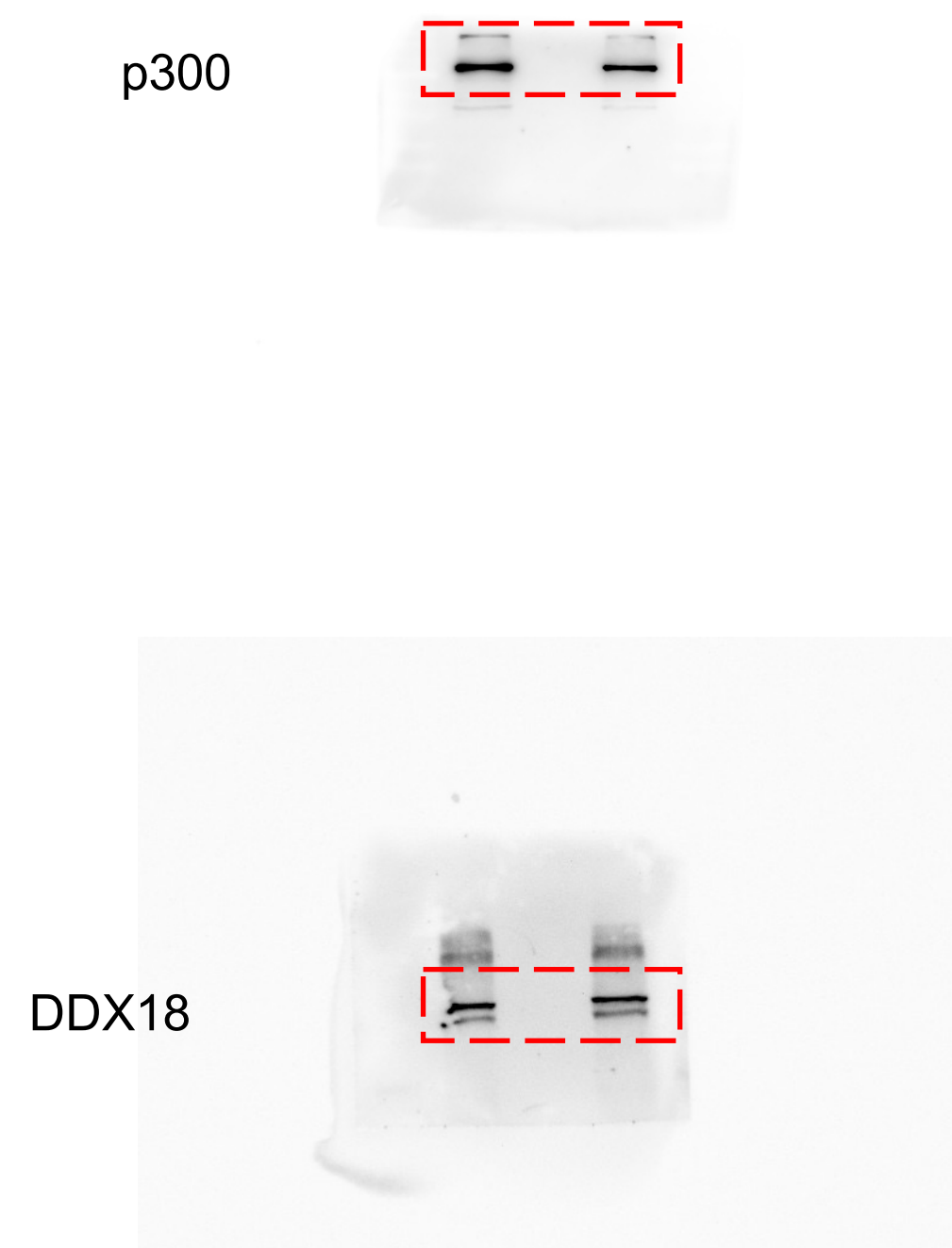

Full unedited gel for
Supplementary Figure 10B

fibronectin

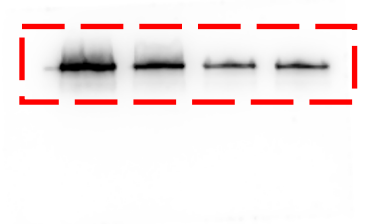

α-SMA

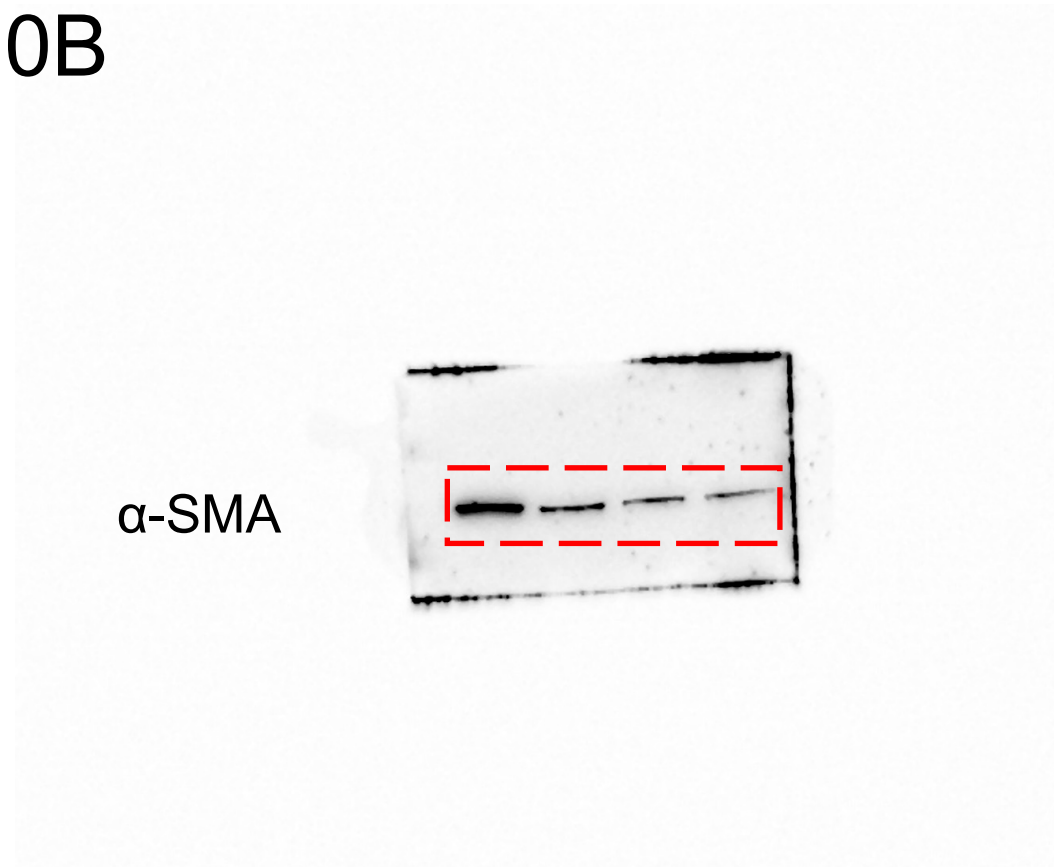

collagen 1

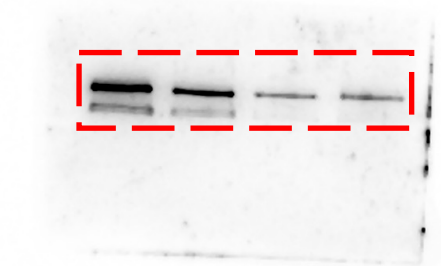

CD44

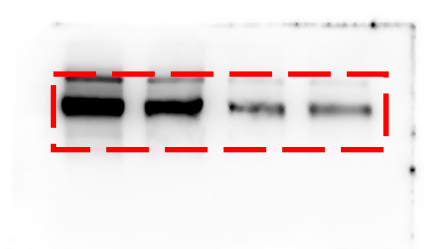

β-actin

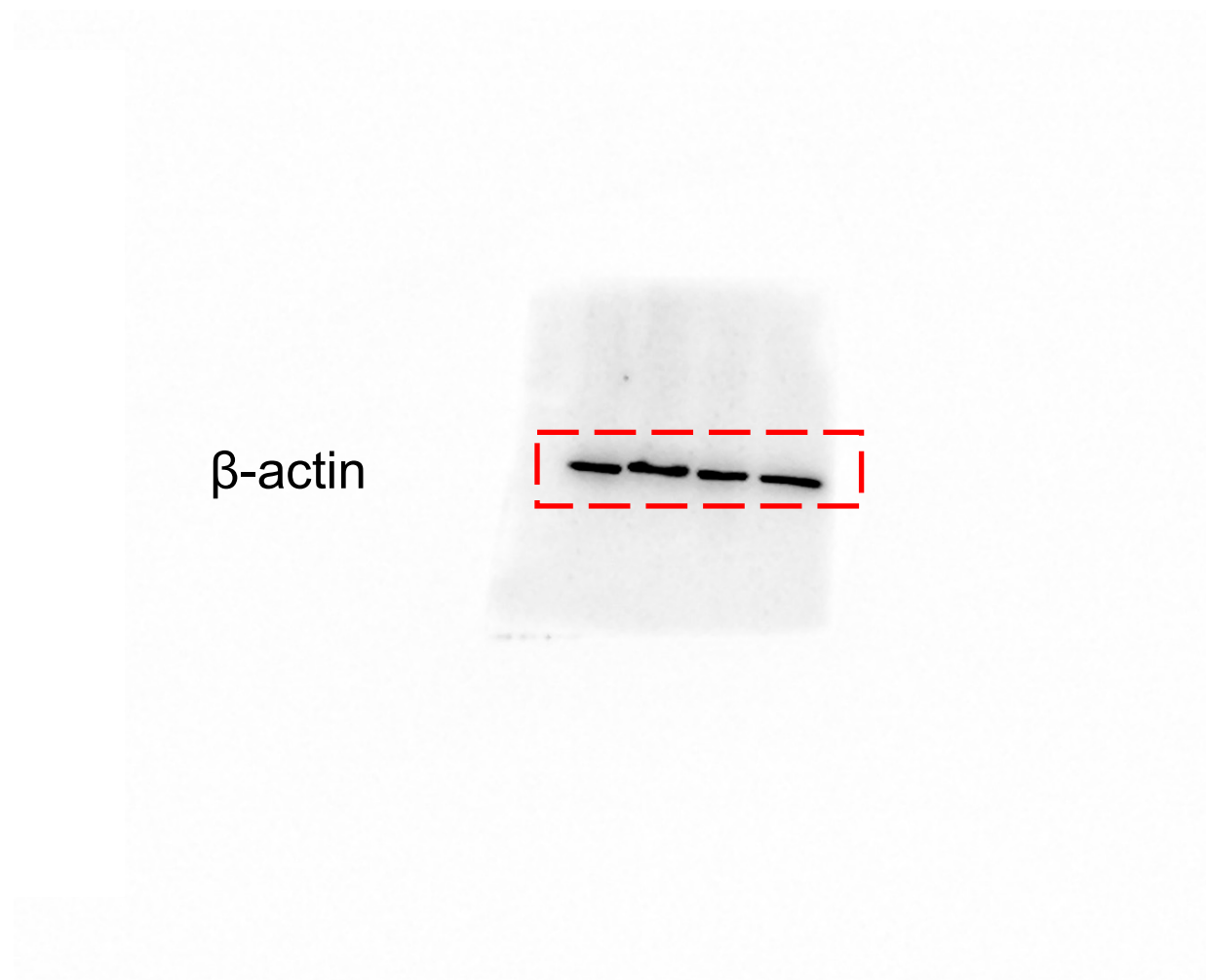

Supplement: Supplementary file 2 [file jci-136-201848-s331.pdf]
